# Prolyl-isomerase Pin1 drives platinum resistance by regulating Notch3 stability and function in ovarian cancer

**DOI:** 10.1186/s13046-026-03658-x

**Published:** 2026-02-11

**Authors:** Maria Valeria Giuli, Angelica Mancusi, Bianca Natiello, Samuele Di Cristofano, Rebecca Reali, Maria Gemma Pignataro, Daniel D’Andrea, Laura Di Magno, Carmine Nicoletti, Alessandra Giorgi, Alberto Macone, Serena Camerini, Marialuisa Casella, Giovanna Peruzzi, Sabrina Zema, Gianluca Canettieri, Federica Tomao, Innocenza Palaia, Angelina Pernazza, Alessandra Rustighi, Rocco Palermo, Domenico Raimondo, Alessandra Monti, Nunzianna Doti, Giulia d’Amati, Giannino Del Sal, Isabella Screpanti, Claudio Talora, Diana Bellavia, Saula Checquolo

**Affiliations:** 1 Department of Medico-Surgical Sciences and Biotechnology, Sapienza University of Rome, Laboratory affiliated with Istituto Pasteur Italia- Fondazione Cenci Bolognetti, Latina, Italy; 2 Department of Pediatric Hematology and Oncology, Cell and Gene Therapy, Bambino Gesù Children’s Hospital, IRCCS, Rome, Italy; 3 Department of Molecular Medicine, Sapienza University of Rome, Laboratory affiliated with Istituto Pasteur Italia- Fondazione Cenci Bolognetti, Rome, Italy; 4 Department of Radiological, Oncological and Pathological Sciences, Sapienza University of Rome, Rome, Italy; 5 School of Engineering Mathematics and Technology, University of Bristol, Bristol, UK; 6 Department of Anatomy, Histology, Forensic Medicine and Orthopaedics, Sapienza University of Rome, Rome, Italy; 7 Department of Biochemical Sciences, Sapienza University of Rome, Rome, Italy; 8 Core Facilities, Istituto Superiore di Sanità, Rome, Italy; 9 Center for Life Nano- & Neuro-Science@Sapienza, Istituto Italiano di Tecnologia, Rome, Italy; 10 Department of Gynecological, Obstetrical and Urological Sciences, Sapienza University of Rome, Rome, Italy; 11 Department of Life Sciences, University of Trieste, Trieste, Italy; 12 International Centre for Genetic Engineering and Biotechnology (ICGEB), Area Science Park-Padriciano, Trieste, Italy; 13 Istituto di Biostrutture e Bioimmagini, IBB-CNR, Naples, Italy; 14 AIRC Institute of Molecular Oncology, IFOM ETS, Milan, Italy

**Keywords:** HGSOC, Pin1 targeting, Notch3, Platinum drug resistance

## Abstract

**Background:**

Resistance to platinum-based drugs represents a major obstacle for the management of high-grade serous ovarian cancer (HGSOC) patients. Indeed, the selective pressure of platinum-based (PT) chemotherapy often leads to the outgrowth of platinum-resistant subclones. In this scenario, the underlying adaptive networks should be fully investigated to provide advances toward more streamlined and personalized care.

**Methods:**

We conducted a comprehensive analysis of Pin1/Notch3relationship from HGSOC cell lines and primary tumours, integrating multiple genetic targeting under chemotherapy pressure, differential proteomic approaches, molecular docking data and dynamics simulations, thus identifying a functional circuit evaluated in vitro and in vivo models.We conducted a comprehensive analysis of relationship from HGSOC cell lines and primary tumours, integrating multiple genetic targeting under chemotherapy pressure, differential proteomic approaches, molecular docking data and dynamics simulations, thus identifying a functional circuit evaluated in vitro and in vivo models.

**Results:**

Here, we demonstrated that carboplatin treatment of HGSOC cells promoted the activation of the Pin1/Notch3 axis, resulting in platinum resistance. Accordingly, HGSOC-bearing patients showing increased Pin1/Notch3 co-expression after PT-based chemotherapy correlated with a clinical worse response. Conversely, genetic targeting of Pin1 combined with carboplatin treatment sensitizes resistant cells to platinum-based therapy, both in vitro and in vivo, strongly reducing their Notch3-mediated metastatic potential in preclinical murine models. Mechanistically, Pin1-Notch3 binding favours protection of Notch3 from its GSK3β-mediated degradation, resulting in increased Notch3 expression.

**Conclusions:**

Collectively, our findings identify the functional Pin1/Notch3 axis as an escape strategy from chemotherapy-induced cell death, thus suggesting a novel predictive role of the Pin1/Notch3 axis in the platinum response, which could be useful for implementing frontline treatments for HGSOC patients before recurrence.

**Supplementary Information:**

The online version contains supplementary material available at 10.1186/s13046-026-03658-x.

## Background

High-grade serous ovarian cancer (HGSOC) is the most common histological type of ovarian cancer (OC) and is characterized by a high degree of heterogeneity, which makes its classification and treatment challenging [1]. Furthermore, the lack of methods for early diagnosis and the absence of specific clinical manifestations at disease onset, together with recurrence and widespread dissemination into the peritoneum and organs located in the abdominal cavity, render it one of the most lethal female-related malignancies [2].

Currently, standard HGSOC treatment includes debulking surgery followed by platinum-based chemotherapy even if neoadjuvant chemotherapy before surgery has become a valid option [3]. Nevertheless, the prognosis remains poor, given that most patients relapse within two years with platinum-resistant disease [4]. Since drug resistance limits the effectiveness of cancer therapy, finding novel targetable biomarkers for predicting the response to platinum-based therapy is of paramount importance to support clinicians in the early selection of the optimal therapy and to foster more effective therapeutic approaches to reverse platinum-based drug resistance [5].

The evolutionarily conserved Notch signalling pathway has emerged as a promising candidate given its multifaceted and well-documented role in tumorigenesis [6]. Overall, owing to the key role of Notch signalling in the development of normal ovarian tissue as well as in the carcinogenesis and tumour progression of OC [7], an increasing number of studies have focused on the involvement of Notch signalling in the promotion of drug resistance in OC [8, 9]. Among the four Notch paralogues encoded by the mammalian genome, Notch3 (N3) has been found to be altered in a wide panel of OCs [10]. Accumulating evidence has revealed its pivotal role in supporting OC stem cells [11] and platinum resistance [12]; hence, evaluating the efficacy of N3-specific inactivation to restore chemosensitivity in HGSOC [13] is strongly warranted.

Notably, given that Notch signalling plays a key role in tumoral, stromal, and immune cell compartments, as well as in healthy tissues, pan-Notch inhibition has led to off-target effects in several clinical trials [14]. Thus, research is moving towards Notch-specific targeted therapies even though numerous shortcomings have also been identified in clinical trials [15], thereby highlighting the need to find novel strategies, including the modulation of positive regulators.

In this context, one promising candidate for fine-tuning N3 might be the peptidyl-prolyl *cis/trans* isomerase Pin1, which is overexpressed in several cancers, including OC [16]. By binding and catalyzing the *cis/trans* conversion of specific motifs, Pin1 regulates the activity of a plethora of cancer-driving pathways [17], including those involving Notch receptors [18]. Indeed, we previously demonstrated that Pin1 positively regulates the N3-dependent aggressive properties of T-cell acute lymphoblastic leukemia (T-ALL) by increasing N3 stability [19]. In addition, Pin1 KO or pharmacological inhibition has been shown to curb tumour growth, metastasis, and chemoresistance in several types of cancers [18, 20]. However, whether Pin1 inhibition holds promise in impairing N3 signalling and chemoresistance in HGSOC remains to be elucidated.

Here, we show that Pin1 is able to bind the N3 protein, thus increasing its stability following platinum treatment of HGSOC cells, ultimately resulting in the acquisition of platinum resistance. At the biochemical level, the residues involved in Pin1/N3 binding have been identified, and the atomic interaction between these two proteins has been studied via molecular docking and molecular dynamics approaches. Mechanistically, we demonstrated that this binding interferes with the GSK3β-dependent phosphorylation of N3, thus resulting in the impairment of its proteasomal degradation. Notably, while immunohistochemical analyses of primary tumours revealed an interesting positive correlation between Pin1 and N3 protein expression in human patients, genetic inhibition of Pin1 in preclinical models impaired N3 signalling and resulted in sensitivity to platinum agents both in vitro and in vivo. Collectively, these findings reveal the intriguing possibility of combining platinum-based chemotherapy with Pin inhibition to overcome N3-mediated platinum resistance.

## Methods

### Cell cultures, transfections, and lentiviral infections

HEK293T (purchased from ATCC), HEK293T-Pin1KO (kindly provided by Prof. G. Del Sal [21]) and Caov3 (kindly provided by Prof L. Rosanò, CNR Rome) cells were maintained in Dulbecco’s modified Eagle’s medium (DMEM) (#SD6546-500 mL; Sigma‒Aldrich, St. Louis, MO, USA) supplemented with 10% fetal bovine serum (FBS) (Gibco, Carlsbad, CA, USA) and 2 mM glutamine (#G7513-100 mL; Sigma‒Aldrich). Kuramochi cells (kindly provided by Prof. S. Indraccolo) were cultured in RPMI-1640 (#R0883-500 mL – Sigma‒Aldrich) supplemented with 10% FBS (Gibco) and 2 mM glutamine. SKOV3 cells (purchased from ATCC) were cultured in McCoy’s 5 A medium (Merck, Kenilworth, NJ, USA) supplemented with 10% FBS (Gibco) and 2 mM glutamine.

Caov3 CBDCA-resistant cells were generated as previously described [22]. Briefly, we treated Caov3 cells for 2 h with a CBDCA dose 10-fold greater than the IC_50_, followed by a recovery period. After 16–20 cycles of CBDCA treatment, the resulting cell population was maintained in drug-free medium. We experimentally verified that the resistant phenotype was stable and maintained for at least two months, independently from the presence of CBDCA in the culture medium. The IC_50_ values were calculated in comparison with those in Caov3 parental cells.

Mycoplasma contamination in the cell cultures was routinely detected via a PCR detection kit (#ab289834; Abcam, Cambridge, UK).

Transient transfections were performed via Lipofectamine 2000 transfection reagent (#11668019 – Invitrogen - Thermo Fisher Scientific, Waltham, MA, USA) or Lipofectamine RNAiMAX transfection reagent (#13778-075 – Invitrogen – Thermo Fisher Scientific) in accordance with the manufacturer’s protocols.

For RNA interference, the cells were transfected with siRNA-Ctrl (#sc-37007), siRNA-Notch3 (#sc-37135), or siRNA-Pin1 (#sc-36230) (Santa Cruz Biotechnology, Dallas, TX, USA) for 72 h.

Unless otherwise specified, plasmids for lentiviral infection were purchased from Addgene (Watertown, MA, USA). SKOV3 cells were subjected to two lentiviral infections by combining packaging plasmids (pCMV and pMDG) with pLENTI-CMV-Puro-LUC (#17477) or a lentiviral construct encoding the entire human N3_ICD_ fragment [hN3_ICD_ (3xFLAG)-pCDF1-MCS2-EF1-copGFP] (#40640) and the corresponding empty control vector (#CD111B-1 – System Biosciences, Palo Alto, CA, USA). Viral supernatants were produced from HEK293T cells. After infection, pLENTI-CMV-Puro-LUC cells were selected via puromycin treatment (0.5µM/µL) (P7255; Sigma‒Aldrich). After infection with hN3_ICD_ (3xFLAG)-pCDF1-MCS2-EF1-copGFP] and the corresponding control vector, the GFP-transduced cells were subjected to fluorescence-activated cell sorting (FACS) and sorted on the basis of GFP expression via a FACSAria III (BD Biosciences, Franklin Lakes, NJ, USA) equipped with a 488 nm laser and FACSDiva software (BD Biosciences version 6.1.3). Briefly, cells were first gated on the basis of forward and side scatter areas (FSC-A and SSC-A), detected in the green fluorescence channel for GFP expression, and isolated on the basis of high GFP levels. Upon sorting, an aliquot of the collected cells was checked for purity (purity > 99%). For Kuramochi and Caov3 lentiviral infections, shPin1 was cloned and inserted into doxycycline-inducible pLKO-TetON (pLKO-TetO-shPin1), and pHAGE-GFP/LUC was kindly provided by Prof. G. Del Sal [21]. For the preparation of the viral particles psPAX2 and pMD2. G were used as packaging plasmids in HEK293T cells. After infection, pLKO-TetO-shPin1 cells were selected by puromycin treatment (1 µg/mL), whereas cells transduced with pHAGE-GFP/LUC were subjected to FACS analysis and sorted for GFP expression as previously described.

### Compounds and drug treatments

Where indicated, the cells were treated with MG132 (50µM for 4 h; #C2211-5MG; Sigma‒Aldrich), cycloheximide (CHX) (10 µg/ml for 0-2-4–6 h; C4859-1ML; Sigma‒Aldrich), Carboplatin (CBDCA) (C2538-100MG - Sigma-Aldrich), Cisplatin (CDDP) (#P4394 - Sigma-Aldrich), CHIR99021 (5µM for 2 h; SML1046-5MG; Sigma‒Aldrich), doxycycline hyclate (1 µg/mL for 48 h; D9891; Sigma‒Aldrich), and Paclitaxel (#33069-62-4 – Selleckchem, Houston, TX, USA) according to the manufacturers’ instructions.

For dose-response curves, the indicated cells [SKOV3_luc clones, HGSOC cells (including Caov3-resistant cells)] were seeded in 96-well culture plates and treated with: CBDCA (ranging from 0 to 1000µM) for 72 h and then released in drug-free medium for an additional 24 h; CDDP (ranging from 0 to 750µM) for 16 h and then released in drug-free medium for an additional 24 h; and Paclitaxel (ranging from 0 to 1µM) for 48 h. For dose-response curves on siRNA-transfected, and shRNA-transduced cells, increasing doses of CBDCA (ranging from 0 to 2000µM) were used for 16 h and then released in drug-free medium for an additional 24 h to better evaluate the effects of combined treatments studies [23]. Cell viability was determined via a CellTiter 96 AQueous kit (#G3582 - Promega, Fitchburg, WI, USA). The absorbance was detected at 492 nm via a Glomax Discover Microplate Reader (Promega). The data were analysed as previously described [24]. Briefly, data were collected as units of absorbance (ABS) and expressed as a percentage of viable cells with respect to untreated cells via the following equation: % Cell Viability = (ABScells+CBDCA – ABSmedium+compound)/(ABScells+H20 – ABSmedium+H20) X 100. The results are presented as the means ± SDs of three experiments, each performed in triplicate.

In the experiments with a fixed dose of CBDCA, cells were treated with a suboptimal concentration referred to approximatively 25% reduction in cell viability (IC_25_) for the time indicated in each figure legend.

### Immunoblot and Immunoprecipitation analyses

The cells were lysed in a mixture containing lysis buffer (50 mM Tris HCl (pH 7.5), 150 mM NaCl, 1 mM EDTA, 0.5% Triton X-100, 10 mM NaF, 1 mM Na_3_VO_4_, 1 mM PMFS, and 1% protease inhibitors) and clarified at 13.000 × rpm for 15 min at 4 °C [25]. Before immunoblotting, the samples were mixed with β-mercaptoethanol (#M6250; Sigma‒Aldrich) and Laemmli Sample Buffer (#1610737; Bio-Rad, Hercules, CA, USA) and boiled for 5 min at 99 °C. For immunoblotting, protein extracts were run on SDS‒polyacrylamide gels and transferred to nitrocellulose membranes (#1620115; Bio-Rad).

Immunoprecipitation was performed using whole-cell extracts. For Pin1 and GSK3β immunoprecipitation, the cell lysates were immunoprecipitated overnight at 4 °C with rotation with specific primary antibodies or IgG as a negative control (#sc-2025; Santa Cruz Biotechnology) and then incubated with Protein A/G-agarose beads (#sc-2003; Santa Cruz Biotechnology) for 1 h at 4 °C with rotation. For FLAG and HA immunoprecipitation, the cell lysates were incubated for 2 h at 4 °C with rotation with anti-FLAG M2 affinity gel or anti-HA agarose conjugate (HA 7) beads, respectively (Table S2). Where specified, the FLAG peptide (#F3290; Sigma‒Aldrich) was used as the negative control.

The immunoprecipitated proteins were then washed five to eight times with CO-IB buffer (50 mM Tris HCl (pH 7.5), 150 mM NaCl, 15 mM EDTA, 0.5% Triton X-100), resuspended in Laemmli sample buffer (#1610737 – Bio-Rad), boiled for 5 min at 99 °C, resolved via SDS‒PAGE and then subjected to immunoblot analysis by using antibodies listed in Table S2.

All immunoblotting data are representative of three independent experiments.

### Hematoxylin and Eosin (HE) and immunohistochemistry

Human and murine tissue samples were fixed and paraffinized as described. Three slides were prepared with 4 μm section from the paraffin-embedded tissues. One sample was stained with hematoxylin‒eosin to evaluate the morphology of the tissue and the presence of tumour cells. The other sections were stained with rabbit anti-Notch3 and mouse anti-Pin1 antibodies (listed in Table S2). Antigen retrieval was performed in 10 mM sodium citrate buffer (pH 6.0) for 15 min for Pin1 and 20 min for Notch3 in microwave. After they were incubated with primary antibodies, the sections were washed and incubated with secondary biotinylated antibodies. Antibody binding was detected with a DAB staining system (SK-4105 Vector - Vector Laboratories, Newark, CA, USA) according to the manufacturer’s protocol. Where indicated, immunohistochemical staining of N3 and Pin1 was converted to an H score [intensity (0, 1, 2, 3) × area (0–100%)] as previously described [16]. The H score values in the plot represent the means of five independent fields for each sample.

### Study population

Our *in-house* dataset consists of 62 HGSOC samples selected from our institutional tissue bank (Department of Maternal Child and Urological Sciences, Policlinico Umberto I, “Sapienza” University of Rome). Biospecimens were collected from newly diagnosed patients with HGSOC (age ≥ 18 years) who underwent surgical resection and had received no prior treatment for their disease, including chemotherapy or radiotherapy, from 2015 to 2024. Patients were stratified into “High” (H) and “Low” (L) expression groups based on the IHC H score values for both N3 and Pin1 proteins and using the lower tertile for each protein as threshold (H > cut-off value vs. L ≤ cut-off value).

The paired tumour samples (pre-NACT and post-NACT), derived from our institutional tissue bank, were obtained from newly diagnosed patients with HGSOC (age ≥ 18 years) who underwent NACT followed by interval debulking surgery (IDS). Pre-NACT tumour biospecimens were acquired through diagnostic laparoscopy, while post-NACT biospecimens were collected during IDS, between 2018 and 2023. Stratification of these samples was performed by using the same cut-off values described above.

For the institutional tissue bank, all patients had previously provided written informed consent indicating their authorization or refusal for the collection, storage, and research use of biological and tissue samples.

Progression-Free Interval (PFI) data and clinical information for HGSOC patients (*n* = 150) were derived from an external public dataset (https://cptac-data-portal.georgetown.edu) named *Zhang et al.* [26]. The analysis included patients for whom survival data were available and who had a minimum follow-up of 3 months.

Patients were stratified into two groups, “High Pin1 and Notch3” and “Low Pin1 and/or Notch3”, based on the expression levels of Pin/Notch3 proteins, with the lower tertile used as the threshold. The levels of Pin1 and N3 protein expression were downloaded from the Supplementary Information of the related paper [26]. In detail, patients with a protein expression level higher than the cut-off value in the cohort for both Pin1 and Notch3 were classified as “High Pin1 and Notch3”; the patients that were not included in the “High Pin1 and Notch3” group, were classified as “Low Pin1 and/or Notch3”.

Survival curves were estimated using the Kaplan–Meier method, and statistical differences were tested using the logrank test; P value < 0.05 was considered to be statistically significant.

### Generation of primary cancer cells from patient tissues

This study included HGSOC patients belonging to a prospectively enrolled cohort during 2024 and included under a prospective study protocol (n°0067/2024 – PI: Checquolo) approved by the ethical Committee of the Policlinico Umberto I (Rome, Italy). Patients provided specific written informed consent as part of their participation in the approved prospective study. Eligibility criteria: (1) histologically or cytologically confirmed HGSOC; (2) age ≥ 18 years; (3) no prior treatment for their disease, including chemotherapy or radiotherapy. Stratification of these samples was performed by using the same cut-off values described above for the *in-house* dataset.

HGSOC primary cells were obtained from fresh biopsies of HGSOC patients, following the same procedure of Sueblinvong and colleagues [27]. Briefly, biopsies were cut with a sterile razor blade and incubated with dispase II (2.4 U/ml) (Gibco - #17105–041-5 g) in DMEM at 5% CO2 and 37 °C for 30 min. After 30 min of incubation, the cell mixture was transferred onto a cell strainer (70 μm mesh). The obtained cell suspension was centrifuged, and the obtained cell pellet was resuspended, counted and plated. Primary cells were maintained in Dulbecco’s modified Eagle’s medium (DMEM) (#SD6546-500 mL; Sigma‒Aldrich) supplemented with 10% fetal bovine serum (FBS) (Gibco) and 2 mM glutamine (#G7513-100 mL; Sigma‒Aldrich).

All experiments were conducted between the fifth and sixth in vitro passages. The obtained primary cells are named the PMOV# number, where PM stands for preclinical models, OV for HGSOC, and # for the order in which the cell line was established.

### Animal studies

For the xenograft experiments, female NSG 8/10-week-old mice were purchased from Charles River Laboratories (Lecco, IT). The mice were housed in a specific pathogen-free (SPF) animal facility under a controlled temperature and light/dark cycle (12 h/12 h). Furthermore, they had unrestricted access to food and water.

SKOV3_luc clones (5 × 10^6^) were subcutaneously (s.c.) injected into the posterior flank of the mice or intraperitoneally (i.p.) injected for tumour dissemination. On day 15 or day 21, the mice received 6–10 doses of CBDCA (20 mg/kg) (#S1215 – Selleckchem) by i.p. administration every 2 days.

Kuramochi_luc cells infected with doxycycline-inducible lentiviral particles encoding short hairpin RNA targeting human Pin1 (shPin1) (5 × 10^6^) were s.c. injected into the posterior flank of the mice or i.p. injected into the mice. At the time indicated, doxycycline treatment (2 mg/ml) was started in the drinking water and D(+) sucrose (#A2211,1000 - PanReac Applichem, Darmstadt, DE) was added at a concentration of 2% every 72 h. The mice received 10 doses of CBDCA (20 mg/kg) by i.p. administration every 2 days, as indicated.

Tumour growth and dissemination were monitored at the indicated times via an IVIS Lumina III In vivo Imaging System (Caliper Life Science, Waltham, MA, USA) and IVIS living image software (Caliper Life Science) after D-Luciferin (#770504 - Perkin Elmer, Whaltam, MA, USA), as previously described [28]. Briefly, it was i.p. injected (150 mg/kg body weight) into the mice, and 10 min later, luciferase imaging was performed with IVIS. The total flux was calculated and expressed as photons per second.

No mice were excluded from the experiments. The number of mice used for each experiment is indicated in the Figure legends.

### Statistical analysis

The sample size was determined on the basis of prior experience and knowledge gained through previous similar investigations.

No data were excluded from the analysis except when a technical problem occurred in the measure.

The blinding of researchers or investigators was deemed irrelevant because of the nature of the experimental design and the specific characteristics of the interventions employed. The primary reason for not implementing blinding was the absence of subjective or observer-dependent measurements that could be influenced by knowledge of group assignments. This study focused primarily on objective and quantifiable outcomes, where measurements such as biochemical assays, physiological parameters, or histological analyses were utilized. These assessments were conducted via standardized and automated procedures, minimizing the potential for observer bias.

Descriptive statistics were employed to summarize the study information. Prior to conducting the statistical tests, the appropriateness of the data distributions’ normality and homogeneity of variances were evaluated. The normality distribution of the data was assessed via the Wilcoxon–Shapiro test. Parametric or nonparametric tests, as indicated in the figure legends, were selected on the basis of the distribution of the data.

The associations between categorical variables were tested by Fisher’s exact test. HGSOC samples from our *in-house* dataset (*n* = 62) were stratified into “High” and “Low” expression groups on the basis of the expression levels of the Notch3 and Pin1 proteins, respectively, as described in the study population section. P values were determined via Fisher’s exact test on a contingency table, with columns indicating the expression levels of Notch3 and rows indicating the expression levels of Pin1.

Multiple comparison analyses were performed via unpaired t tests (with Welsh’s correction where indicated) or one-way ANOVA followed by Tukey’s or Sidak’s post hoc tests, and statistical significance was set at *P* ≤ 0.05. The results are expressed as the means ± SDs from an appropriate number of experiments (at least three biological replicates). Significance: ns = not significant *P* > 0.05, *P ≤ 0.05, **P ≤ 0.01, ***P ≤ 0.001¸****P ≤ 0.0001.

GraphPad Prism version 8 software (GraphPad Prism, San Diego, CA, USA) and the R environment for statistical computing (v. 4.2.2) were used for all analyses.

## Results

### Notch3 overexpression correlates with platinum-resistance in ovarian cancer cells

**Fig. 1:**
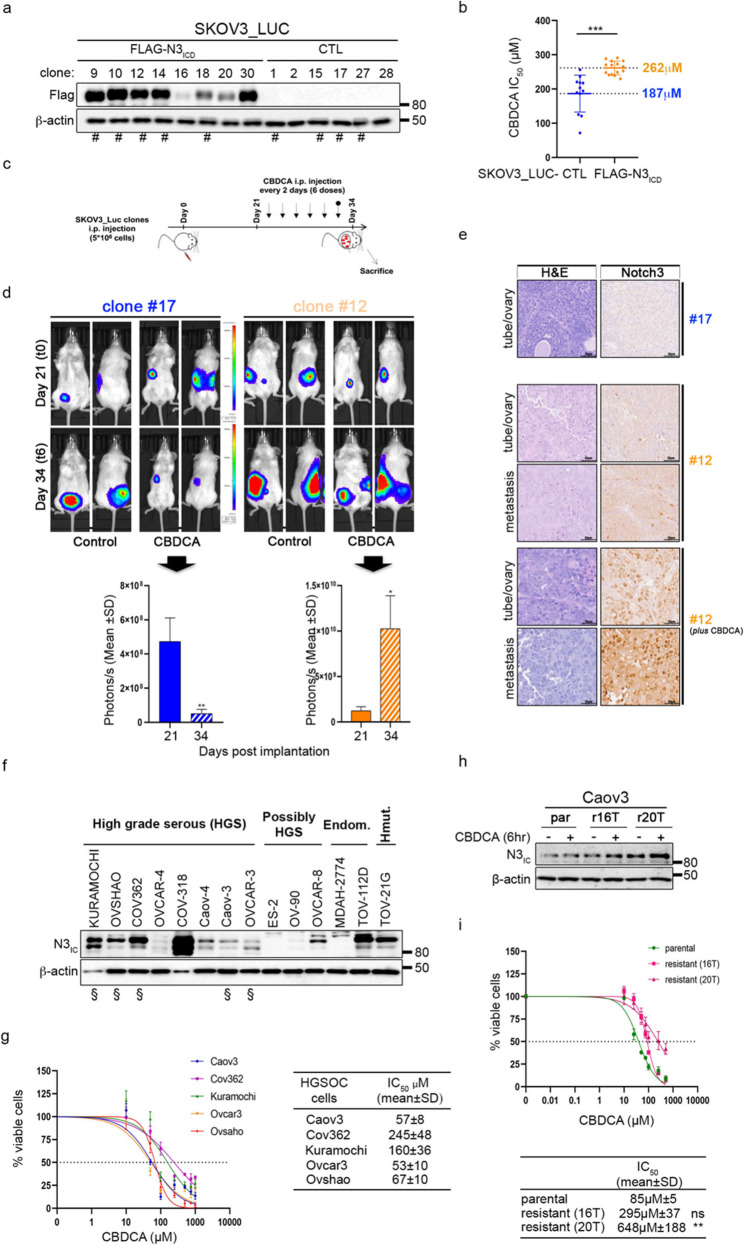
Notch3 is involved in platinum drug resistance in vitro and in vivo. **a-e**.SKOV3-LUC clones. **a** Anti-Flag antibody was used to detect FLAGN3ICD levels. **b** Scattered dot plot reporting the CBDCA IC50 of selected clones (#) subjected to increasing doses of CBDCA for 72 h and then released in drug-free medium for an additional 24 h. Each dot represents one biological replicate. The bars indicate the means ± CIs. Significant differences were computed via an unpaired t test with Welch’s correction. The average IC50 value of three independent experiments ± SD of each clone is reported. **c-e.** NSG mice (n = 4 for each group) bearing the selected clones (#) were intraperitoneally (i.p.) injected with CBDCA (20 mg/kg) every two days (treatment scheduling in **c**). Tumour growth was monitored via optical imaging at the indicated times. **d** Representative images (upper panels) and quantitative analysis (lower panels) of luciferase activity at the indicated times. Statistically significant differences in average radiance (expressed as the mean ± SD) are indicated. P values were calculated via an unpaired t test. The unpaired t test for clone #12-bearing mice was computed with Welch’s correction. **e** Representative H&E and N3 immunohistochemical staining of tubes/ovaries and metastatic nodules from the indicated SKOV3_LUC clones (#). Scale bar = 25 μm. Original magnification 20X. **f**. Western blot analysis to detect N3ICD endogenous levels in different subset of OC cells [High grade serous (HGS), possibly HGS, Endometrioid, Hypermutated]. **g-i**. HGSOC cells (**g**) CBDCA dose–response curves. HGSOC cells (§) were treated as in **b**. The results are expressed as the percentage of viable cells with respect to untreated cells (left panel), and the resulting IC50 values are reported (right panel). **h-i.** Parental (par) and isogenic-resistant (r16T and r20T) Caov3. **h** Immunoblotting analyses of parental and CBDCA-resistant Caov3 cells treated with or without a suboptimal dose of CBDCA (6 h). (**i**) CBDCA dose–response curves on parental and isogenic-resistant cells treated as in **b**. The results are expressed as the percentage of viable cells with respect to untreated cells (upper panel), and the resulting IC50 is expressed as the mean value of three independent experiments ± SD (lower panel). The difference between parental and isogenic-resistant cells is reported. Statistical significance was determined by one-way ANOVA followed by Tukey’s multiple comparisons test. ns = not significant *P* > 0.05, *P ≤ 0.05, **P ≤ 0.01, ***P ≤ 0.001¸****P ≤ 0.0001. In **a**, **f **and **h**, an anti-β-actin antibody was used as a loading control

Given the Notch3 (N3) pleiotropic effects in OC, we first assessed its involvement in platinum-resistance by using our newly generated N3-overexpressing OC clones (FLAG-N3_ICD_) (Fig. 1a): in vitro, they presented Carboplatin (CBDCA) IC_50_ values greater than those of their counterparts (CTL) (Fig. 1b and Supplementary Fig. S1a), and consistently displayed decreased DNA damage and apoptosis under chemotherapy (Supplementary Fig. S1b and S1c).

According to previous in vivo studies [29, 30] we used selected *luciferase-*expressing OC cells (SKOV3_LUC-CTL and SKOV3_LUC-FLAG-N3_ICD_) to evaluate how N3 overexpression affects OC tumour growth and dissemination in vivo. Clones were subcutaneously injected into female NOD/SCID gamma (NSG) mice. After engraftment (day 15), the mice were treated with CBDCA every two days for up to ten times (Supplementary Fig. S1d). In line with the N3-dependent proliferative advantage observed in vitro (see the vehicle panels in Supplementary Fig. S1c), N3_ICD_-expressing clones (#9 and #12) generated larger tumour masses than did negative clones (#17) before the start of treatment (t0). Moreover, tumour growth was decreased only in treated clone#17-bearing mice, whereas the presence of N3 clearly correlated with CBDCA-resistant disease (Supplementary Fig. S1e).

Since platinum-resistant OC mainly invades the peritoneum, omentum, and organs located in the abdominal cavity, we also evaluated the impact of N3 overexpression on intraperitoneal spread, which resembles the metastatic dissemination that occurs in OC-bearing patients [31]. Female NSG mice were intraperitoneally grafted with the same cells as above, and after engraftment (day 21), each group was randomized and treated with either CBDCA or vehicle (PBS) every two days for a reduced time (t6) (Fig. 1c) to avoid the N3-driven tumour burden, which progressively resulted in evident hepatic metastases 6 weeks after inoculation (Supplementary Fig. S1f), thus affecting survival. As expected, the presence of N3 promoted diffuse tumour spreading with the formation of metastatic nodules (Fig. 1d and e). CBDCA treatment significantly reduced both disease progression and tumour infiltration in the selected clone #17, whereas these effects were amplified in the clone #12-bearing mice (Fig. 1e - H&E panels), as also strongly indicated by the bioluminescence signals (Fig. 1d). Interestingly, when we evaluated mice engrafted with N3-positive clone #12, we observed a clear increase in N3 expression and consistently greater tumour infiltration in CBDCA-treated tumours than in untreated ones (Fig. 1e - Notch3 panels), confirming previous in vitro data (Fig. 1b and Supplementary Fig. S1a).

Then we moved into the HGSOC field by selecting appropriate cellular models recapitulating HGSOC features [32]. First, we tested them for N3 protein expression (Fig. 1f) and their response to CBDCA treatment (Fig. 1g): interestingly, we observed that higher levels of N3 expression are associated with higher CBDCA IC_50_ values (Fig. 1f and g).

To further confirm the correlation between N3 overexpression and increased platinum resistance in HGSOC, we established and characterized a new model of platinum-resistant HGSOC cell line, the CBDCA-resistant Caov3 cells. Specifically, we generated two isogenic platinum-resistant populations after 16 and 20 cycles of chronic drug treatment (herein referred to as r16T and r20T) by using the pulse method (Supplementary Fig. S2a). In keeping with previous findings, we showed that N3 protein expression progressively increases after platinum-based treatment, being even more evident after short CBDCA treatment and mainly in r20T population (Fig. 1h), consistently with a significant increase in its CBDCA IC_50_ (7-fold greater than that in parental cells) (Fig. 1i). Comparing to parental cells, Caov3-r20T population also displayed several features that recapitulate the clinically relevant resistant phenotype, such as (i) the acquired cross-resistance to Cisplatin (CDDP) and Taxol (Paclitaxel) (Supplementary Fig. S2b), two other chemotherapeutic drugs commonly used to treat HGSOC patients, (ii) less DNA damage, as demonstrated by the reduced phosphorylation levels of Histone H2AX (γH2AX) (Supplementary Fig. S2c-e), and increased RAD51 foci (Supplementary Fig. S2f-h), finally resulting in (iii) decreased apoptosis under chemotherapy pressure (Supplementary Fig. S2i).

Furthermore, proteomic analysis of PT-resistant Caov3 cells revealed several deregulated proteins (*n* = 316 out of 4,343 proteins measured) (Supplementary Fig. S2l) involved in multiple mechanisms associated to platinum-resistance phenotype [33], including the regulation of drug turnover, the metabolic reprogramming, the activation of epithelial-mesenchymal transition (EMT) program (Supplementary Fig. S2m).

Collectively, these findings strongly support the fundamental role of N3 in sustaining tumour growth, dissemination, and platinum resistance in ovarian cancer, thus suggesting that N3 inhibition holds promise in reverting aggressive HGSOC phenotypes through the recovery of drug sensitivity.

### Pin1 and Notch3 protein levels are correlated in HGSOC

To overcome the numerous challenges associated with Notch-targeted therapies [15] by identifying therapeutic options based on Pin1 inhibition, we first investigated the existence of potential Pin1/N3 crosstalk in HGSOC.

**Fig. 2:**
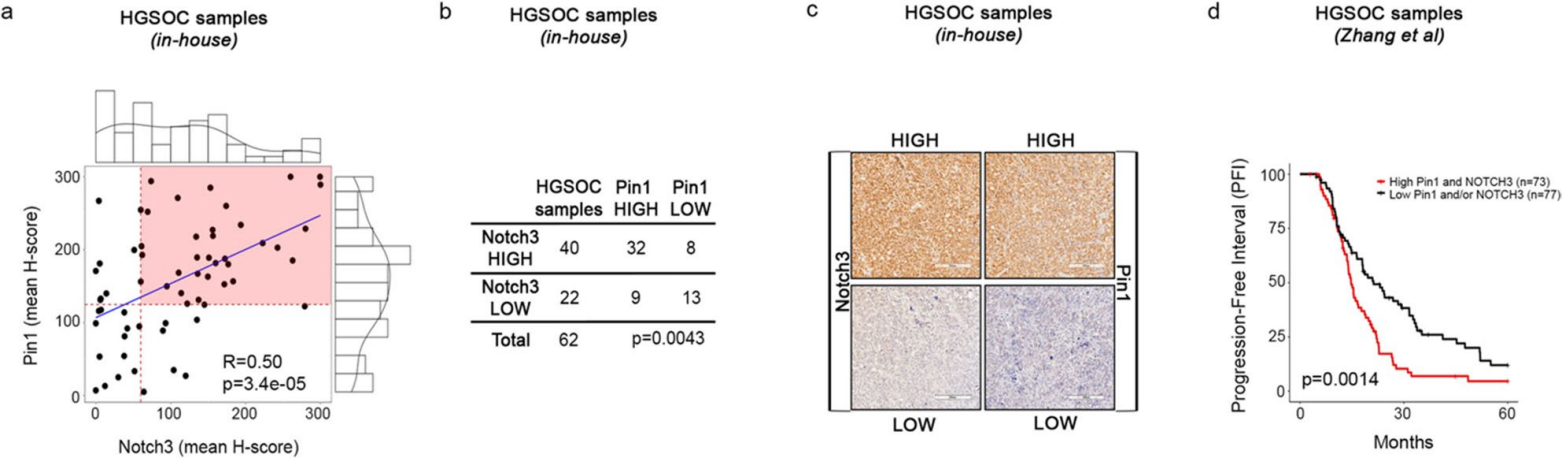
Relevance of the Pin1/Notch3 *axis* in HGSOC primary tumours. **a-c.** *In-house dataset.* **(a)** Scatterplot showing the correlations of Notch3 with Pin1 in primary HGSOC patients (*n* = 62). Values were obtained by converting immunohistochemical (IHC) staining of Notch3 and Pin1 to H scores [16]. The H score values in the plot represent the means of five independent fields for each sample. Pearson’s correlation value and p value (*P* ≤ 0.0001) are shown. The dotted red lines indicate the lower tertile used as the threshold to stratify patients with high Pin1/Notch3 expression (highlighted area). The H-score distributions for Notch3 and Pin1 are also shown in the upper and in the right panels, respectively. **(b)** Table showing the number of HGSOC samples with HIGH and LOW expression levels of Notch3 and Pin1. The stratification of HIGH and LOW expression levels was performed using the lower tertile value as the threshold. The P value is shown (*P* ≤ 0.01). Significance was computed via Fisher’s exact test. **(c)** Representative images of IHC staining of HGSOC samples expressing high levels (upper panels) or low levels (lower panels) of Notch3 and Pin1 (scale bar = 200 μm; original magnification, 20X). **(d)** External public dataset (*Zhang et al.*). Progression-free interval (PFI) curves of HGSOC patients (*n* = 150) from Zhang et al. [26] stratified into two groups, High Pin1 and Notch3 and Low Pin1 and/or Notch3, based on the expression levels of Pin/Notch3 proteins, with the lower tertile used as the threshold. Statistical significance was calculated via the log-rank test. The P value is shown (*P* ≤ 0.01)

To this purpose, we performed immunohistochemical analysis on HGSOC primary samples from patients (*n* = 62) collected in our institute (indicated as *in-house*). We documented an interesting correlation between Pin1 and N3 protein expression (Fig. 2a) with high levels of both N3 and Pin1 proteins in 32 out of 62 patient samples (nearly 52%), resulting in the main representative group of tumours compared to those expressing low levels of at least N3 and/or Pin1 (Fig. 2b, c and Supplementary Fig. S3). Interestingly, by interrogating a dataset available online (indicated as *Zhang et al.)* that comprises a larger cohort of HGSOC samples (*n* = 150), we observed that high levels of both Pin1 and N3 proteins also correlated with a shorter progression-free interval (PFI) (Fig. 2d).

These results suggested that a functional interplay between Pin1 and N3 might also occur in HGSOC, similar to what we observed in T-ALL [19].

### Pin1 and Notch3 interaction results in increased Notch3 stability under platinum pressure in HGSOC

**Fig. 3:**
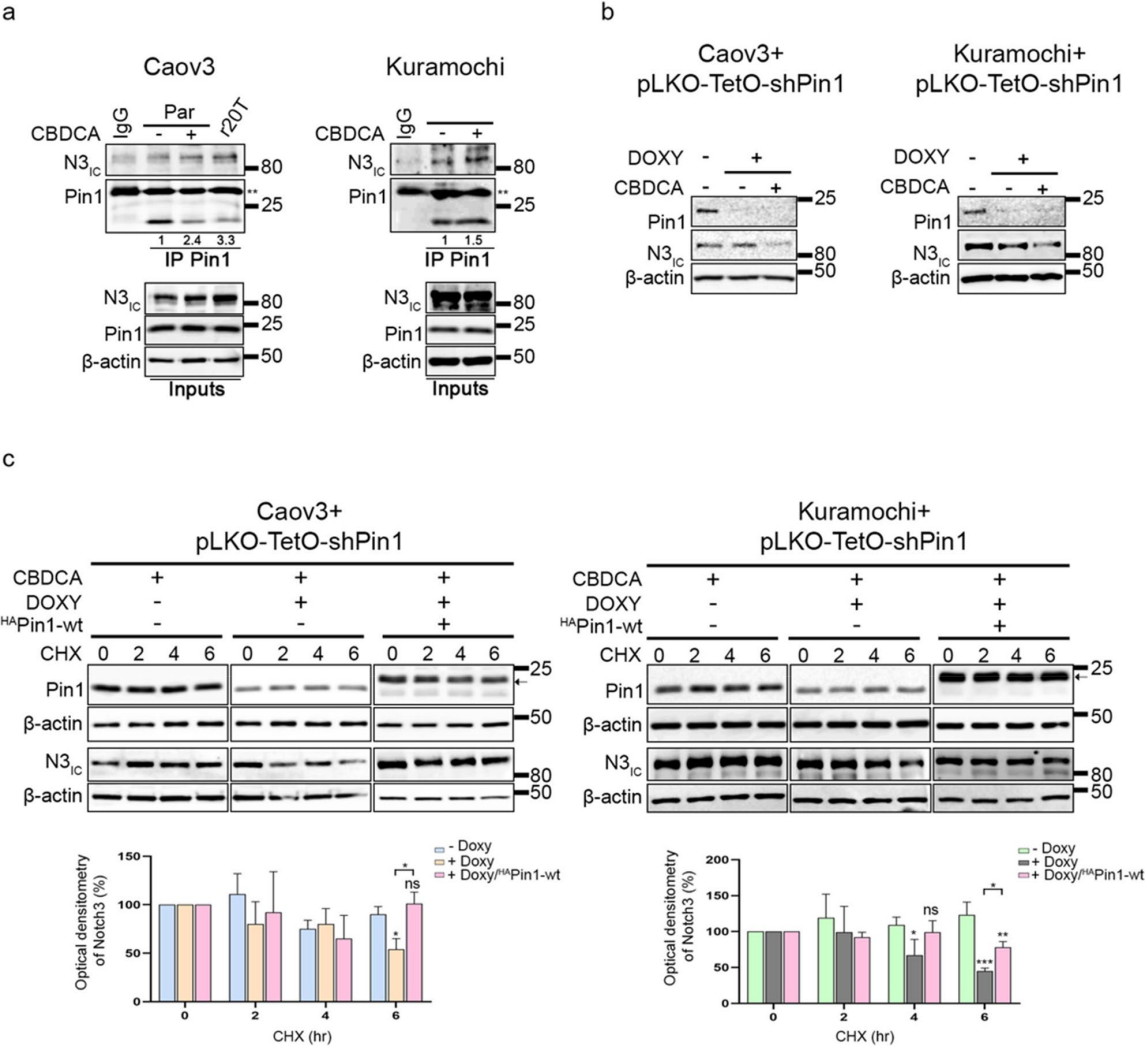
Pin1 favours Notch3 stability under platinum pressure in HGSOC. **a** Co-IP analyses of Pin1 and N3 proteins in parental vs. CBDCA-resistant Caov3 cells (r20T, left panel) and in Kuramochi cells (right panel), treated or not treated with a suboptimal dose of CBDCA (6 h and 9 h respectively). Densitometric analyses of normalized Notch3-IP/Pin1-IP expression are reported. **b** Immunoblotting analyses of N3 expression in Caov3 + pLKO-TetO-shPin1 (left panel) and Kuramochi+pLKO-TetO-shPin1 (right panel) cells upon shPin1 induction (DOXY, 48 h) with or without CBDCA treatment (6 h). **(c)** Immunoblotting analyses of N3 protein stability in Caov3 + pLKO-TetO-shPin1 (left panel) and Kuramochi+pLKO-TetO-shPin1 (right panel) cells treated with a suboptimal dose of CBDCA (6 h) upon shPin1 induction (DOXY, 48 h) combined with or without HAPin1-wt overexpression. Cells were analysed after time-course treatment with cycloheximide (CHX) for 0–2–4–6 h (upper panels). Densitometric analyses of β-actin-normalized N3 protein levels are shown as the mean value of three independent experiments ± SD (lower panels). The results are expressed as percentages with respect to time 0. The difference between the control group (–DOXY) and each sample and, where significant, among the samples is reported. Statistical significance was computed for each time point via one-way ANOVA followed by Tukey’s multiple comparisons test. ns = not significant *P* > 0.05, *P ≤ 0.05, **P ≤ 0.01, ***P ≤ 0.001¸****P ≤ 0.0001. Anti-β-actin antibody was used as a loading control. In **a**, ** indicates light chains. In **c**, arrows indicate exogenous HAPin1-wt.

In order to deepen the Pin1/N3 relationship in HGSOC, we first generated and tested HGSOC cells (KURAMOCHI and Caov3 selected from Fig. 1) expressing a doxycycline-inducible Pin1-knockdown construct (pLKO-TetO-shPin1) both in vitro and in vivo (Supplementary Fig. S4a, c-f). Since Pin1-depleted (DOXY) cells retained endogenous N3 expression (Supplementary Fig. S4a), we hypothesized that Pin1 was able to positively regulate N3 under platinum pressure. Indeed, coimmunoprecipitation experiments demonstrated that these proteins interacted at baseline, but short CBDCA-treatment increased their binding (Fig. 3a). Notably, the Pin1‒N3 interaction in CBDCA-treated Caov3 cells reached the same level as that observed under basal conditions in CBDCA-resistant Caov3 cells (r20T in Fig. 3a, left panels).

Consistently, Pin1 silencing (+ DOXY) resulted in a significant decrease in N3 expression under CBDCA treatment (Fig. 3b), which was also correlated with a reduced N3 half-life (Fig. 3c and Supplementary Fig. S4b). Accordingly, transient Pin1 overexpression reversed this effect (Fig. 3c), indicating that N3 protein levels are Pin1-dependent.

Overall, these observations suggest that the Pin1/N3 *axis* might be involved in the acquisition of an aggressive phenotype in HGSOC, as increased N3 protein stability may result in platinum resistance [12] and consequently in tumour recurrence [4].

### Pin1 binds to Notch3 through specific residues in its intracellular domain

**Fig. 4:**
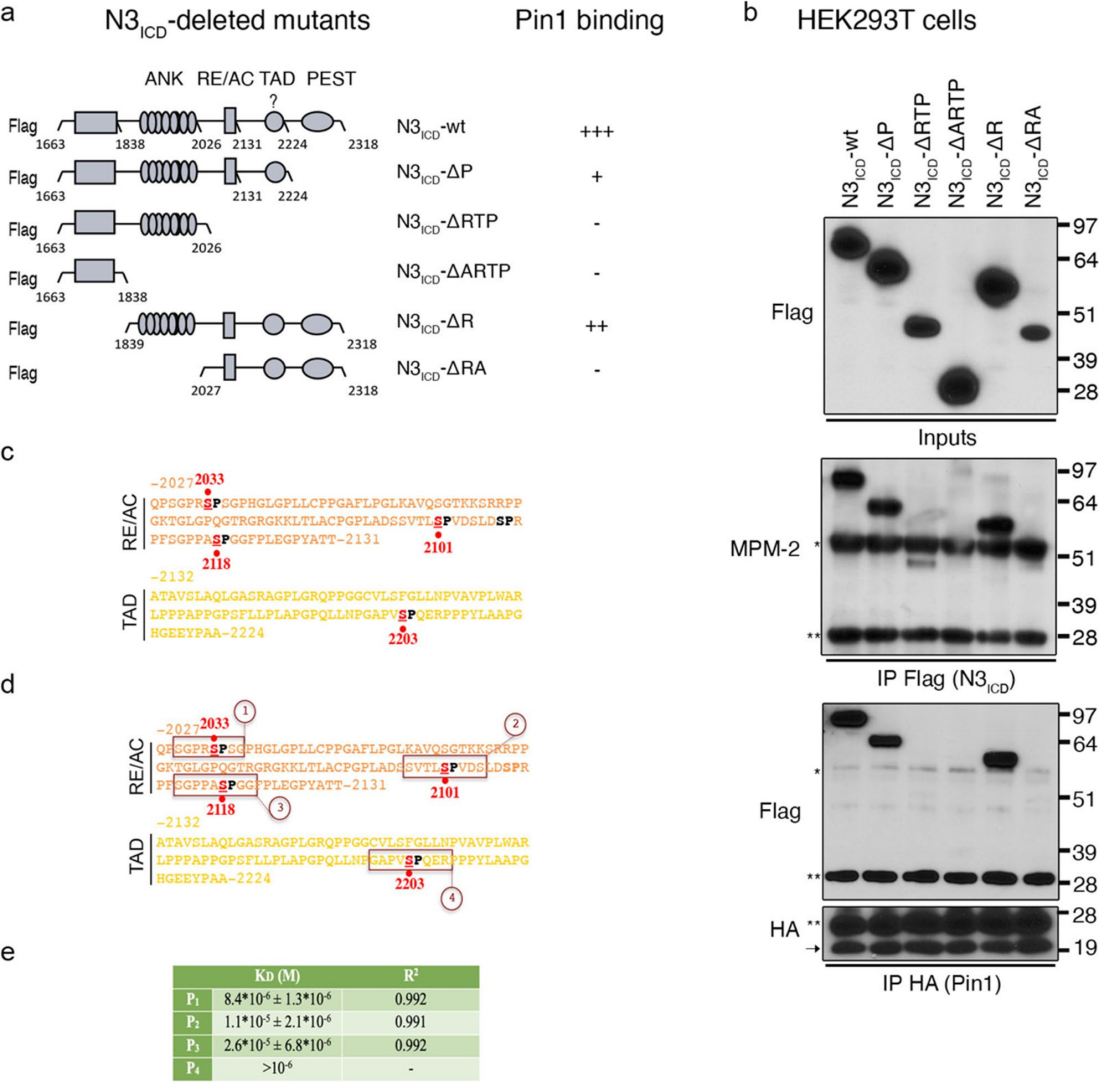
Pin1 binds to specific residues of Notch3_ICD_. **a** Illustration of ^FLAG^N3_ICD_-wt and -deleted mutants. Interactions with Pin1 are indicated next to the constructs (+++: strong; ++: intermediate; +: weak; –: no binding). **b** Immunoblotting of ^FLAG^N3_ICD_-wt and -deleted mutants (upper panel), analysis of their phosphorylation *status* (middle panel) and co-IP analyses of ^HA^Pin1/^FLAG^N3_ICD_ binding in HEK293T cells (lower panel). Anti-MPM-2 was used to detect phospho-S/T-P motifs. * and ** indicate heavy and light chains, respectively, whereas arrows indicate exogenous ^HA^Pin1. **c**,** d.** Illustration of the RE/AC and TAD regions of N3_ICD_ showing the four identified phosphorylated Pin1 consensus motifs (red and bold). **d** The designed four peptides containing Pin1 consensus sites. In **a**,** c** and **d**, ANK: ankyrin; RE/AC: repression/activation domain; TAD: transcriptional activation domain. The numbering refers to UniProtKB entry Q61982. **e** List of KD and related R^2^ values for the interaction of the PIN1 protein with N3-derived peptides

From a mechanistic perspective, we first investigated how Pin1 sustains N3. We previously demonstrated that the N3 intracellular domain (N3_ICD_) harbours several Pin1 consensus motifs (serine/threonine residues preceding a proline, Ser/Thr-Pro) [34] and that Pin1 is able to directly interact with N3_ICD_ [19]. To identify the residues involved in this interaction, we generated various Flag-tagged N3_ICD_ deletion mutants (Fig. 4a, and 4b upper panel). We subsequently assessed their phosphorylation *status* at specific Pin1 motifs as well as their interaction with HA-tagged Pin1, as indicated by western blot analysis with phospho-specific MPM-2 and anti-Flag antibodies (Fig. 4b, middle and lower panels). Furthermore, we mapped these residues within the ANK, RE/AC, and TAD domains of N3_ICD,_ which collectively harbour seven Ser/Thr-Pro motifs (Supplementary Fig. S5a). Among these motifs, four (S2033, S2101, S2118, and S2203) were identified as phosphorylated (Fig. 4c), which is consistent with our previous findings [35], and were found to be evolutionarily conserved across species (Supplementary Fig. S5b).

A series of peptides mimicking the amino acid sequences identified as Pin1 substrates were synthesized, hereafter named P_1_--_4_ (Fig. 4d and Supplementary Fig. S5c). The interaction between N3-related peptides and Pin1 was examined via biolayer interferometry technology [36]. To this purpose, the four amino acid sequences were functionalized with a biotin moiety at the N-terminal end (Supplementary Fig. S5c), allowing their binding to streptavidin and immobilization on high-streptavidin (SSA) optical sensors through molecular recognition. Dose‒response binding analyses across six analyte concentrations revealed that Pin1 binds to all the tested peptides in a dose-dependent manner, confirming its direct interaction with Pin1 (Supplementary Fig. S5d). However, steady-state analysis revealed that the most pronounced dose-dependent binding to Pin1 was observed for the P_1_ peptide, which exhibited an apparent binding affinity (KD) in the low micromolar range (Fig. 4e and Supplementary Fig. S5d). As further evidence of this finding, pull-down experiments coupled with LC‒MS analysis demonstrated that, among all the tested peptides, the P_1_ peptide consistently coeluted with Pin1 when incubated at a 1:1 stoichiometric ratio (Supplementary Fig. S6-S9).

**Fig. 5:**
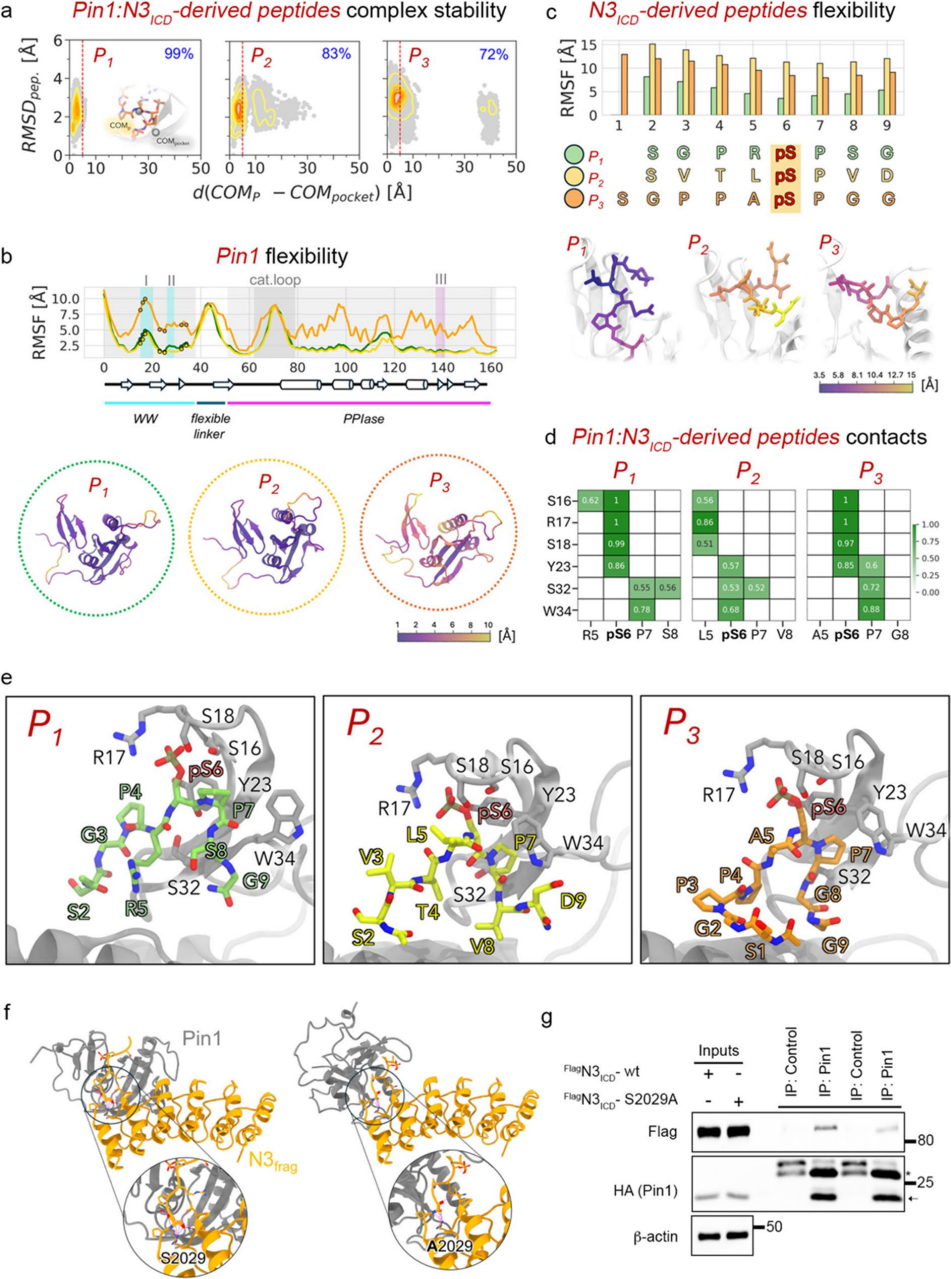
Structural and dynamic determinants of Pin1-Notch3 interactions. **(a)** Bidimensional landscape as a function of two order parameters: RMSD_peptide_ (Å) and distance between the centre of mass (dCOM) (Å) of the peptides and the WW domain binding region. **(b)** Root mean square fluctuation (RMSF) (Å) of Pin1 Cα atoms. The peptide binding residues are represented as filled circles corresponding to the colours in **S10d** (upper panel). Pin1 coloured according to the RMSF averaged over five replicas for each peptide (indicated in the dashed circles, corresponding to the colours in S10d). The colour range is from 1 Å, dark purple, to 10 Å, yellow, as indicated by the colour bar (lower panel). **(c)** RMSF (Å) of peptide backbone atoms (upper panel). P_1_, P_2_ and P_3_ are coloured according to the RMSF averaged over five replicas for each one. The colour range is from 3.5 Å, dark purple, to 15 Å, yellow, as indicated by the colour bar (lower panel). **(d)** Interaction fingerprint of Pin1-peptide complexes. Frequencies are calculated for all interaction types and filtered to include only interactions that are found to have a frequency over 50% in at least one condition. The interaction frequency is represented via a scale ranging from white (0%) to green (100%). **(e)** Inter residue interactions between the Pin1 WW domain and peptides corresponding to **d**, visualized over representative Pin1-peptide complexes extracted from MD simulations. **(f)** Structures of the Pin1–N3_− fragment_ complexes in the wild-type (S2029) and mutant (S2029A) forms, generated using Boltz2. The inset shows a close-up view of the wild-type and the mutated amino acid and neighbouring interacting residues. Hydrogen bonds are depicted as black dashed lines, while van der Waals (vdW) interactions are shown in magenta. **(g)** Co-IP analyses of ^HA^Pin1/^FLAG^N3_ICD−wt_ and ^FLAG^N3_ICD−S2029A_. Binding in HEK293T cells. * indicates light chains, while the black arrow indicates exogenous ^HA^Pin1.

Rosetta FlexPepDock ab initio docking followed by multiple independent atomistic molecular dynamics (MD) simulations was employed to model peptide–Pin1 interactions. Peptide stability, assessed by *peptide RMSD* (Supplementary Fig. S10 a, b and d) indicated that P_1_ formed the most stable complex (2.22 ± 0.48 Å) compared with P_2_ (2.34 ± 0.55 Å) and P_3_ (2.92 ± 0.47 Å). Consistently, centre-of-mass distance (dCOM) and RMSD analyses (Fig. 5a; Supplementary Fig. S10c) showed that P_1_ remained bound for 99% of the simulation time, whereas P_2_ and P_3_ underwent earlier unbinding events. The bidimensional dCOM–RMSD landscape of P_2_ displayed a broader distribution of partially unbound states (10–20 Å), while P_3_ populated a single, well-defined unbound state at ~ 40 Å from the WW domain (Fig. 5a). These computational trends qualitatively mirrored the experimentally determined dissociation constants (KD) (Fig. 4e; Supplementary Fig. S5d).

To further characterize peptide-dependent conformational effects, essential dynamics analysis was performed via principal component analysis (PCA) on peptide backbone conformations pooled from five independent simulations per complex (Supplementary Fig. S11a, b) [30]. RMSF profiles of Pin1 (Fig. 5b) and the peptides (Fig. 5c) revealed pronounced rigidity of the P_1_ core region encompassing the Pin1 consensus motif (pSer6/Pro7), whereas corresponding regions in P_2_ and P_3_ remained more flexible. Persistent contact analysis showed that P_1_ uniquely established stable interactions with key Pin1 residues (Ser16, Arg17, Tyr23, Ser32, Trp34), driven by its charged Arg5 and polar Ser8 (Fig. 5d, e). Consistent with these findings, mutation of these residues in P1 (P1m; Supplementary Fig. S5c) markedly impaired binding in both BLI (Supplementary Fig. S12a) and pull-down assays (Supplementary Fig. S12b–e). Furthermore, competition experiments demonstrated that the wild-type P1 peptide competed more effectively with the full-length protein for Pin1 binding than did P1m (Supplementary Fig. S12f).

Notably, we observed that in the isolated P1 peptide, S2029 is extensively solvent-exposed and highly flexible, forming only transient hydrogen bonds with N30 and P149 of Pin1 (occupancy of 26.4% and 21.1%, respectively), located at the interface between WW and PPIase domains. However, in the full-length N3 protein, the upstream ANK repeat domain imposes structural constraints that likely stabilize polar interactions involving S2029. This restriction of conformational freedom may limit compensatory rearrangements, thereby potentiating the structural impact of S2029 on the Pin1–N3 interaction. To test this hypothesis, we employed Boltz2 [37] to generate two putative interaction models comparing the wild-type Pin1–N3_frag_ (residues 1837–2040, encompassing the ANK domain and the P1 region) with the S2029A mutant (Fig. 5f). Intermolecular contact analysis confirmed that the S2029A substitution results in the loss of a critical hydrogen bond and multiple van der Waals interactions (Fig. 5f). Consistently, direct binding assays between the P1-S2029A peptide and Pin1 showed that the mutant peptide retains an affinity similar to the wild-type P1 (Data not shown). This can be explained by the marked flexibility of the P1 peptide extracted from N3, which may allow the formation of compensatory hydrophobic contacts involving A2029 (e.g., hypothetically with L86 and L156). The functional importance of S2029 in the context of the full-length proteins was further validated by Co-IP experiments (Fig. 5g), which demonstrate that the S2029A mutation nearly abrogates the interaction between N3 and Pin1.

Collectively, these data demonstrate that Pin1 recognizes and binds the P1 peptide with higher efficiency compared to other tested sequences. This suggests a preferential role for P1 in the Pin1–N3(ICD) interaction, primarily mediated by pS3033 and the presence of a positively charged arginine residue preceding the phosphorylated serine, a unique feature of this sequence. Furthermore, the S2029 residue serves as a significant structural determinant of the Pin1–N3 association.

### Pin1 regulates Notch3_ICD_ stability by competing with the kinase GSK3β

To better characterize the molecular mechanism whereby Pin1 positively regulates N3_ICD_, we wondered whether stabilization resulted from masking overlapping sites of N3 negative regulators. Therefore, we queried the ELM database [38] for consensus motifs that overlap with Pin1 binding sites, and we identified the kinase GSK3β.

**Fig. 6:**
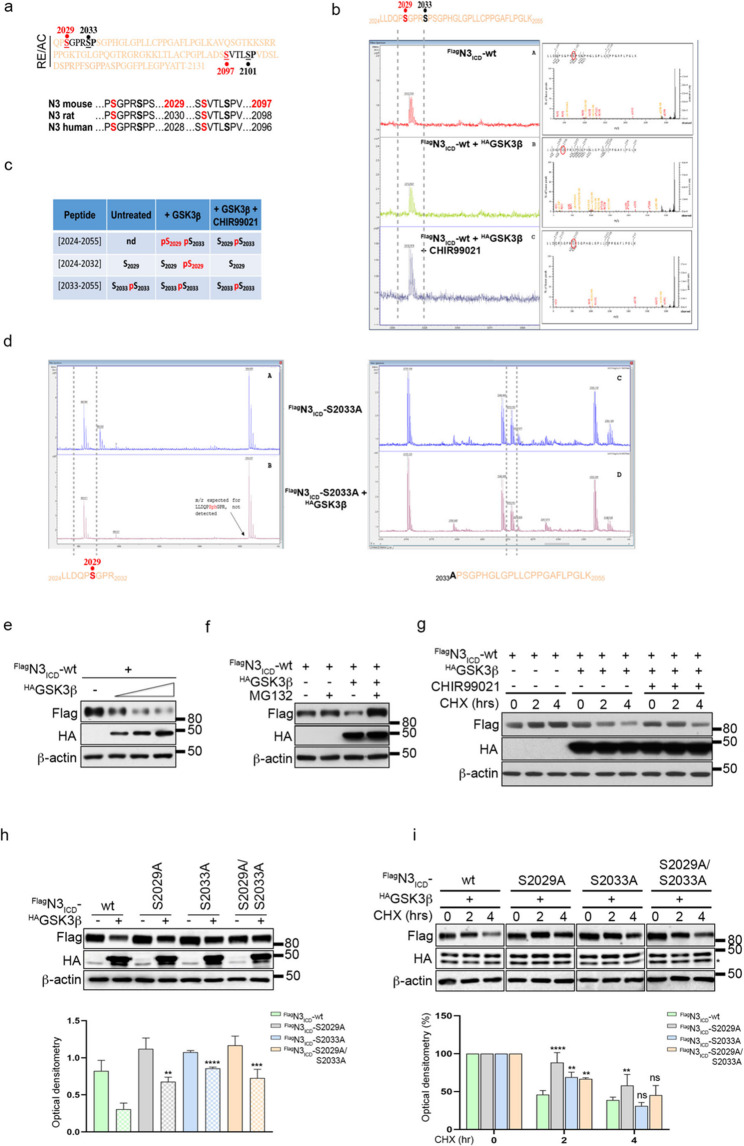
GSK3β phosphorylates Notch3_ICD_ and induces its proteasomal degradation. **a** Illustration of the RE/AC domain of N3ICD showing GSK3β consensus motifs (bold: primed residues; red: putative residues) (upper panel) and their sequence alignment across species (lower panel). **b** MALDI ToF spectra for peptide mass fingerprinting of FLAGN3ICD-wt in the absence (A) or presence of HAGSK3β, alone (B) or with CHIR99021 (2 h) (C), zoomed into the area approximately 3312 m/z corresponding to the 2024LLDQPSGPRSPSGPHGLGPLLCPPGAFLPGLK2055 peptide. Each box contains the MS/MS spectrum of the 3312 m/z signal and the identified phosphorylated residue via Biotools software. **c** Table showing the different tryptic peptides with the corresponding identified phosphorylated residues (red) detected by an Orbitrap Fusion Tribrid mass spectrometer. nd = not detected. **d** MALDI ToF spectra for peptide mass fingerprinting of FLAGN3ICD-S2033A alone (A, C) or with HAGSK3β (B, D), zoomed into the area approximately 982 m/z corresponding to the 2024LLDQPSGPR2032 peptide (A, B) or approximately 2253 m/z corresponding to 2033APSGPHGLGPLLCPPGAFLPGLK2055 peptide (C, D). In **a-d**, numbering refers to UniProtKB entry Q61982. **e-i.** HEK293T cells.** e** Immunoblotting analyses of FLAGN3ICD-wt in the presence of increasing amounts of HAGSK3β. **f**, **g**.Immunoblotting analyses of FLAGN3ICD-wt in the presence of the highest dose of HAGSK3β plasmid followed by MG132 (4 h) (**f**) or CHIR99021 (2 h) (**g**). In **g**, the cells were analysed over a time course with cycloheximide (CHX) (0–2–4 h). **h**, **i**. Immunoblotting analyses of FLAGN3ICD-wt and non phosphorylable mutants in the presence or absence of HAGSK3β (**h**) or after a time course with CHX (0-2-4 h) following the overexpression of HAGSK3β **(i)** (upper panels). Densitometric analyses of β-actin-normalized N3 levels are shown as the mean value of three independent experiments ± SD (lower panels). In **i**, the results are expressed as percentages with respect to time 0. The difference between FLAGN3ICD-wt and each non phosphorylatable mutant in the presence of HAGSK3β (**h**) or at each time point (**i**) is shown. Statistical significance was computed via one-way ANOVA followed by Sidak’s multiple comparisons test. ns = not significant *P* > 0.05, *P ≤ 0.05, **P ≤ 0.01, ***P ≤ 0.001¸****P ≤ 0.0001. Anti-β-actin antibody was used as a loading control. In **i**, * indicates a-specific bands

Most of the GSK3β substrates require a priming phosphorylated residue (serine or threonine) at amino acid + 4 (C-terminal) to be phosphorylated by GSK3β at specific serine/threonine residues in the N-terminal region, S/TXXXpS/pT (S = Ser; T = Thr; X = variable amino acid) [39]. As shown in Fig. 6a, two putative GSK3β consensus motifs, which are evolutionarily conserved across species, overlapped with Pin1 interaction motifs identified in the RE/AC domain.

Therefore, we first sought to verify whether GSK3β could act on the RE/AC of N3_ICD_. For this purpose, we transiently overexpressed N3_ICD_ and GSK3β plasmids in the presence or absence of the GSK3β inhibitor CHIR99021. After immunoprecipitation, N3_ICD_ was in-gel digested, and the trypsin digestion product was analysed via MALDI ToF. The results revealed GSK3β-dependent phosphorylation of the S2029 residue (Fig. 6b), which was further confirmed by LC‒MS/MS (Fig. 6c). Indeed, S2029 phosphorylation (pS2029) was detected in two peptides, (2024–2032) and (2024–2055), only after the overexpression of GSK3β kinase and in the absence of the CHIR99021 inhibitor (Fig. 6c and Supplementary Fig. S13). On the other hand, the S2033 residue was detected in the phosphorylated state (pS2033) regardless of the presence of the kinase GSK3β or the CHIR99021 inhibitor (Fig. 6c). Notably, the peptide (2024–2055), containing both S2029 and S2033 residues, shows phosphorylated S2029 concurrently with phosphorylated S2033, suggesting that S2033 prephosphorylation could be necessary for GSK3β recognition (Fig. 6c and Supplementary Fig. S13c and S13d). To test this hypothesis, we repeated the MS analysis by using a newly generated N3_ICD_ phospho-mutant (N3_ICD_-S2033A): as shown in Fig. 6d, the absence of a functional S2033 residue led to impaired S2029 phosphorylation, confirming the role of the phospho-site S2033 as the priming residue required to correctly drive GSK3β phosphorylation activity at the S2029 site (Fig. 6d).

By exploring the role of GSK3β in the regulation of N3_ICD_, we revealed that dose-dependent overexpression of GSK3β led to a reduction in N3_ICD_ protein levels (Fig. 6e), which was reversed by the proteasome inhibitor MG132, thus suggesting that GSK3β is involved in N3_ICD_ proteasomal degradation (Fig. 6f). Consistently, the N3_ICD_ half-life was shorter following GSK3β overexpression, which was reversed by the CHIR99021 inhibitor (Fig. 6g). In contrast, GSK3β was unable to induce a similar strong reduction in N3_ICD_ non-phosphorylable mutants (N3_ICD_-S2029A, N3_ICD_-S2033A and N3_ICD_-S2029A/S2033A), which affected GSK3β activity (Fig. 6h), resulting in their increased stability (Fig. 6i) and unresponsiveness to proteasomal degradation GSK3β-dependent (Supplementary Fig. S14).

Collectively, these findings revealed that the kinase GSK3β negatively regulates N3_ICD_ protein levels by recognizing the pS2033 residue and acting on the S2029 residue.

Given that we identified the Pin1 target phospho-site S2033 (containing in peptide P1) as the priming residue for GSK3β activity on N3_ICD_, we hypothesized that the Pin1 and GSK3β proteins may compete for N3_ICD_ binding with different functional outcomes.

**Fig. 7:**
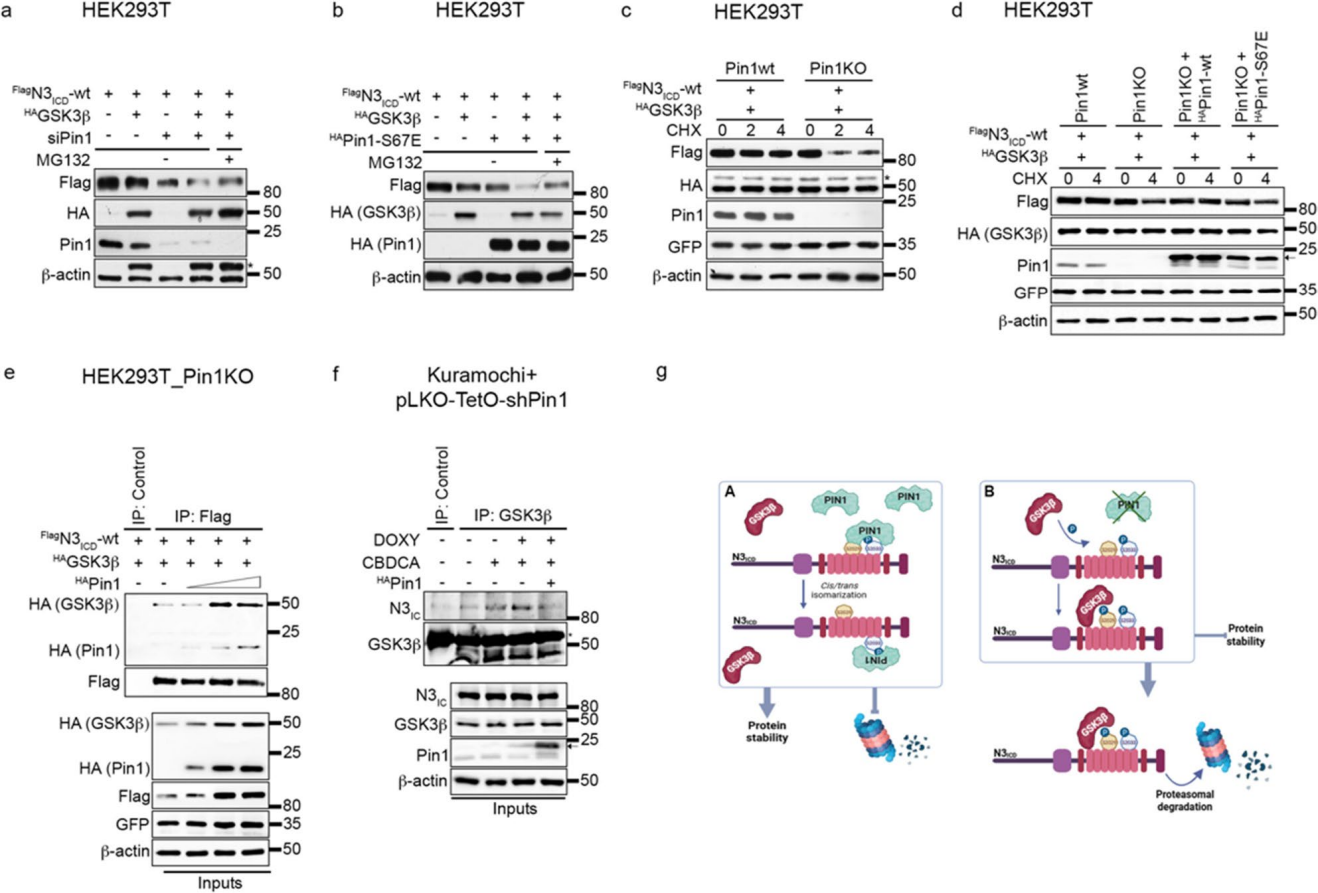
Antagonistic effects of Pin1 and GSK3β on Notch3_ICD_. **a**,** b**. Immunoblotting showing analyses of FLAGN3ICD-wt protein expression levels in HEK293T cells transiently transfected for 48 h with HAGSK3β alone or in combination with Pin1-specific siRNA (siPIN1) (**a**) or HAPin1-S67E (**b**). The cells were also treated with MG132 (4 h) as indicated. **c** Immunoblotting analyses of the FLAGN3ICD-wt half-life in Pin1 wild-type (Pin1wt) and knockout (Pin1KO) HEK293T cells transiently transfected with HAGSK3β. The cells were analysed after time-course treatment with cycloheximide (CHX) for 0–2–4 h. **d** Immunoblotting analyses of the FLAGN3ICD-wt half-life in Pin1 wild-type (Pin1wt) and knockout (Pin1KO) HEK293T cells transiently transfected with HAGSK3β. HEK293T Pin1KO cells were transfected with or without HAPin1-wt or -S67E. The cells were analysed after time-course treatment with cycloheximide (CHX) for 0–4 h. **e** Co-IP analyses of FLAGN3ICD-wt and HAGSK3β proteins in HEK293T Pin1KO cells in the presence of increasing amounts of HAPin1-wt.** f** Co-IP analyses of GSK3β and N3 proteins in Kuramochi+pLKO-TetO-shPin1 cells treated with a suboptimal dose of CBDCA (9 h) upon shPin1 induction (DOXY, 48 h) with or without HAPin1-wt under MG132 treatment (4 h). **g** A schematic model (created in BioRender) showing the antagonistic effect of Pin1 and GSK3β proteins on N3ICD. Pin1 isomerase activity on N3ICD masks the GSK3β binding site (S2033 residue), ultimately resulting in N3ICD stabilization (A). In the absence of Pin1, GSK3β recognizes the S2033 residue and phosphorylates the S2029 residue, thereby promoting proteasomal degradation (B). Anti-β-actin antibody was used as a loading control. In **a **and **c**, * indicates a-specific bands. In **c**,** d**, and **e**, an anti-GFP antibody was used to evaluate the transfection efficiency. In **d**,** f**, arrows indicate exogenous HAPin1. In **f**, * indicates the heavy chain

We documented that the activity of GSK3β on N3_ICD_ is favoured in the absence of Pin1 isomerase, which is obtained both by Pin1 silencing (Fig. 7a) or by overexpressing the catalytically inactive mutant Pin1-S67E, which is able to interact with its substrates without inducing *cis/trans* isomerization [21] (Fig. 7b). In agreement with these data, the stability of N3_ICD_ was significantly lower in the presence of GSK3β in a Pin1-deficient context (Fig. 7c), which was subsequently restored by the exogenous expression of wild-type Pin1 but not Pin1-S67E (Fig. 7d). These data indicate that Pin1 isomerase activity plays a key role in the regulation of N3_ICD_ stability.

Furthermore, Pin1 negatively affected the interaction between exogenous GSK3β and N3_ICD_ (Fig. 7e), favouring its own binding to N3_ICD_ (Fig. 7e, see blot HA (Pin1)). Accordingly, transient Pin1 overexpression in Pin1-depleted cells decreased the endogenous binding between GSK3β and N3_ICD_ under CBDCA treatment (Fig. 7f).

Overall, these findings suggest that Pin1 isomerization, through masking shared residues, protects N3_ICD_ from GSK3β-dependent proteasomal degradation by displacing their interaction (Fig. 7g).

### Suppression of Pin1 sensitizes HGSOC to platinum-based chemotherapy via Notch3 downregulation both in vitro and in vivo

**Fig. 8:**
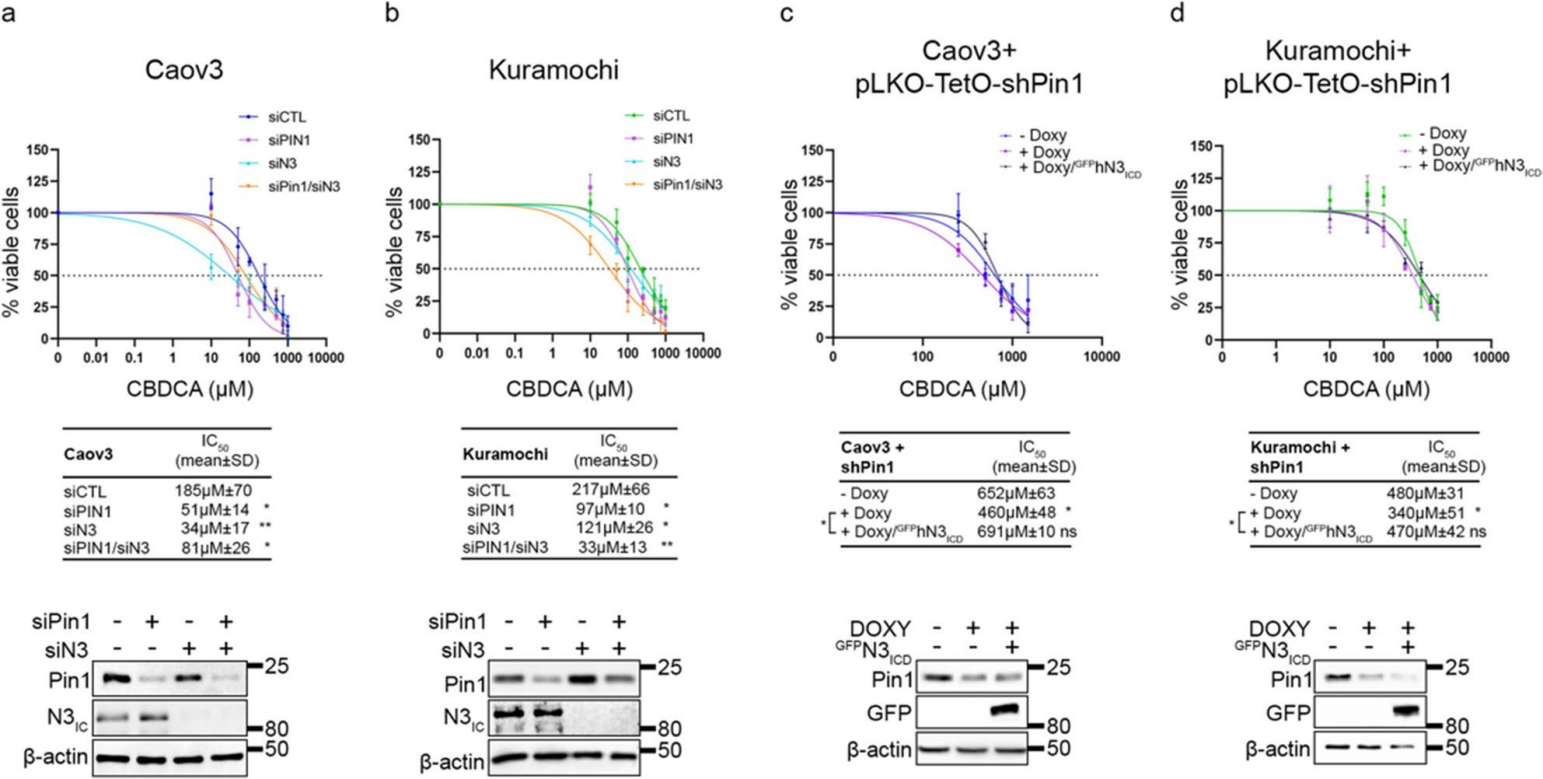
Genetic Pin1 targeting sensitizes tumour growth to platinum treatment in vitro. **a**, **b**. CBDCA dose–response curves. Caov3 (**a**) and Kuramochi (**b**) cells were transfected with control, Pin1, or N3-specific siRNAs, alone or in combination for 72 h. **c**, **d**. Caov3 + pLKO-TetO-shPin1 (**c**)and Kuramochi+pLKO-TetO-shPin1 (**d**) cells upon shPin1 induction (DOXY, 48 h) with or without overexpression of GFPN3ICD. Cells were subjected to increasing doses of CBDCA for 16 h and then released in drug-free medium for an additional 24 h. The results are expressed as the percentage of viable cells with respect to untreated cells, and the resulting IC50 is expressed as the mean value of three independent experiments ± SD. The expression of Pin1 with N3 (**a**, **b**) and GFP (**c**, **d**) is reported in the lower inset as an experimental control for transient transfections without CBDCA treatment. The difference between the control group (siCTL in **a**, **b** and –DOXY in **c**, **d**) and each sample is reported, and where significant, the difference among samples is shown. Statistical significance was determined by one-way ANOVA followed by Tukey’s multiple comparisons test. ns = not significant P > 0.05, *P ≤ 0.05, **P ≤ 0.01, ***P ≤ 0.001¸****P ≤ 0.0001. Anti-β-actin antibody was used as a loading control

The role of Pin1 in sustaining N3 protein expression prompted us to investigate whether Pin1 inhibition can lead to chemo sensitization. Accordingly, Pin1 knockdown was correlated with a significant decrease in cell survival (Fig. 8a-d). N3 silencing did not further sensitize Pin1-silenced cells to CBDCA (Fig. 8a and b), and N3 transient overexpression rescued the increased CBDCA-induced cell death in Pin1-silenced cells (Fig. 8c and d), confirming that N3 acted downstream of Pin1 in response to CBDCA.

**Fig. 9:**
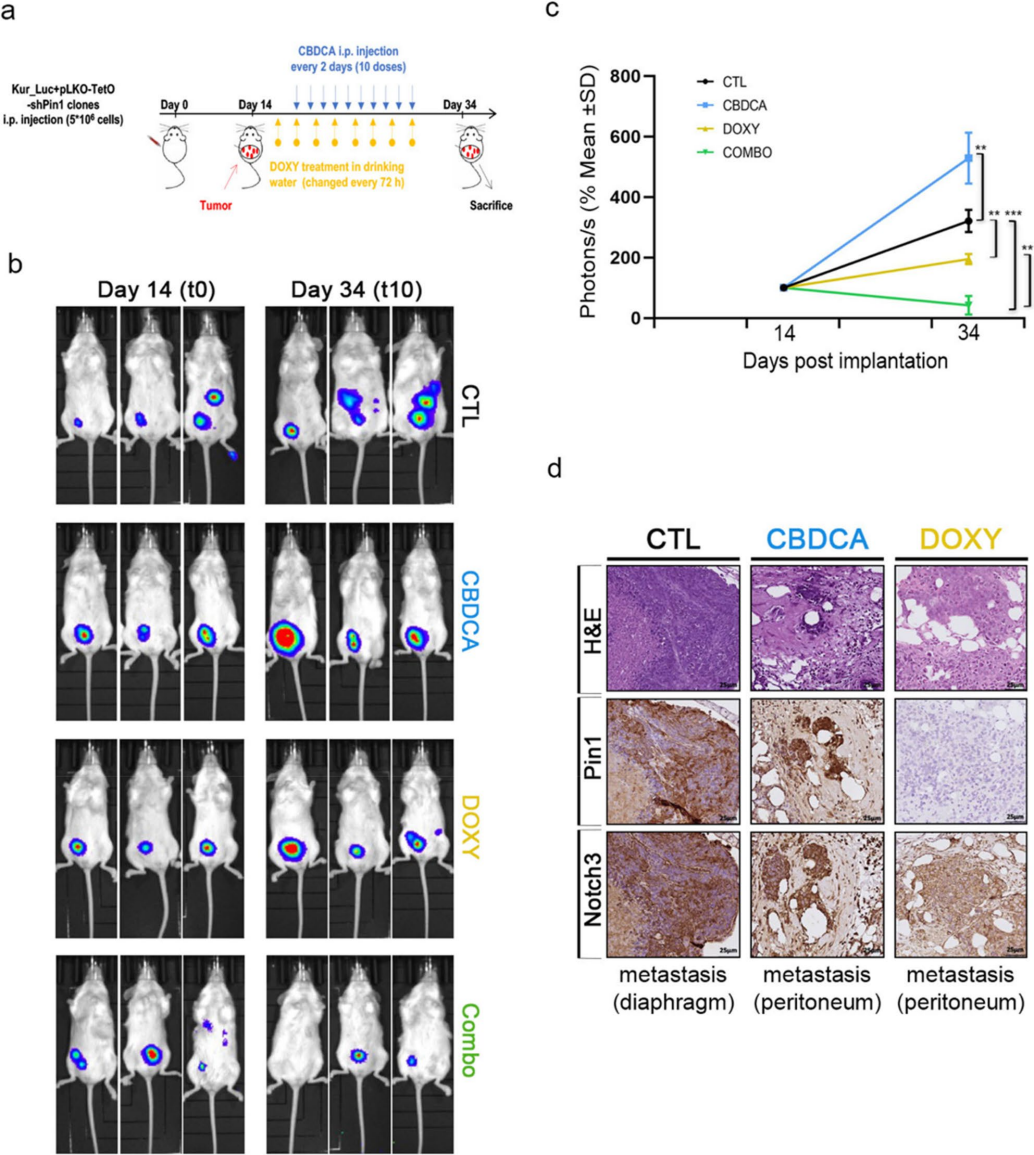
Genetic Pin1 targeting sensitizes tumour growth to platinum treatment in vivo. **a-d**.NSG mice (n = 4 for each group) were intraperitoneally (i.p.) injected with Kuramochi_Luc+pLKO-TetO-shPin1 cells and exposed to doxycycline in the drinking water every 72 h. After one administration (24 h later), the mice were treated (i.p.) with either CBDCA (20 mg/kg) or vehicle (PBS) every two days for a total of ten times (treatment scheduling in **a**). Tumour growth was monitored via optical imaging at the indicated times. **b** Representative images of luciferase activity at the indicated times. **c** Quantitative analysis of luciferase activity at the indicated times. The results are expressed as percentages with respect to time 0. Statistically significant differences at time point 10 (expressed as the mean ± SD) are indicated. P values were calculated via one-way ANOVA followed by Tukey’s multiple comparisons test. **d** Representative Hematoxylin Eosin (H&E) and Pin1 and N3 immunohistochemical staining of metastatic nodules of the indicated groups (CTL, CBDCA, and DOXY). Scale bar = 25 μm - Original magnification 20X. ns = not significant *P* > 0.05, *P ≤ 0.05, **P ≤ 0.01, ***P ≤ 0.001¸****P ≤ 0.0001

To evaluate the in vivo impact of these findings, pLKO-TetO-shPin1-LUC Kuramochi cells were tested in xenograft experiments with female NSG mice. After cell inoculation (day 15), each group was randomized and treated with either doxycycline and/or CBDCA or vehicle (PBS), and tumour growth was monitored for two more weeks with 10 doses of CBDCA (t10) (Fig. 9a). While Pin1 knockdown (DOXY) moderately delayed tumour burden, tumours treated with CBDCA monotherapy continuously progressed even more than those in the control group (CTL) did (Fig. 9b and c), as expected by our previous in vitro data showing increased Pin1/N3 interaction in CBDCA-treated cells (Fig. 3a), thereby sustaining N3 expression and CBDCA resistance. Interestingly, only the combined treatment (Combo), represented by Pin1 depletion *plus* CBDCA, effectively reduced intraperitoneal tumour dissemination, ultimately resulting in significant CBDCA sensitization (Fig. 9b and c). Notably, all the tumoral masses identified (mainly in CTL and CBDCA groups) co-expressed both Pin1 and N3 proteins, confirming the importance of a functional Pin1/N3 *axis* in the acquisition of an aggressive phenotype (Fig. 9d, left and middle panels). As expected by our previous results (Fig. 3b and Supplementary Fig. S4a), Pin1-depleted (DOXY) tumoral cells retained N3 expression, not influenced at all in the absence of CBDCA treatment (Fig. 9d, right panels).

Overall, these findings indicate that Pin1 inhibition impairs N3 signalling, ultimately resulting in sensitivity to chemotherapeutic drugs.

### Pin1/Notch3 *axis *activation is correlated with platinum resistance in HGSOC primary tumours

**Fig. 10:**
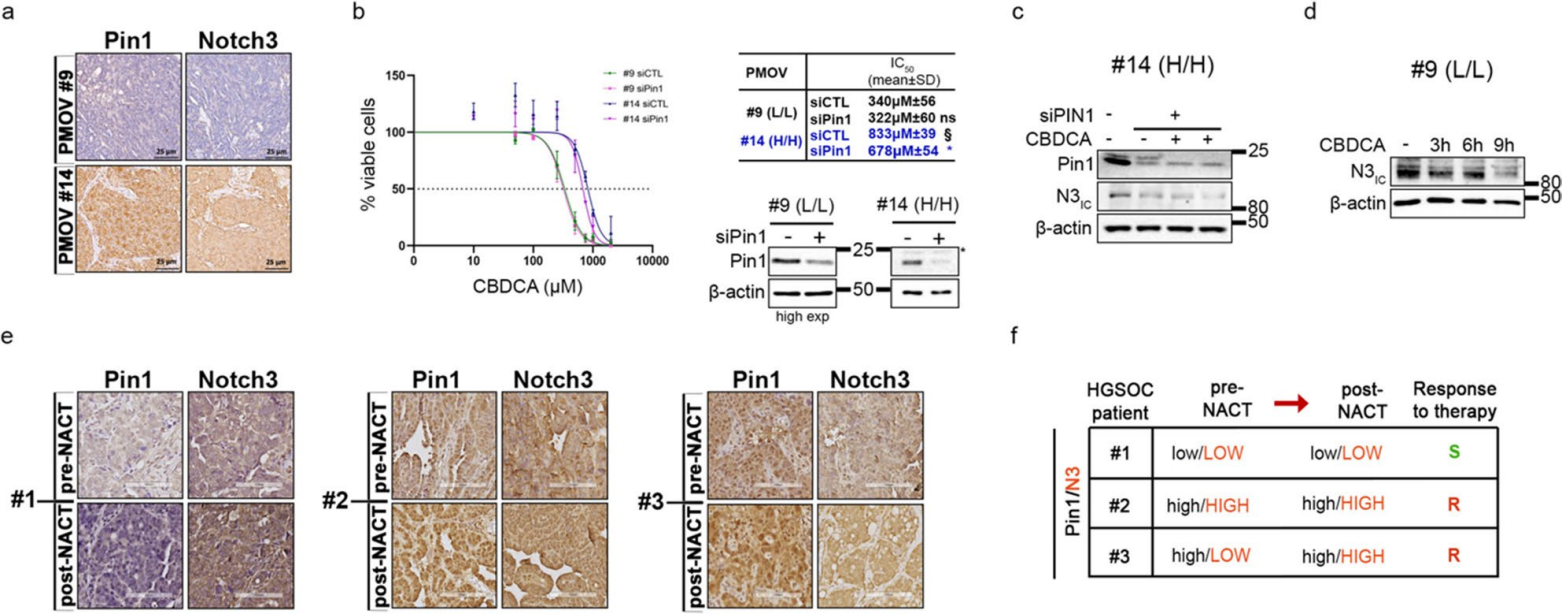
Pin1/Notch3 axis activation is correlated with platinum resistance in HGSOC primary tumours. **a** Representative Pin1/N3 IHC staining of HGSOC biopsies used for the isolation of the indicated (#) primary cells [preclinical model ovarian cancer (PMOV)]. Scale bar = 25 μm; original magnification, 20X. **b** CBDCA dose–response curves. PMOV#9 and PMOV#14 cells were transfected with control or Pin1-specific siRNAs. The results are expressed as the percentage of viable cells with respect to untreated cells, and the resulting IC50 is expressed as the mean value of three independent experiments ±SD. The expression of Pin1 with N3 is reported in the right inset. The difference between the control group (siCTL) and Pin1-specific siRNA (siPin1) within the indicated PMOV (#) (black: PMOV#9; blue: PMOV#14) is reported, as is the difference among the control groups (PMOV#9 siCTL vs PMOV#14). Statistical significance was determined by one-way ANOVA followed by Tukey's multiple comparisons test. **c** Immunoblotting analyses of N3 expression in PMOV#14 cells upon Pin1 genetic targeting induction (siPin1) with or without a suboptimal dose of CBDCA (6–9 h).** d** Immunoblotting analyses of N3 expression in PMOV#9 cells treated with or without a suboptimal dose of CBDCA (3-6-9 h). **e** Pre- and Post-NACT therapy, tumour biopsies from the indicated patients (#) were subjected to IHC for Pin1/N3. Scale bar = 100 μm; original magnification, 20X. **f** Table showing the correlation between Pin1/N3 protein expression shown in e and the platinum-based clinical response defined in accordance with international guidelines. S=sensible; R=resistant. ns=not significant P>0.05, *P≤0.05, **P≤0.01, §*P*≤0.001¸****P≤0.0001. Anti-β-actin antibody was used as a loading control. In **b**,** c**, and **d**, H and L refer to Pin1 and N3 expression (H=High and L = Low) as reported in Fig. 2

To translate our findings into a relevant preclinical model, we next used two primary chemo-naïve HGSOC cell lines, named PMOV#9 and PMOV#14 (Supplementary Table S1), which were isolated from fresh biopsies of HGSOC patients selected as described in Fig. 2: these cells were representative of low (L) or high (H) co-expression of both Pin1 and N3 proteins, respectively (Fig. 10a and Supplementary Fig. S15a).

As shown in Fig. 10b, PMOV#14 (H/H) is more resistant to CBDCA than PMOV#9 (L/L), confirming that the high expression of both Pin1 and N3 is correlated with a more aggressive phenotype.

Notably, in PMOV#14 (Pin1-high context), the genetic targeting of Pin1 induced a decrease in N3 protein levels (Fig. 10c), which was consistent with the observed re-sensitization to CBDCA (Fig. 10b), indicating that high protein levels of Pin1 are necessary to stabilize N3 under platinum pressure in primary tumours. Consistent with these data, CBDCA treatment of PMOV#9 cells (Pin1-low context) did not lead to an increase in N3 (Fig. 10d), likely because Pin1 is expressed at very low levels (Fig. 10a), thus explaining why Pin1 inhibition did not affect the CBDCA response (Fig. 10b).

To confirm these observations, we evaluated Pin1/N3 levels in HGSOC biopsies before and after CBDCA therapy (Fig. 10e, Supplementary Fig. S15b-d) and correlated them with their clinical platinum response (Fig. 10f). We performed IHC analysis on paired tumour samples obtained prior to the start of PT-based neoadjuvant chemotherapy (pre-NACT) treatment and at the time of interval debulking surgery (post-NACT) (Fig. 10e and Supplementary Table S1). The pre-NACT biopsies from patients #1 and #2 recapitulated PMOV#9 and #14, respectively, for the expression of the analysed proteins. Accordingly, the treatment did not affect the protein levels of Pin1/N3 in patient #1 (Fig. 10e), as we observed for PMOV#9 (Fig. 10d). Furthermore, patient #1 (Pin1-L/N3-L) was sensitive to treatment, whereas patient #2 (Pin1-H/N3-H) developed resistance (Fig. 10e), confirming our results (Fig. 10b). Notably, patient #3 is of particular interest, as its pre-NACT biopsy revealed high levels of Pin1 but low levels of N3 proteins. Interestingly, post-NACT, the levels of N3 increased (Fig. 10e), which correlates with a resistant phenotype (Fig. 10f), thus confirming the ability of Pin1 to regulate N3 stability and function under platinum pressure, ultimately resulting in platinum resistance.

## Discussion

Heterogeneity, insurgence of resistance to platinum-based chemotherapy, and recurrence still represent crucial issues to be addressed for the management of HGSOC-bearing patients [40]. Several studies depicted the interplay between different signaling pathways operating within tumour cells as a potential strategy to escape platinum-based chemotherapy response in HGSOC [33], thus enabling the discovery of potential druggable targets to develop more effective therapies.

In this context, we identified the Pin1/N3 *axis* as potentially predictive of poor response to standard platinum-based treatments in HGSOC patients.

Mechanistically, we investigated N3_ICD_ phospho-sites and their impact on N3_ICD_ stability. Our findings cover a largely unstudied layer of fine tuning and regulation of N3 in cancer, given that little is known about how phosphorylation affects N3 function [41]. Indeed, we mapped the phospho-residues responsible for Pin1 binding, which in turn prevents the N3_ICD_/GSK3β interaction. In particular, we demonstrated that once Pin1 targets the pS2033 site, it physically occupies the surrounding molecular space. This steric hindrance effectively sequesters the S2029 residue, protecting it from GSK3β and preventing its subsequent phosphorylation. Therefore, Pin1 acts as a critical negative regulator of GSK3β activity at this specific locus. Notably, our data indicate that the kinase GSK3β negatively affects N3_ICD_ stability, thus adding further information to previous studies which described GSK3β kinase either as a positive or a negative regulator of N3_ICD_ in other tumour contexts [42, 43]. While the role of GSK3β in HGSOC [44] remains controversial, aberrant activation of the PI3K/AKT/mTOR signalling pathway, including PTEN loss, which negatively affects GSK3β function, is frequently reported in OC [45]. Furthermore, several lines of evidence suggest that oncogenic processes targeting GSK3β activity result in the promotion of proliferative or invasive signals or the inhibition of apoptotic ones [46], which is consistent with our data. Indeed, we demonstrated that high Pin1 expression hinders GSK3β-dependent proteasomal degradation, mirroring findings in the context of breast cancer, where Pin1 sustains Notch1 by antagonizing the E3-ubiquitin ligase Fbxw7 [18].

In keeping with previous studies reporting frequent N3 overexpression in recurrent post-chemotherapy HGSOCs [47], we demonstrated that the Pin1/N3_ICD_ interaction increased under platinum pressure, first functionally leading to increased N3_ICD_ protein levels and finally resulting in the acquisition of a platinum drug-resistant phenotype. Our generated model of Carboplatin-resistant cells supported these data. Consistently, data from HGOSC primary tumours strongly support these findings. In particular, by comparing pre- vs. post NACT patients we observed that the combined high co-expression of both Pin1/N3 proteins correlated with poor response to platinum-based chemotherapy, thus being an interesting predictive response biomarker in HGSOC. Of clinical relevance, this may suggest a potential stratification of HGSOC, thus identifying patients expressing high levels of both Pin1 and Notch3 proteinsas the population who most likely could benefit from targeting this signalling.

While our data highlight a central role for Pin1 in modulating N3 driven platinum resistance, its broader impact likely involves additional pathways given that Pin1 sustains multiple cancer-driving targets over Notch receptors [17]. In keeping with this, the observed HGSOC re-sensitization to Platinum upon Pin inhibition via N3 destabilization by GSK3β may also be sustained by the known direct Pin1-regulatory activities on GSK3β itself [48, 49]. Interestingly, the synergistic antitumoral effect of N3 targeting and the antiangiogenic drug bevacizumab [50], which is commonly used in combination with first-line chemotherapy and as a maintenance strategy in HGSOC, suggests the possibility of extending Pin1 inhibitors to the overall management of HGSOC patients, thus allowing stratification before treatment.

From this holistic perspective, Pin1 targeting may also be useful for mimicking “BRCAness” conditions in HGSOC, as Pin1 has been shown to sustain the BRCA1 protein in other BRCA-associated cancers, including prostate cancer pancreatic, and triple-negative breast cancer [51]. Therefore, Pin1 inhibition potentially enhances the response to chemotherapy [33] and poly (ADP‒ribose) polymerase inhibitors (PARPis) [52], the latter being an effective maintenance strategy currently limited to a subset of HGSOC patients showing a “BRCAness” phenotype owing to their HRD^+^ (homologous recombination deficiency) *status*.

Taken together, our findings provide important evidence that Pin1 is an intriguing target that could be used to implement first-line and maintenance therapies for HGSOC patients. Interestingly, Pin1-deficient mice develop normally [53], suggesting that Pin1 downmodulation should avoid general toxicity. To our knowledge, only a few studies have evaluated Pin1 inhibition in HGSOC and have focused mainly on how it affects cancer cell proliferation [16, 54], thereby underscoring that the potential of Pin1 targeting in this context is far from fully elucidated. Remarkably, we demonstrate that Pin1 could be an actionable vulnerability in HGSOC, as its inhibition, combined with platinum treatment, impairs N3 protein expression and function, ultimately restoring chemosensitivity and reducing the tumour burden.

Clinically, several Pin1 inhibitors have been developed in recent years and tested for their potent anticancer efficacy [55]. Promising evidence has been derived from studies on the KPT-6566 molecule, a small Pin1 inhibitor able to selectively inhibit Pin1 and target it for degradation [20], thus effectively reducing lung dissemination in vivo without major toxicity [20]. Moreover, all-trans retinoic acid (ATRA), which induces Pin1 degradation [56], could be of particular interest, as it is an FDA-approved drug that is currently used for acute promyelocytic leukemia (APL)-bearing patients in combination with arsenic trioxide [57]. Since ATRA has relatively low systemic toxicity, expanding its use for the treatment of other cancer types has become crucial. As a result, the therapeutic potential of ATRA has been extensively studied in a variety of cancer types, thus allowing several clinical trials using ATRA, which are currently ongoing for the treatment of solid tumours [58]. However, additional studies are needed to strongly support its potential use as an appealing therapeutic strategy for HGSOC therapy. In this scenario, some limitations should be acknowledged in this study, mainly regarding the translational relevance. Despite promising in vitro and in vivo data obtained by using validated HGSOC established cells and primary tumours from HGSOC patients, this study used a small number of models also limited to 2D cultures which often lose the complexity of the human pathology. Implementing HGSOC cohort consistency by extending our *in-house* dataset of HGSOC patients is essential for the establishment of clinically relevant models, including HGSOC patients-derived organoids, able to mimic key architecture, stromal/ECM cues and cellular heterogeneity of the disease, thus representing the best tool to better understand the correlation between Pin1/Notch3 expression and CBDCA responses in HGSOC, and finally resulting in personalized applications in clinic. Consistently, despite the observed paclitaxel cross-resistance in our generated HGSOC-resistant cells, further assays addressing whether the Pin1/N3 axis also affects taxane response will be essential to clarify treatment option for patients.

## Conclusion

Overall, this study provides a strong rationale for further investigation of Pin1 pharmacological inhibition and platinum-based combinations in HGSOC-bearing patients who rely on Pin1/N3 *axis* activation to survive and acquire a resistant phenotype. In this context, we suggest a novel role of the combined high Pin1/N3 co-expression in predicting the platinum response of HGSOC patients before clinical recurrence, which could be exploited to develop new diagnostic and therapeutic tools, thus opening innovative perspectives to support clinicians in HGSOC management.

## Supplementary Information

Supplementary Material 1.

Supplementary Material 2.

## Data Availability

All data in this study are available within the Article and Supplementary Information or from the corresponding authors upon reasonable request.
