# Supplementary material for: Prolyl-isomerase Pin1 drives platinum resistance by regulating Notch3 stability and function in ovarian cancer
Source: J Exp Clin Cancer Res. 2026 Feb 11;45:71. doi: 10.1186/s13046-026-03658-x (PMC12998371; doi:10.1186/s13046-026-03658-x)
Supplement: Supplementary file 1 — Supplementary Material 1. [file 13046_2026_3658_MOESM1_ESM.docx]

**Supplementary Material**

**Prolyl-isomerase Pin1 drives platinum resistance by regulating Notch3 stability and function in ovarian cancer**

**Authors**

Maria Valeria Giuli^1^, Angelica Mancusi^2^, Bianca Natiello^3^, Samuele Di Cristofano^3^, Rebecca Reali^3^, Maria Gemma Pignataro^4^, Daniel D’Andrea^5^, Laura Di Magno^3^, Carmine Nicoletti^6^, Alessandra Giorgi^7^, Alberto Macone^7^, Serena Camerini^8^, Marialuisa Casella^8^, Giovanna Peruzzi^9^, Sabrina Zema^3^, Gianluca Canettieri^3^, Federica Tomao^10^, Innocenza Palaia^10^, Angelina Pernazza^4^, Alessandra Rustighi^11,12^, Rocco Palermo^3^, Domenico Raimondo^3^, Alessandra Monti^13^, Nunzianna Doti^13^, Giulia d’Amati^4^, Giannino Del Sal^11,12,14^, Isabella Screpanti^3^, Claudio Talora^3*^, Diana Bellavia^3^, Saula Checquolo^1*^

^1^Department of Medico-Surgical Sciences and Biotechnology, Sapienza University of Rome, Laboratory affiliated with Istituto Pasteur Italia-Fondazione Cenci Bolognetti, Latina, Italy; ^2^Department of Pediatric Hematology and Oncology, Cell and Gene Therapy, Bambino Gesù Children's Hospital, IRCCS, Rome, Italy; ^3^Department of Molecular Medicine, Sapienza University of Rome, Laboratory affiliated with Istituto Pasteur Italia-Fondazione Cenci Bolognetti, Rome, Italy; ^4^Department of Radiological, Oncological and Pathological Sciences, Sapienza University of Rome, Rome, Italy; ^5^School of Engineering Mathematics and Technology, University of Bristol, Bristol, United Kingdom; ^6^Department of Anatomy, Histology, Forensic Medicine and Orthopaedics, Sapienza University of Rome, Rome, Italy; ^7^Department of Biochemical Sciences, Sapienza University of Rome, Rome, Italy; ^8^Core Facilities, Istituto Superiore di Sanità, Rome, Italy; ^9^Center for Life Nano- & Neuro-Science@Sapienza, Istituto Italiano di Tecnologia, Rome, Italy; ^10^Department of Gynecological, Obstetrical and Urological Sciences, Sapienza University of Rome, Rome, Italy; ^11^Department of Life Sciences, University of Trieste, Trieste, Italy; ^12^International Centre for Genetic Engineering and Biotechnology (ICGEB), Area Science Park-Padriciano, Trieste, Italy; ^13^Istituto di Biostrutture e Bioimmagini, IBB-CNR, Naples, Italy;^14^IFOM ETS, the AIRC Institute of Molecular Oncology, Milan, Italy.

These authors contributed equally: Giuli Maria Valeria, Mancusi Angelica and Natiello Bianca.

These authors contributed equally: Bellavia Diana and Checquolo Saula.

**Corresponding authors*:** Saula Checquolo (SC): [saula.checquolo@uniroma1.it](mailto:saula.checquolo@uniroma1.it); Claudio Talora (CT): [claudio.talora@uniroma1.it](mailto:diana.bellavia@uniroma1.it)

**
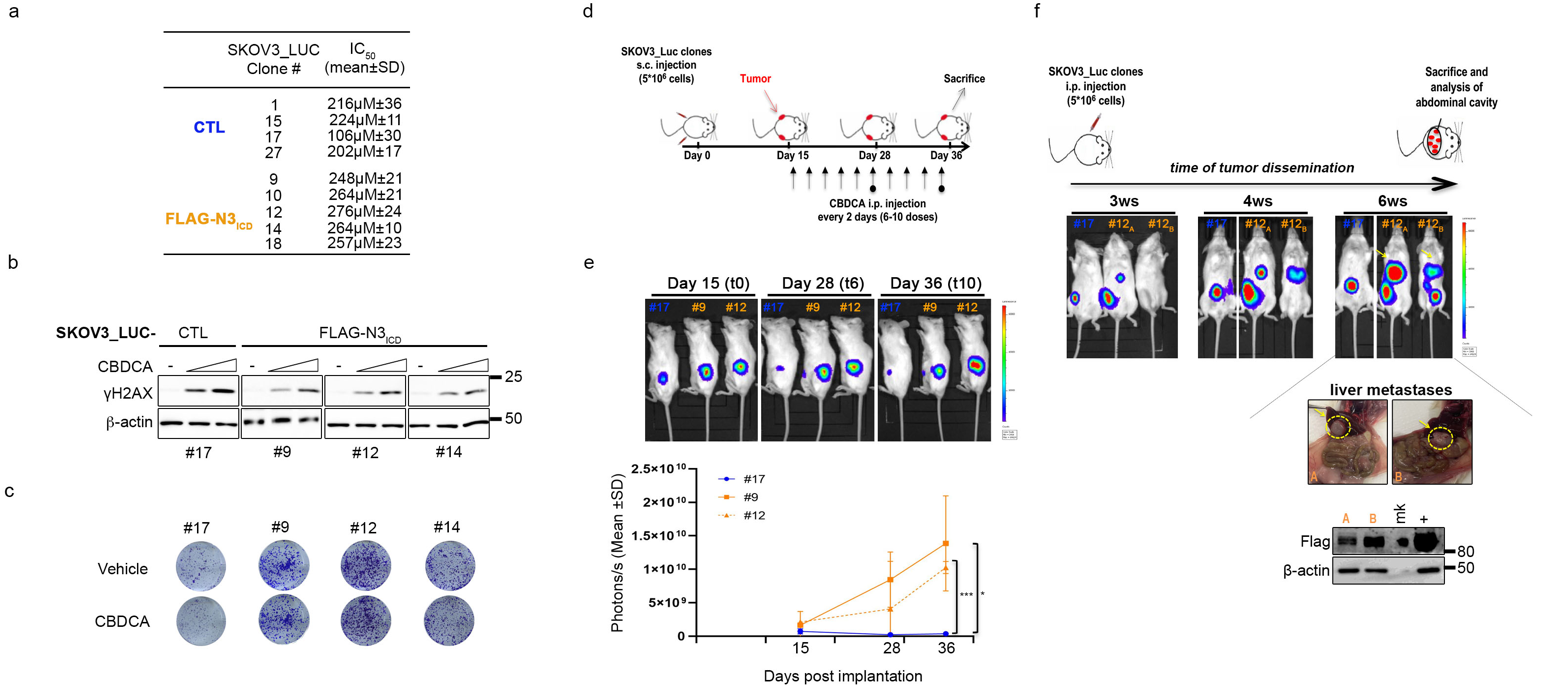
Supplementary Figures**

**Figure S1. Effects of N3 overexpression in OC both *in vitro* and *in vivo.***

**a.** Table reporting the average IC50 value of three independent experiments ±SD of SKOV3-LUC selected clones (#) subjected to increasing doses of CBDCA for 72 h and then released in drug-free medium for an additional 24 h, as reported in Fig. 1b. **b.** Immunoblotting analyses of the expression of S139 phosphorylated histone H2AX (γH2AX), a marker of DNA damage in SKOV3-LUC-CTL *vs* the indicated FLAG-N3_ICD_ clones (#) treated with increasing doses of CBDCA (IC_25_ and IC_50_) as described in **a. c.** A representative colony formation assay was performed on the same cells described in **a.** which were treated or not treated with a suboptimal dose of CBDCA for 72 h and then released for another 72 h. **d-f.** NSG mice (n=4 for each group) bearing the selected clones (#) injected subcutaneously (s.c.) received intraperitoneal injections (i.p.) of CBDCA (20 mg/kg) every two days (treatment scheduling in **d**). Tumour growth was monitored via optical imaging at the indicated times. **e.** Representative images (upper panels) and quantitative analysis (lower panels) of luciferase activity at the indicated times. Statistically significant differences in average radiance (expressed as the mean ± SD) are indicated. P-values were calculated via one-way ANOVA followed by Tukey's multiple comparisons test. **f.** NSG mice (n=4 for each group) were intraperitoneally (i.p.) injected with the selected SKOV3_LUC clones (#). Tumour dissemination was evaluated by optical imaging at indicated times. At the end of the experiment, the abdominal cavities were evaluated, and macroscopic evidence of liver metastases in mice A and B (injected with clone #12) is shown. Immunoblot analysis of liver metastases probed with an anti-Flag antibody to detect FLAG-N3_ICD_ levels.

ns=not significant P>0.05, *P≤0.05, **P≤0.01, ***P≤0.001¸****P≤0.0001.

Anti-β-actin antibody was used as a loading control. ws: week; +: positive control (extract from #12).

**
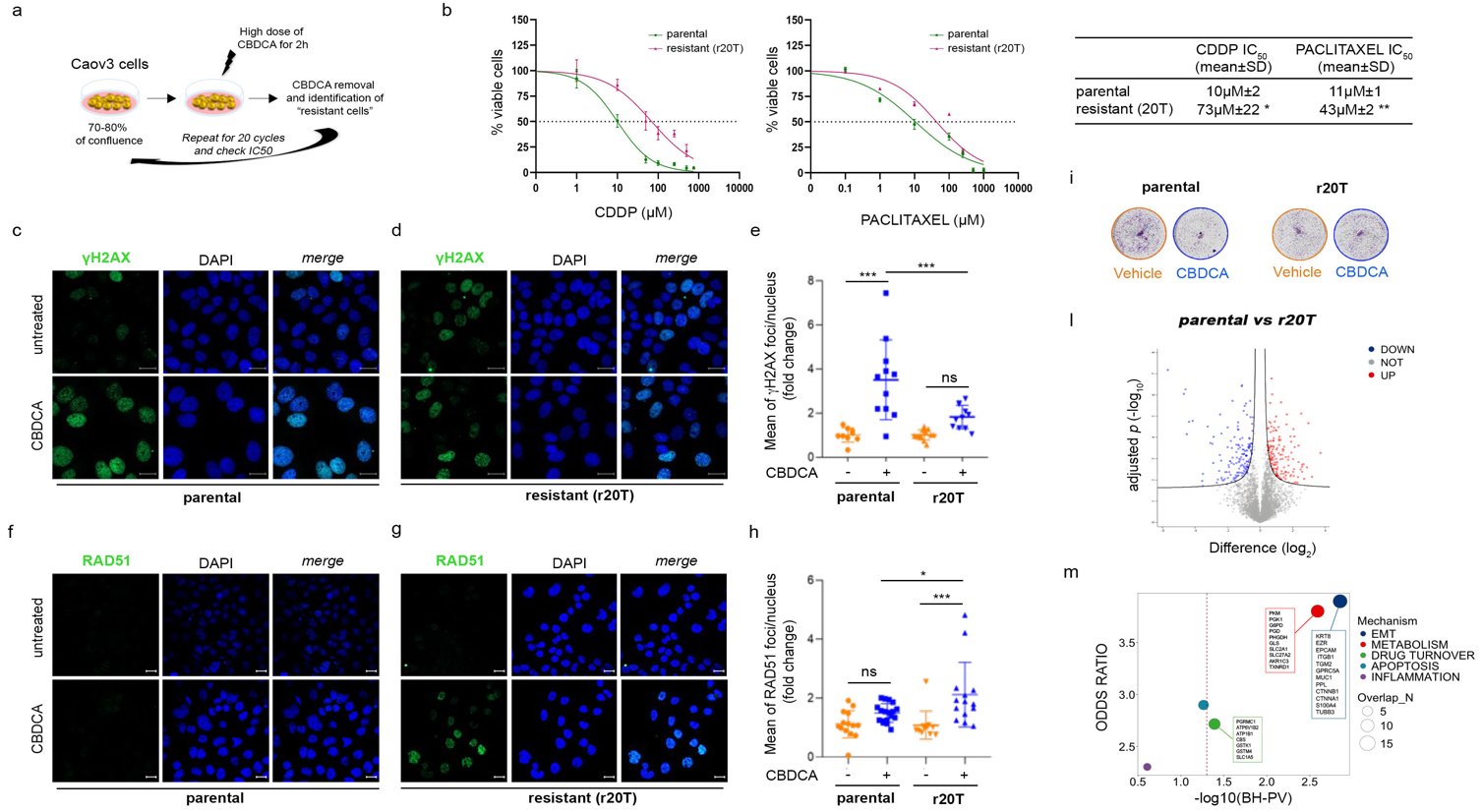
**

**Figure S2. Generation and characterization of CBDCA-resistant HGSOC cells.**

**a.** Experimental design used in the generation of Caov3 CBDCA-resistant cells (r20T) by pulse method (described in Material and methods section). **b.** Cisplatin (CDDP) (left panel) and Paclitaxel (middle panel) dose-response curves on parental and r20T cells, as indicated. Cells were subjected to increasing doses of: i) CDDP for 16 h and then released in drug-free medium for an additional 24 h or ii) Paclitaxel for 48 h. The results are expressed as the percentage of viable cells with respect to untreated cells, and the resulting IC_50_ is expressed in the table (right panel) as the mean value of three independent experiments ±SD. The difference between parental and r20T cells is reported for each drug. Statistical significance was determined by unpaired t-test. **c-h.** Immunofluorescence analyses on the same cells described in **b.**, upon treatment or not with CBDCA by using a dose corresponding to the IC_25_ of parental cells indicated in Fig. 1i for 72 h and then released for another 24 h (γH2AX) or 6 h (RAD51). **c,d,f,g.** Representative images of S139 phosphorylated histone H2AX (γH2AX) (**c,d**) and RAD51 (**f,g**). Green fluorescence indicates the endogenous levels of γH2AX (**c,d**) and RAD51 (**f,g**). Cell nuclei were stained with DAPI. Scale bar: 20 μm.**e,h.** Dot plot representing the mean of γH2AX (**e**) and RAD51 foci (**h**)/nucleus ± SD. Results are expressed as fold change with respect to untreated cells. Significant differences were computed via one-way ANOVA. **i.** A representative colony formation assay of the same cells described in **b.** which were treated or not treated with a suboptimal dose of CBDCA for 72 h and then released for another 72 h. **l.** Volcano plots showing differentially expressed (DE) proteins (q < 0.05, S0=0.1): Caov3 model with 4343 proteins detected in at least 3 replicates in one group, n=5 biological replicates for each group. The negative log10 P values plotted against the differences between the geometric means of the abundances are reported. Dots represent individual proteins. Red and blue dots: up- and downregulated proteins. Gray dots: non significantly differentially abundant proteins. **m.** Enrichment analysis comparing DE proteins in r20T *vs* Caov3 parental with known mechanisms involved in PT-resistance (EMT, Metabolism, Drug Turnover, Apoptosis, and Inflammation) by *Huang and colleagues*(1), highlighting significant enrichment in EMT, Metabolism and Drug Turnover (DE proteins are indicated in the coloured boxes). Results are presented as −log₁₀-transformed P values after Benjamini–Hochberg (BH) correction for multiple testing, together with odds ratios. Point size indicates the number of differentially expressed genes associated with each mechanism. Statistical significance was assessed using Fisher’s exact test, with a threshold of BH < 0.05.

ns=not significant P>0.05, *P≤0.05, **P≤0.01, ***P≤0.001¸****P≤0.0001.

**
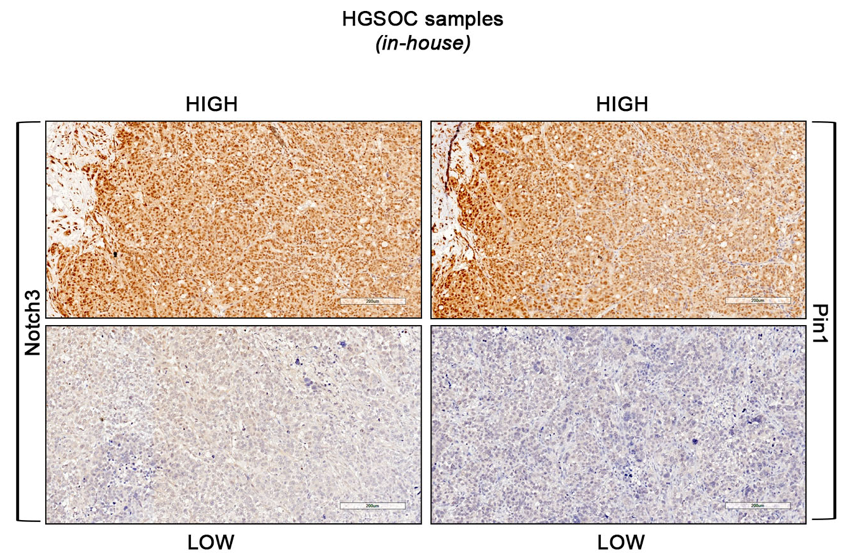
**

**Figure S3. Full resolution of IHC staining.**

Full resolution of the HGSOC representative images of IHC staining reported in Figure 2c (scale bar = 200μM; original magnification, 20X).

**
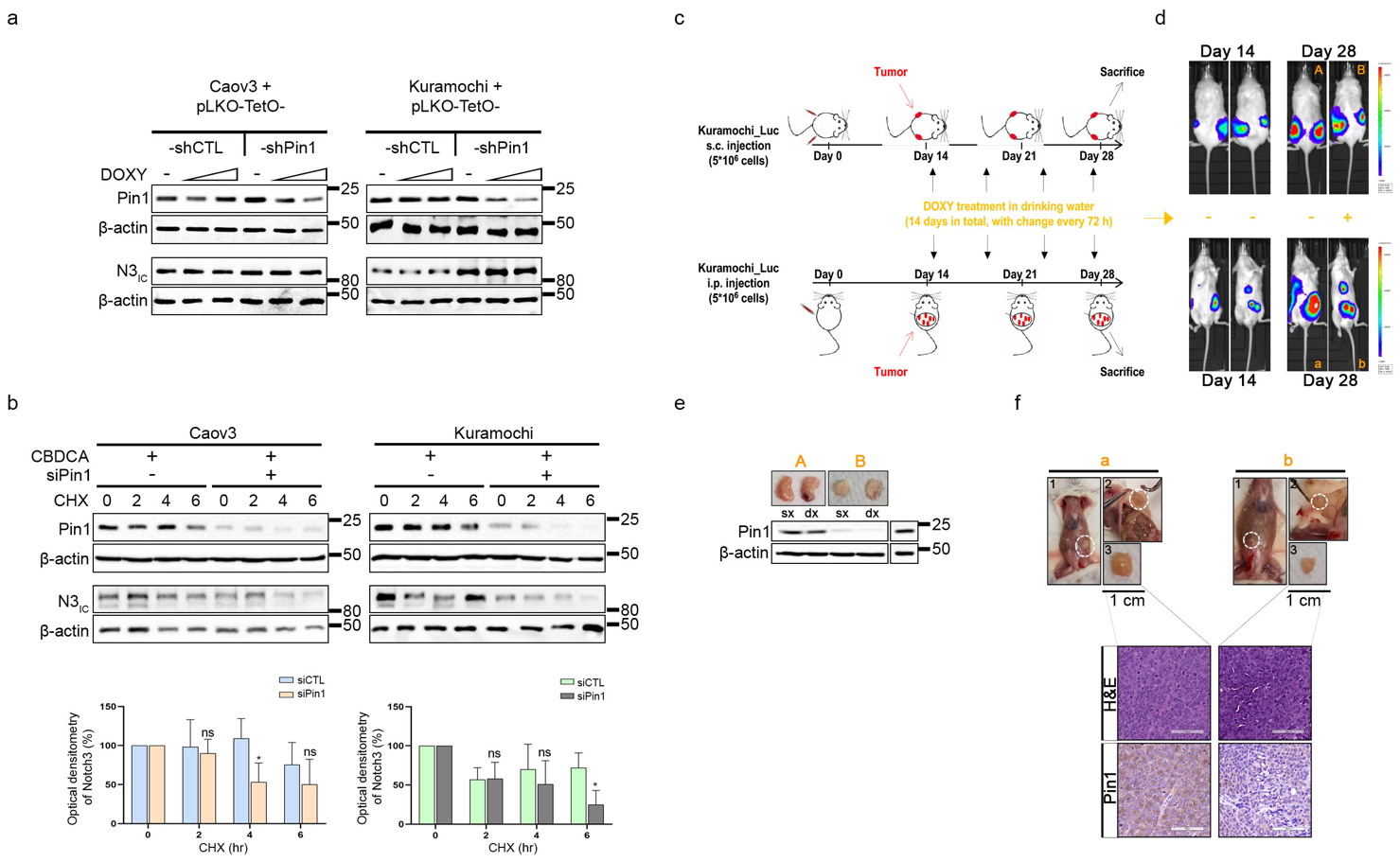
**

**Figure S4. Effects of Pin1 suppression *in vitro* and *in vivo*.**

**a.** Immunoblotting analyses of N3 expression in Caov3+pLKO-TetO-shCTL vs -shPin1 (left panel) and Kuramochi+pLKO-TetO-shCTL vs -shPin1 (right panel) cells upon shPin1 induction (DOXY, 48h). **b.** Immunoblotting analyses of N3 protein stability Caov3 (left panel) and Kuramochi (right panel) cells treated with a suboptimal dose of CBDCA (6 h) and transfected with control (siCTL) or Pin1-specific siRNA (siPin1). The cells were analysed after time-course treatment with cycloheximide (CHX) for 0-2-4-6 h (upper panels). Densitometric analyses of β-actin-normalized N3 protein levels are shown as the mean value of four (Caov3) or three (Kuramochi) independent experiments ±SD (right panels). The results are expressed as percentages with respect to time 0. The difference between siCTL and siPin1 is reported. Statistical significance was computed for each time point via an unpaired t test. **c-f.** NSG mice (n=4 for group) bearing Kuramochi+pLKO-TetO-shPin1 cells subcutaneously (s.c.) or intraperitoneally (i.p.) injected received doxycycline treatment in the drinking water every 72 h (mice B and b) or not (mice A and a) (treatment scheduling in **c**). Tumour growth was monitored via optical imaging at the indicated times. **d.** Representative images of luciferase activity at the indicated times. At the end point, the posterior flank masses (**e**) and abdominal cavities (**f**) were evaluated **e.** Representative images of explanted tumour masses (upper panels) subjected to immunoblot analysis of Pin1 expression (lower panel). **f.** Representative macroscopic evidence of intraperitoneal tumour masses (upper panels) subjected to H&E and Pin1 immunohistochemical staining (lower panels). Scale bar = 100μm - Original magnification, 20X.

ns=not significant P>0.05, *P≤0.05, **P≤0.01, ***P≤0.001¸****P≤0.0001.

In **a**, **b**, **e**, Anti-β-actin antibody was used as a loading control.

**
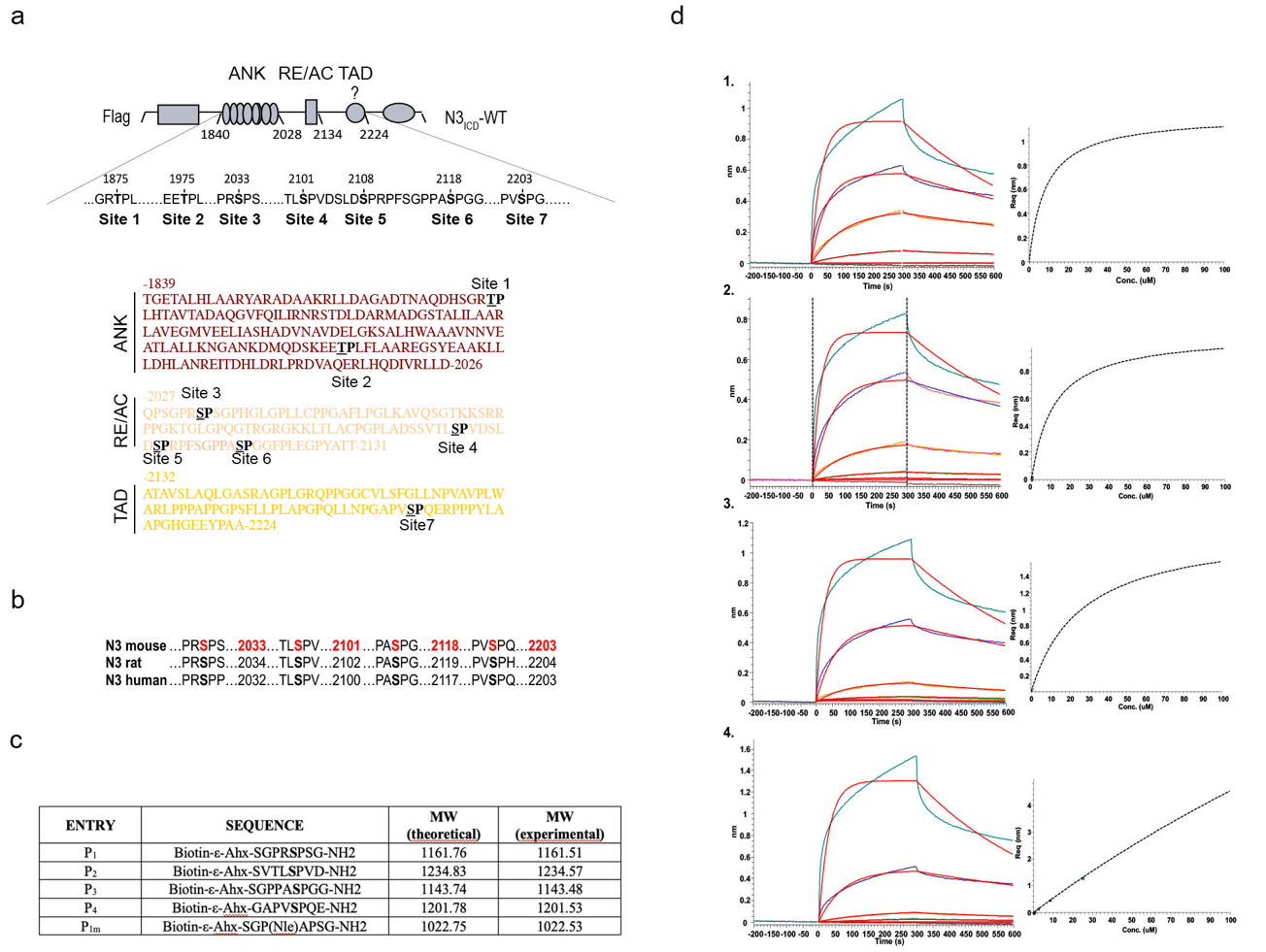
**

**Figure S5. Peptides mimicking the amino acid sequences of N3_ICD_ were identified as Pin1 substrates.**

**a.** Illustration of the ANK (ankyrin), RE/AC (repression/activation)(2), and TAD (transcriptional activation domain) regions of N3_ICD_ showing the seven putative Pin1 consensus motifs (in bold). **b.** Sequence alignment of the four identified Pin1 consensus motifs on the N3_ICD_ protein across different species. In **a** and **b**, numbering refers to UniProtKB entry Q61982. **c.** List and sequences of the peptides used in this study. The table also reports the theoretical and experimental molecular weights (MWs) as monoisotopic masses. All peptides are C-terminally amidated (-NH2) and biotinylated at the N-terminus using 6-aminohexanoic acid (ε-Ahx) as a linker. Phosphoserine is reported as S*, the norleucine residue is reported as NLeu, and the linker 6-aminohexanoic acid is reported as ε-Ahx. **d.** BLI binding experiments: Curve fits were performed via a 1:1 interaction model (left panels), and KD values were determined from steady-state binding levels over the range of analyte concentrations (right panels).

**
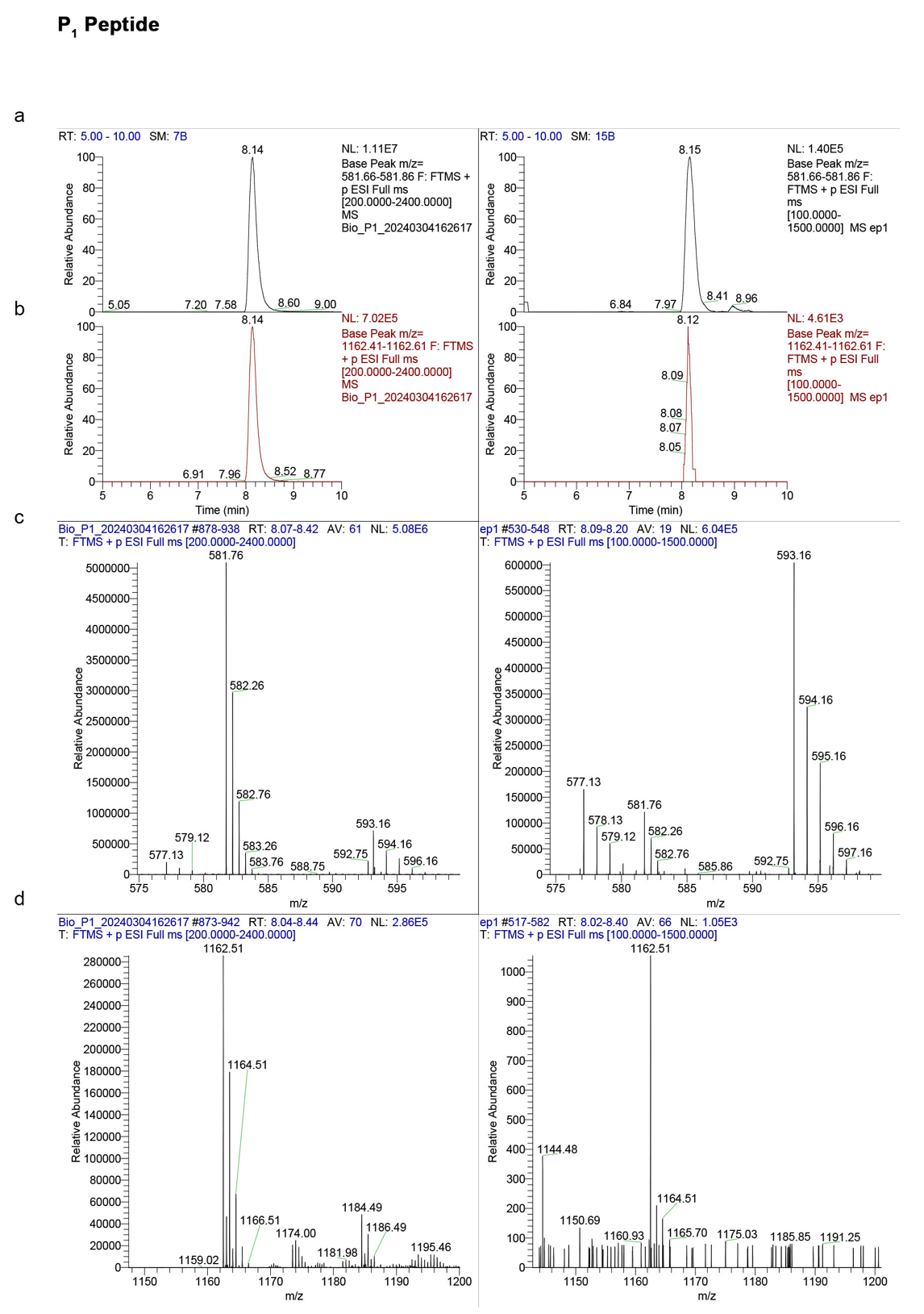
**

**Figure S6. LC‒MS analysis after pull-down experiments between P_1_ and Pin1.**

**a-d.** Each panel shows the MS spectra of the peptides purified to 0.1 µg, used as a reference (**a-d**, left panels), and of the unknown samples obtained from the pull-down experiments (**a-d**, right panels). The extracted base peaks for P_1_ fall within the *m/z* range of 581.66--581.86 (z =2, **a**) and 1162.41--1162.61 (z =1, panel **b**). The tR values of the extracted base peaks agreed with those of the reference, and MS analysis revealed that the predicted masses at *m/z were as follows*: 581.76 [M+2H]2+, 593.16 [M+H+Na]2+ (**c**) and 1162.51 [M+H]+ (**d**).


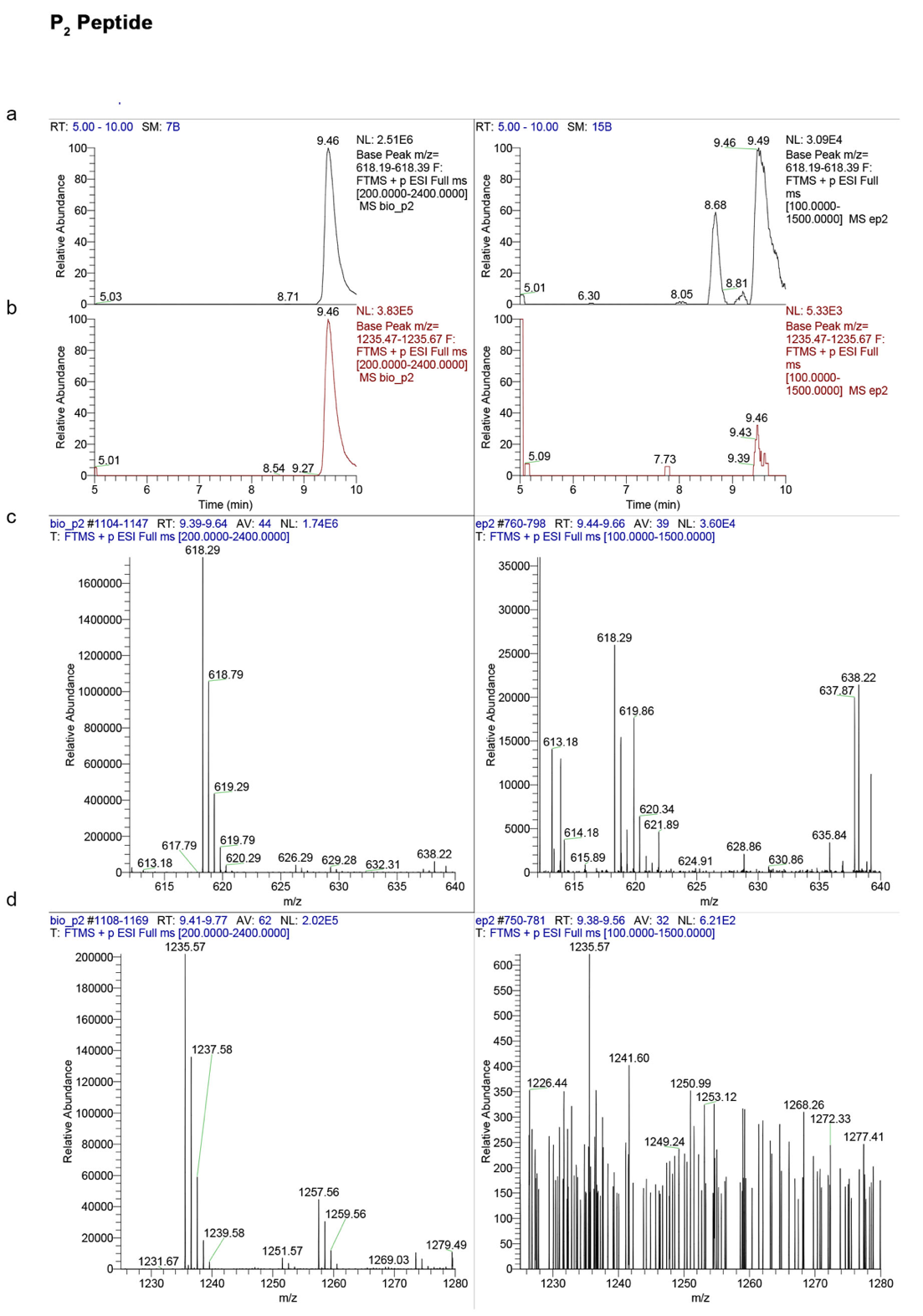


**Figure S7. LC‒MS analysis after pull-down experiments between P_2_ and Pin1.**

**a-d.** Each panel shows the MS spectra of the peptides purified to 0.1 µg, used as a reference (**a-d**, left panels), and of the unknown samples obtained from the pull-down experiments (**a-d**, right panels). The extracted base peaks for P_2_ fall within the *m/z* range of 618.19--619.39 (z =2, panel **a**) and 1235.47--1235.67 (z =1, panel **b**). The tR values of the extracted base peaks agreed with those of the reference, and MS analysis revealed that the predicted masses at *m/z* were 618.29 [M+2H]2+ (**c**) and 1135.57 [M +H]+ (**d**).


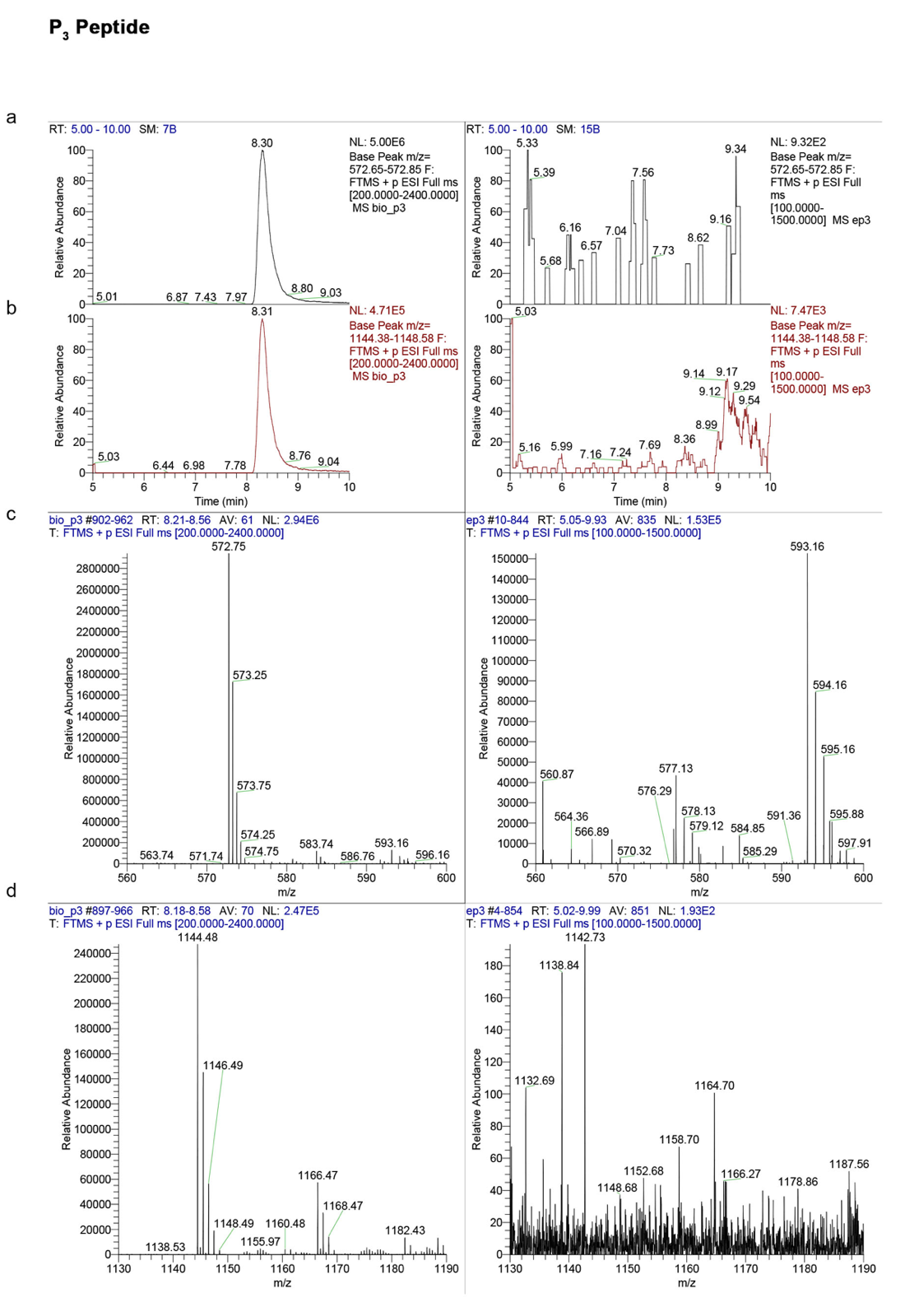


**Figure S8. LC‒MS analysis after pull-down experiments between P_3_ and Pin1.**

**a-d.** Each panel shows the MS spectra of the peptides purified to 0.1 µg, used as a reference (**a-d**, left panels), and of the unknown samples obtained from the pull-down experiments (**a-d**, right panels). The extracted base peaks for P_3_ fall within the *m/z* range of 572.65--572.85 (z =2, panel **a**) and 1144.38--1148.58 (z =1, panel **b**). For P_3,_ no significant peak was observed in the extracted base peaks, and the expected masses were not found in the MS spectra (**c, d**).


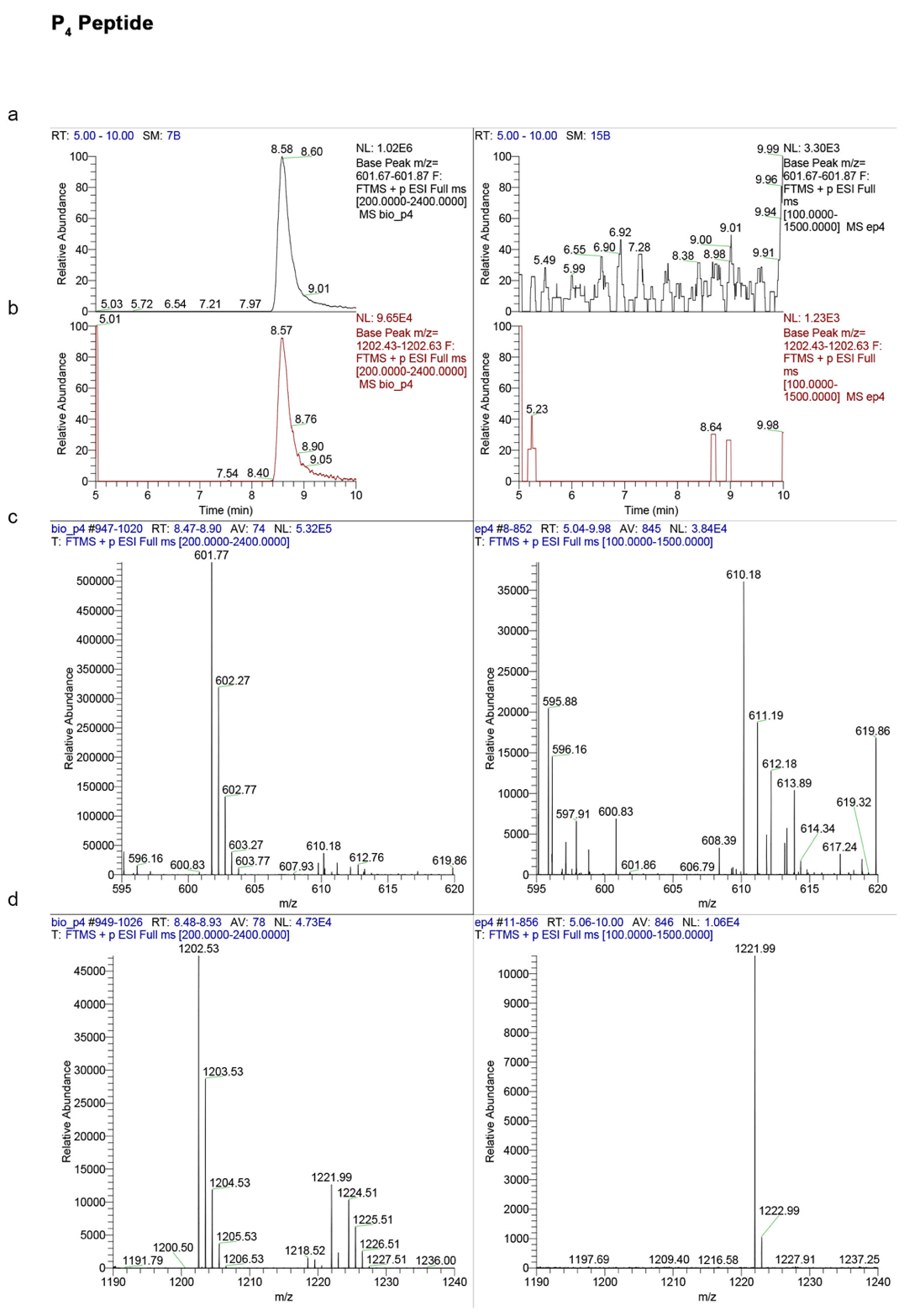


**Figure S9. LC‒MS analysis after pull-down experiments between P_4_ and Pin1.**

**a-d.** Each panel shows the MS spectra of the peptides purified to 0.1 µg, used as a reference (**a-d**, left panels), and of the unknown samples obtained from the pull-down experiments (**a-d**, right panels). The extracted base peaks for P_4_ fall within the *m/z* range of 601.67--601.87 (z =2, panel **a**) and 1202.53--1202.63 (z =1, panel **b**). For P_4,_ no significant peak was observed in the extracted base peaks, and the expected masses were not found in the MS spectra (**c, d**).

**
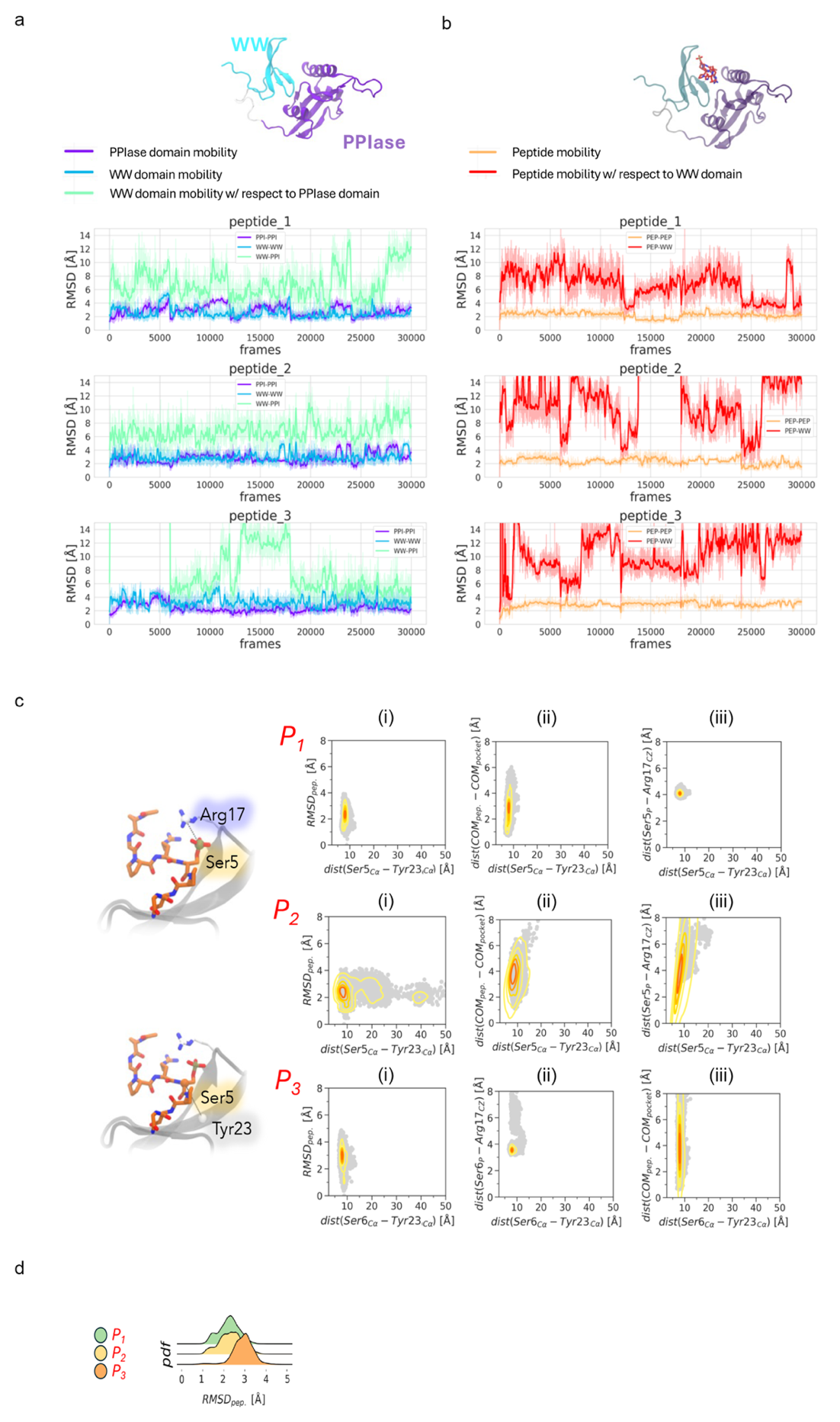
**

**Figure S10. Peptide RMSD and dCOM analyses.**

**a,b.** Time course of the peptide root mean square deviation (RMSD) throughout independent MD simulations. **c.** Bidimensional plots computed via multiple MD simulations of (i): C-α of phospho-Ser6 and C-α of Tyr23 vs. peptide-RMSD; (ii) C-α of phospho-Ser6 and C-α of Tyr23 vs. center of mass (dCOM) of peptides and the WW domain binding region; (iii) C-α of phospho-Ser6 and C-α of Tyr23 vs. the C-zeta of Arg17 and C-α of phospho-Ser6. The upper and lower insets represent the C-zeta of Arg17–C-α of phospho-Ser6 and the C-α of phospho-Ser6–C-α of the Tyr23 distance, respectively. **d.** The binding stability of Pin1–peptide complexes was assessed via the “*peptide RMSD*” metric, which was calculated as the root mean square deviation of the peptide Cα atoms relative to their predicted docking-predicted binding mode after superposition with the Pin1 WW domain. The probability distributions of peptide RMSD were analysed to evaluate binding stability across simulations.

**
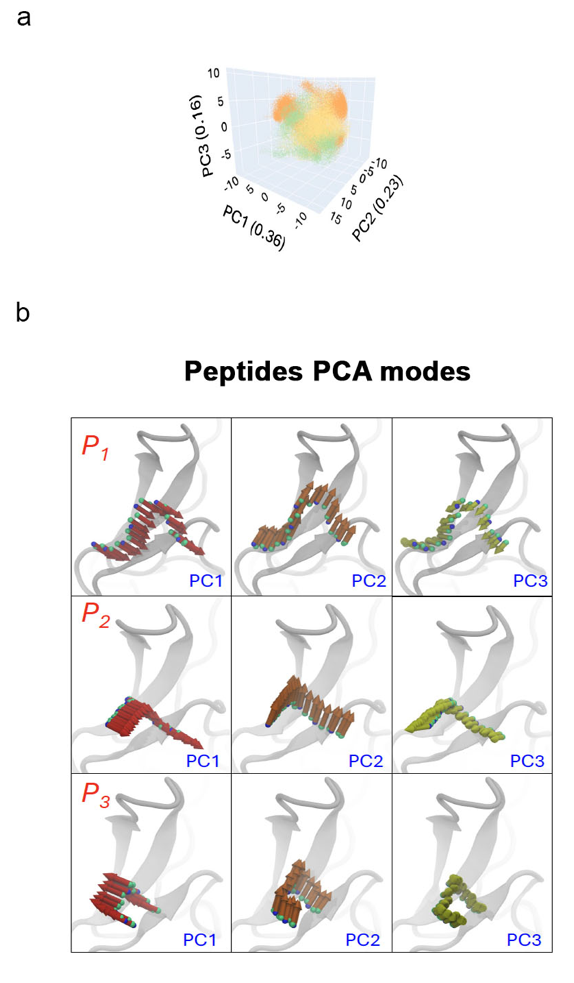
**

**Figure S11. PCA analyses.**

**a.** Principal component analysis of peptide–Pin1 complexes reveals distinct dynamic behaviours.

Essential dynamics were evaluated by performing principal component analysis (PCA) on the peptide backbone conformations pooled from five independent simulations for each complex. The principal components PC1, PC2, and PC3 accounted for 36%, 23%, and 16% of the total variance, respectively. The P_1_ and P_2_ peptides exhibited limited similarity in their dynamic behaviour, whereas P_3_ occupied extreme regions of the essential subspace, indicating distinct motions. P_1_ explored concerted movements toward key binding regions of the Pin1 WW domain (loop I, β1–β3), enhancing stable interactions. In contrast, P_2_ and P_3_ showed motions directed toward the solvent, with only minor contributions toward loop I (notably PC3 for P2 and PC2 for P3), which is consistent with partial stabilization via pSer6–Arg17 interactions. These data suggest that sequence-specific differences in peptide dynamics underlie the reduced binding stability observed for P_2_ and P_3_. **b.** Porcupine plots depicting the simulated motion of peptides in the top three PCA modes, shown as arrows originating from the backbone atoms of the peptides. The orientation of the arrow indicates the direction of motion of the atoms to which they are attached, and their length indicates the amplitude of this motion. The figure was created by using the NMWiz plugin in VMD.

**
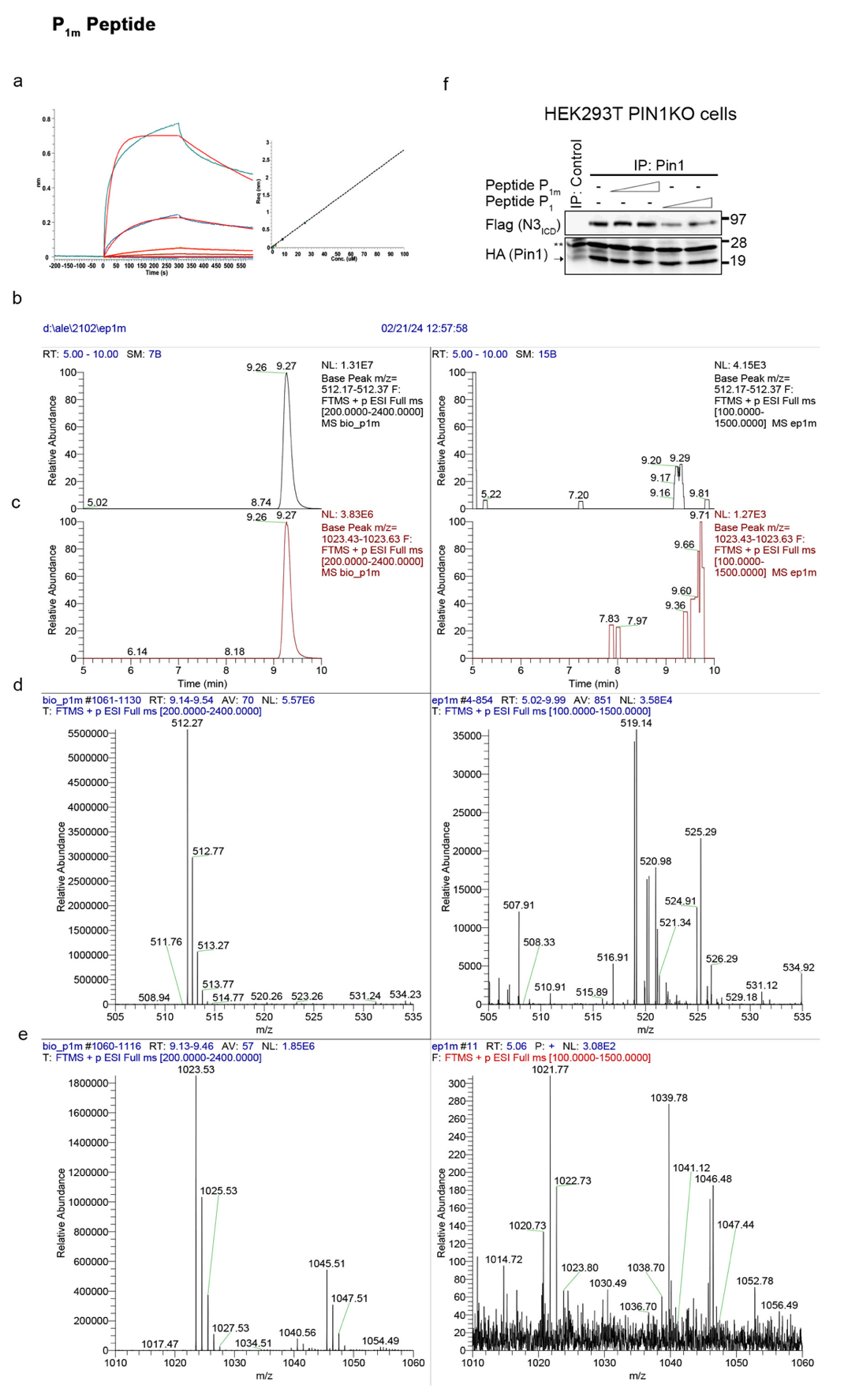
**

**Figure S12. Peptide P_1m_ characterization.**

**a.** BLI binding experiments: Curve fitting was performed via a 1:1 interaction model (left panel), and the KD values were determined from steady-state binding levels over the range of analyte concentrations (right panel). **b-e.** LC‒MS after pull-down experiments between P_1m_ and Pin1**.** Each panel shows the MS spectra of the peptides purified to 0.1 µg, used as a reference (**b-e**, left panels), and of the unknown samples obtained from the pull-down experiments (**b-e**, right panels). The extracted base peaks for P_1m_ fall within the *m/z* range: 512.17--512.37 (z=2, **b**) and 1023.43--1023, 63 (z=1, **c**). For P_1m,_ no significant peak was observed in the extracted base peaks, and the expected masses were not found in the MS spectra (**c, d**). **f.** Co-IP analyses of exogenous ^HA^Pin1 and ^FLAG^N3_ICD_ proteins in HEK293T Pin1KO cells in the presence of increasing doses of P_1m_ and P_1_.

**
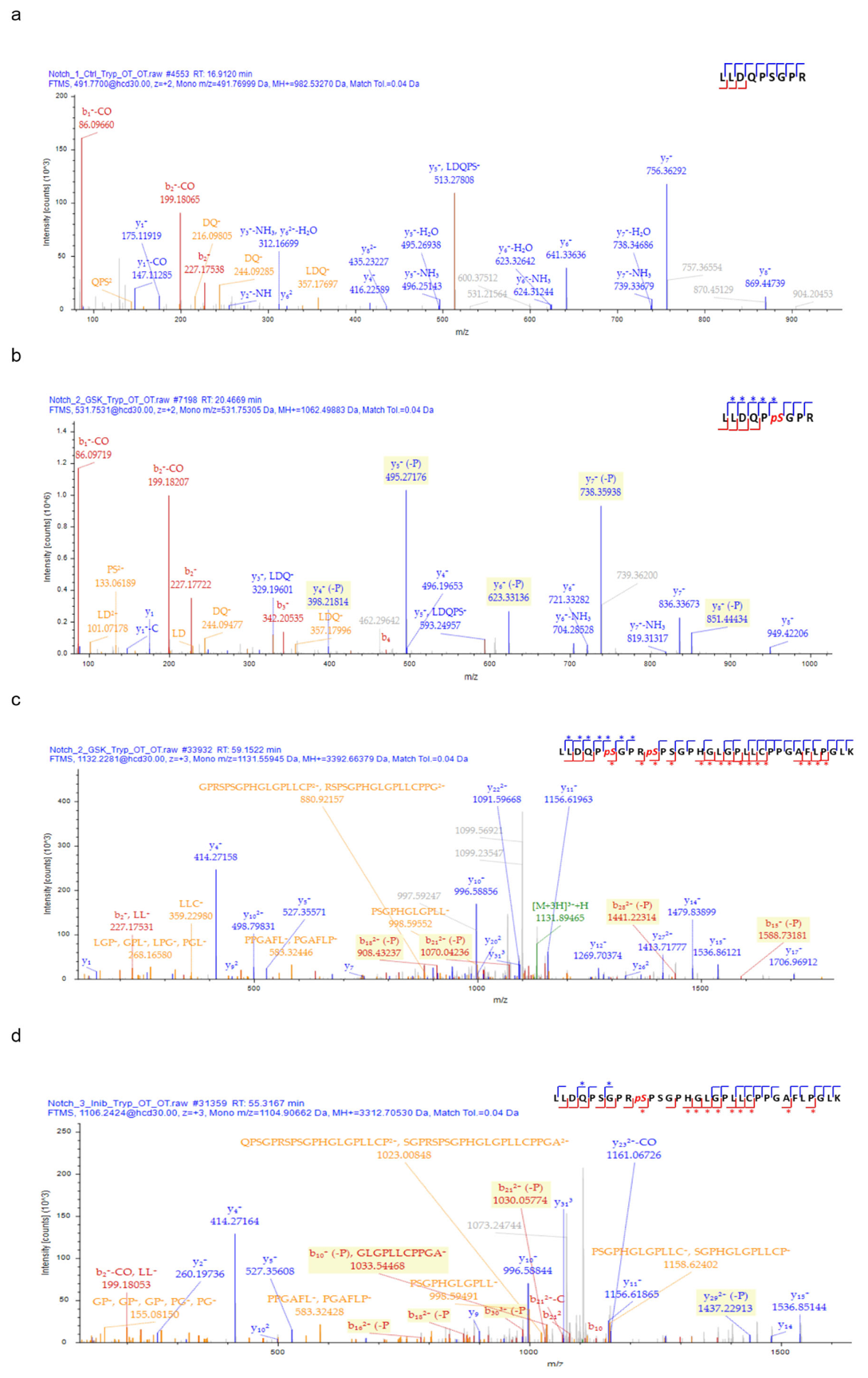
**

**Figure S13. LC‒MS/MS analysis of N3_ICD_.**

**a-d.** MS/MS spectra of ^FLAG^N3_ICD_-wt in the absence (sample 1) or in the presence of ^HA^GSK3β, alone (sample 2) or with CHIR99021 (2h) (sample 3). **a.** MS/MS spectrum of the (2024--2032) unmodified peptide detected in sample 1. **b.** MS/MS spectrum of the (2024--2032) peptide, phosphorylated at S2029, detected in sample 2. **c.** MS/MS spectrum of the (2024--2055) peptide phosphorylated on both S2029 and S2033 and detected in sample 2. **d.** MS/MS spectrum of the (2024--2055) peptide phosphorylated at S2033, which was detected in sample 3. In each spectrum, the inner panel contains the peptide sequence, with the blue and red lines indicating the matched y and b fragment ions, respectively, and the stars mark ions carrying a phosphate group. The yellow highlighted squares indicate ions derived from phosphoric acid neutral loss.

**
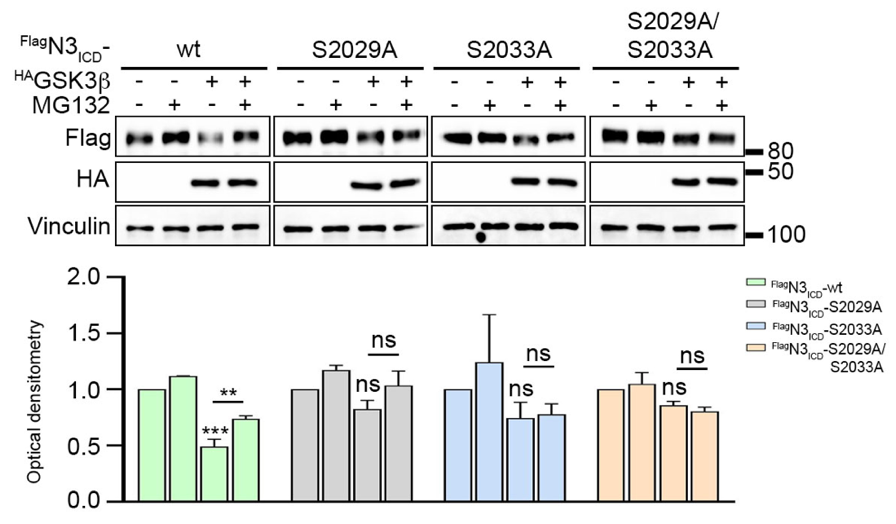
**

**Figure S14. Responsiveness to proteasomal degradation of ^FLAG^N3ICD-wt non phosphorylable mutants.**

**a.** Immunoblotting analyses (upper panel) of ^FLAG^N3_ICD_-wt and non phosphorylable mutants in the presence or absence of the highest dose of ^HA^GSK3β plasmid followed by MG132 (4 h). Densitometric analyses (lower panel) of vinculin-normalized N3 levels as shown as the mean value of three independent experiments ±SD. The results are expressed as percentages with respect to the corresponding control (no ^HA^GSK3β and no MG132). Statistical significance was determined by one-way ANOVA followed by Tukey's multiple comparisons test.

Anti-vinculin was used as a loading control.

ns=not significant P>0.05, *P≤0.05, **P≤0.01, ***P≤0.001¸****P≤0.0001.

**
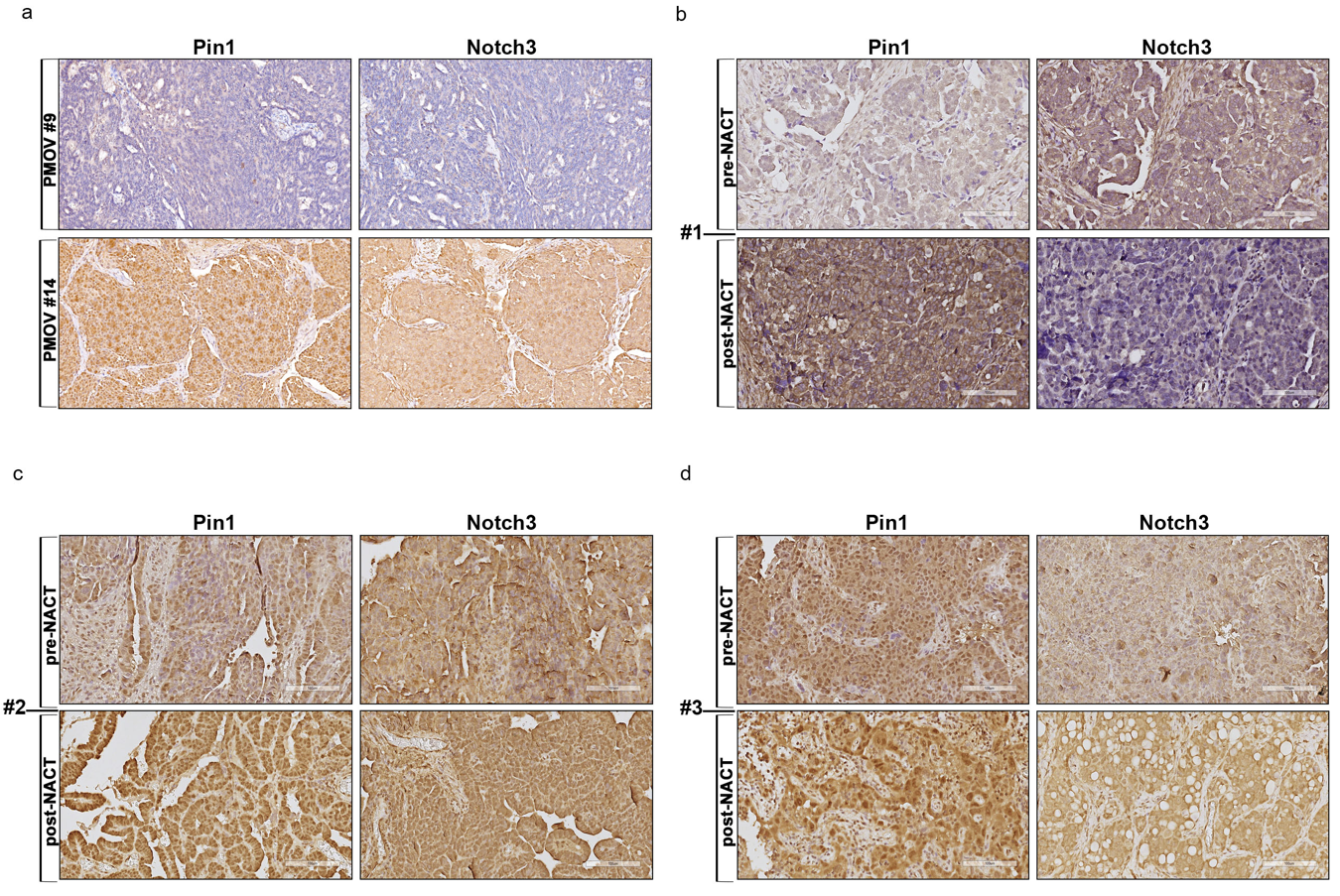
**

**Figure S15. Full resolution of IHC staining.**

**a.** Full resolution of the representative IHC images of the HGSOC biopsies used for the isolation of PMOV#9 and PMOV#14 reported in Fig. 9a (scale bar = 25μM; original magnification, 20X). **b-d.** Full resolution of the representative IHC images of the pre- and post-NACT patients [#1 (**b**), #2 (**c**), #3 (**d**)] reported in Fig. 9e (scale bar = 100μM; original magnification, 20X).

**Supplementary Tables**

**Table S1. HGSOC patients’ clinical data.**

| **Name** | **Stage** | **Protein expression** | | **Mutational status** | | **Exposures (at the time of the IHC)** |
| --- | --- | --- | --- | --- | --- | --- |
|  |  | **WT1** | **PAX8** | **P53** | **BRCA1/2** |  |
| **PMOV#9** | 2A | pos | pos | mut | wt | none |
| **PMOV#14** | 2A | pos | pos | mut | wt | none |
| **Patient#1** | 4A | pos | pos | mut | wt | pre- and post-NACT |
| **Patient#2** | 3C | pos | pos | mut | wt | pre- and post-NACT |
| **Patient#3** | 3C | pos | pos | mut | wt | pre- and post-NACT |

Table summarizing the clinical characteristics of the patients analysed: **1.** stage; **2.** expression of the OC markers WT1 and PAX8; **3.** mutational status of the P53 and BRCA proteins; and **4.** exposures [at the time of immunohistochemistry (IHC) analyses]. Pos=positive; neg=negative; mut=mutated; wt=wild-type; NACT=neoadjuvant therapy.

**Table S2. List of antibodies.**

| **Target / Clone** | **Host** | **Supplier** | **Catalog No.** | **Application(s)** | **Dilution** | **Validation Information**  **(Refs)** |
| --- | --- | --- | --- | --- | --- | --- |
| **Pin1 (G-8)** | Mouse | Santa Cruz Biotechnology | sc-46660 | WB and  IHC | WB (1:2000)  IHC (1:100) | (3) |
| **Vinculin (H-10)** | Mouse | Santa Cruz Biotechnology | sc-25336 | WB | 1:6000 | (4) |
| **GFP (B-2)** | Mouse | Santa Cruz Biotechnology | sc-9996 | WB | 1:1000 | (5) |
| **MPM2** | Mouse | Millipore | 05-368 | WB | 1:500 | (6) |
| **HA-Tag (F-7)** | Mouse | Santa Cruz Biotechnology | sc-7392 | WB | 1:2000 | (7) |
| **β-actin (AC-15)** | Mouse | Sigma-Aldrich | A5316 | WB | 1:10.000 | (8) |
| **FLAG M2–HRP (M2)** | Mouse | Sigma-Aldrich | A8592 | WB | 1:1000 | (8) |
| **Notch3** | Rabbit | Cell Signaling Technology | 2889 | WB | 1:1000 | (8) |
| **Notch3** | Rabbit | Abcam | ab23426 | IHC | 1:50 | (9) |
| **GSK-3β (3D10)** | Mouse | Cell Signaling Technology | 9832 | WB | 1:1000 | (10) |
| **Phospho-Histone H2A.X (Ser139) (20E3)** | Rabbit | Cell Signaling Technology | (#9718), | WB and IF | WB (1:1000)  IF (1:200) | (11,12) |
| **RAD51 [EPR4030(3)]** | Rabbit | Abcam | ab133534 | IF | 1:1000 | (13) |
| **Anti-rabbit HRP** | Goat | Jackson ImmunoResearch | JI111-035-003 | WB | 1:10.000 | (14) |
| **Anti-mouse HRP** | Goat | Jackson ImmunoResearch | JI115-035-003 | WB | 1:10.000 | (15) |
| **Anti-rabbit Alexa Fluor 488** | Goat | Invitrogen - Thermo Fisher Scientific | A11008 | IF | 1:400 | (16) |

**Supplementary Methods**

**Plasmids and antibodies**

pcDNA3-FLAGN3ICD(17), pcDNA3-HAGSK-3β (Addgene, Watertown, MA, USA), pcDNA3-HAPin1wt(18), and pcDNA3-HAPin1S67E(18) were used. The plasmid pcDNA3-GFP (Addgene) was used to evaluate the transfection efficiency. Unless otherwise specified, all transfections were performed for 24h.

All the information (catalog numbers, dilutions, and validation information) regarding the antibodies included in this study are provided in the Table S2.

**Immunofluorescence and confocal imaging**

Immunofluorescence staining was performed as described elsewhere(19). Briefly, cells were fixed in PFA 2% for 20 min at RT, permeabilized with 0.2% Triton X-100 for 20 min at RT and stained with anti-RAD51, anti-Phospho-Histone H2A.X (Ser139) (20E3) and the secondary antibody Alexa Fluor 488-conjugated goat-anti-rabbit as indicated in Table S2. Nuclei were counterstained with Hoechst reagent (cat. #33342, Invitrogen by Thermo Fisher Scientific, Waltham, MA, USA). Single plane confocal images of the cell were acquired using a Zeiss LSM 980 confocal microscope with a 63×/1.35NA oil-immersion objective (Zeiss, Oberkochen, Germany). The foci per nucleus analysis was performed using the Foci Analyzer plugin in the Fiji-ImageJ software. Statistical significance was analyzed using GraphPad Prism 8.0.1.

**Colony assays**

SKOV3_luc clones and Caov3, Caov3-r16T and Caov3-r20T cells were seeded at the appropriate density in 6-well plates. After 24 h, the OC cells were treated as indicated in the figure legend. Colonies were visualized as previously described(20). Briefly, colonies were fixed with a mixture of 90% methanol and 10% chloroform at room temperature for 10 min. Then, they were stained with a solution of 0.1% crystal violet (#HT90132; Sigma‒Aldrich, St. Louis, MO, USA) diluted in methanol for 3 min. After staining, the plates were washed with water and left to dry overnight. Finally, the plates were scanned and stored.

**Peptide and protein production and characterization**

Peptides were synthesized following the Fmoc methodology as previously described in the literature(21). Once the peptide syntheses were completed, the amino acid sequences were functionalized with ε-aminocaproic acid (ε-Ahx), used as a linker, and biotin, following the same protocol used for amino acid couplings(21). The peptides were purified via a Jupiter C18 column (300 Å, 5 µm, 150 × 21.2 mm Ea) operating at a flow rate of 15 mL/min with H2O + 0.1% TFA (solvent A) and CH3CN + 0.1% TFA (solvent B) as solvents. The linear gradient used for the elution was 0−70% solvent B for 15 min. The purity and identity of the peptides were assessed via LC‒MS (UltiMate 3000 HPLC-Q Exactive Plus coupled with Orbitrap mass spectrometers, Thermo Fisher Scientific), which uses an Aeris peptide XB-C18 LC column (100 Å, 3.6 µm, 100 × 2.1 mm) operating at a flow rate of 0.2 mL/min with H2O + 0.1% TFA (solvent A) and CH3CN + 0.1% TFA (solvent B) as solvents. The linear gradient used for elution was 0−70% solvent B for 15 min. The purities of all the peptides were greater than 95%. Recombinant human 6xHis-tagged Pin1 (hereafter, Pin1) was produced following the method described in the literature(22). For Co-IP analyses in the presence of increasing amounts of biotin-labelled peptides, P_1m_ and P_1_ peptides were used at final concentrations of 5μM and 10μM, respectively.

**BLI experiments**

An Octet® Red 96® system (ForteBio, Fremont, CA, USA) was used to detect direct interactions between the N-terminally biotin-labelled peptides (P_1_--P_4_ and P_1m_) and the 6xHis-tagged Pin1 protein (hereafter, Pin1) according to standard instructions. The volume of the buffers or samples was 200 µL. The plate speed and temperature were maintained at 600 rpm and 25°C, respectively, throughout the assay. The biosensors were hydrated with 1x kinetic buffer (10 mM phosphate, 150 mM NaCl, 0.05% Tween 20, pH 7.4) for 10 min before the experiments were started. Each biotin-labelled peptide was loaded onto the Octet® high-capacity super streptavidin (SSA) biosensor surface at 2 µM. The association of the Pin1 protein at several concentrations (0–25 µM) with each biosensor-coated peptide was recorded in parallel until equilibrium was reached. The dissociation step was subsequently performed in kinetic buffer. The biosensors were discarded after each measurement. Fitting operations were performed via Octet analysis Studio 12.2.2.26 software, and a 1:1 Langmuir binding model was assumed. The KDs were derived from equilibrium responses via steady-state analysis via Octet analysis Studio 12.2.2.26 software. Similar results were obtained from two independent experiments. The results of the ‘representative’ experiment are presented.

**Pull-down experiments coupled with LC‒MS analyses**

Three hundred microlitres of N-terminal His-tagged Pin1 protein at 0.34 mg/mL (5 nmol) in 50 mM Tris-HCl or 150 mM NaCl, pH 8 (Buffer A), was incubated with 20 μL of pure-tube Ni-NTA agarose (Cube Biotech) and rotated for 30 min at RT. Then, the beads were washed three times with Buffer A. Three hundred microlitres of peptide at ~ 0.02 mg/mL, with 5 nmol for each peptide, was then incubated with the resin and rotated for 1 h at RT. After three washing steps as previously described, the elution of molecules from the resin was performed by adding 100 μL of buffer A with 500 mM imidazole and lyophilization. Prior to the LC‒MS analysis, the dried peptides were dissolved in 100 μL of H2O/0.1% TFA. A volume of 3 μL of solution was loaded onto an LC‒MS instrument (UltiMate 3000 HPLC-Q Exactive Plus coupled with an Orbitrap mass spectrometer, Thermo Fisher Scientific) with an Aeris peptide XB-C18 LC column (100 Å, 3.6 µm, 100 × 2.1 mm), operating at a flow rate of 0.2 mL/min with H2O + 0.1% TFA (solvent A) and CH3CN + 0.1% TFA (solvent B) as solvents. The linear gradient used for elution was 0−70% solvent B for 15 min. Two independent experiments were performed for each peptide. Peptides were identified by extraction of the base peak in the m/z range: [M+H]+ ± 0.1 and [M+2H]2+ ± 0.1, calculated for each peptide. On the other hand, Pin1 elution was evaluated by western blotting with an anti-His antibody (data not shown).

**Differential proteomics**

A bottom-up proteomics workflow was applied to 30 µg of protein whose cysteines were reduced and alkylated with tris(2carboxyethyl) phosphine (TCEP, Sigma‒Aldrich) and iodoacetamide (IAM, Sigma‒Aldrich), respectively. Furthermore, the proteins were precipitated via methanol (VWR chemicals), acetone (Sigma‒Aldrich) and ethanol (Sigma‒Aldrich) (25%, 25% and 50% v/v) solutions at −20°C overnight and centrifuged at 14000 × g for 15 min at 4°C. The protein pellet was suspended in 100 µl of 1 M urea (Sigma‒Aldrich) and 50 mM ammonium bicarbonate (Sigma‒Aldrich), digested with trypsin (Promega, Fitchburg, WI, USA) at a 1:50 ratio of protease to substrate (w/w), and subjected to overnight digestion at 37°C. The next day, formic acid (Sigma‒Aldrich) was added (0.1% final concentration) to block digestion, and 20 µL of the resulting peptide mixture was injected into an Ultimate 3000 UHPLC (Thermo Fisher Scientific) coupled with an Orbitrap Fusion Tribrid mass spectrometer (Thermo Fisher Scientific). Peptides were desalted on a trap column (Acclaim PepMap 100 C18, Thermo Fisher Scientific) and then separated on a 40-cm long silica capillary (MS WIL, ICT 36007508-50-5, Aarle-Rixtel, the Netherlands), packed in-house with a C18, 1.9 µm, 100 A resin (Michrom BioResources, CA, United States). The analytical column was heated at 40 °C in a column oven (Sonation) and attached to a nanospray Flex ion source (Thermo Fisher Scientific). Peptides were separated on the analytical column by running a 180 min gradient of buffer A (95% water, 5% acetonitrile, and 0.1% formic acid) and buffer B (95% acetonitrile, 5% water, and 0.1% formic acid) at a flow rate of 250 nl/min. The chromatographic starting point was 5% buffer B, and after 5 minutes, B was increased to 6%, that after 130 minutes to 32%, that after 25 minutes to 55%, and that after 4 minutes, to 80% B was reached. The mass spectrometer was operated in positive ion mode, and precursor ion scanning was performed in the Orbitrap analyser in the scan range of m/z 350–1550 with 120K resolution. Data-dependent acquisition was performed in top-speed mode (3 s long maximum cycle time): the most intense precursors were selected through a monoisotopic precursor selection (MIPS) filter and with charge >1, quadrupole isolated and fragmented by 30% higher-energy collision dissociation (HCD). Product ion spectra were recorded in an ion trap (ITMS) with a rapid scan rate. Peptide spectra were searched via Proteome Discoverer 2.4 software (Thermo Fisher Scientific) with the Sequest HT search engine against the *Homo sapiens* database from UniProtKB/Swiss-Prot (SwissProt TaxID=9606_and_subtaxonomies, v2017-10-25). Spectral matches were filtered via the Percolator node, which is based on q values, with a 1% false discovery rate (FDR) via a target-decoy approach. Only master proteins were taken into account, and only specific trypsin cleavages with two miscleavages were admitted. Cysteine carbamydomethylation was set as a static modification, whereas N-acetylation at the protein terminus and methionine oxidation were set as variable modifications. The precursor mass tolerance was set to 15 ppm, while the fragment ion match tolerance was set to 0.6 Da. Quantification was based on the precursor ion intensity of unique and razor peptides via the match between runs option, and normalization to the total peptide amount was applied. with Proteome Discoverer 2.4 software. For differential expression analysis, only proteins whose expression was quantified in at least three out of five replicates in at least one group were retained. Statistical analyses were conducted via Perseus software (version 1.6.15)(23). Intensity values were log2 transformed, and missing values were imputed, replacing them from a normal distribution (width 0.3, downshift 1.8). Volcano plots were extracted from Perseus by applying a t test, and a false discovery rate (FDR) < 0.05 was obtained with 250 randomizations and S0=0.1. Differential protein abundance between experimental conditions (r20T *vs* Caov3 parental) was analysed via a two-sample t test: threshold values of FDR q < 0.05 (Benjamini–Hochberg method) and S0=0.1 were applied to identify statistically deregulated proteins.

Enrichment analysis comparing DE proteins in r20T *vs* Caov3 parental with known mechanisms involved in PT-resistance (EMT, Metabolism, Drug Turnover, Apoptosis, and Inflammation) by *Huang and colleagues*(1), was assessed using Fisher’s exact test, and considering as significant the P values after Benjamini–Hochberg (BH) correction for multiple testing lower than 0.05.

**Phosphorylated peptide analysis by LC‒MS/MS**

Gel slices containing ^FLAG^N3_ICD_ that were untreated or treated with ^HA^GSKβ alone or in combination with the CHIR99021 inhibitor were subjected to 45 min of incubation with 10 mM DTT at 56°C, 30 min of incubation with 55 mM iodocetamide at room temperature in the dark, and digestion with 12.5 ng/µL trypsin at 37°C. The peptide mixture was analysed via liquid chromatography‒mass spectrometry (LC‒MS/MS) via an HPLC Ultimate 3000 connected online with an Orbitrap Fusion Tribrid mass spectrometer (Thermo Fisher Scientific).

Peptides were desalted in a trap column (Acclaim PepMap 100 C18, Thermo Fisher Scientific) and then separated on a column self-packed with C18, 5 μm, 100 Å resin (Michrom BioResources, CA, USA) with a 65 min gradient from 6 to 32% buffer B (95% acetonitrile, 0.1% formic acid). A total of 15 min of washing and 10 min of equilibration steps were added to achieve complete 95 min long run. MS data were acquired in the Orbitrap for both full MS and MS/MS at 120K and 30K resolutions, respectively. The HCD collision energy was set at 30% for fragmentation. The acquired data were analysed via Proteome Discoverer 2.4 (Thermo Fisher Scientific) software via the Flag protein-containing database with the following constraints: specific trypsin cleavages, 4 missed cleavages allowed, and 15 ppm and 0.02 Da tolerances for precursor and fragment ions, respectively. Carbamidomethylation is considered a fixed modification, whereas methionine oxidation and serine, threonine or tyrosine phosphorylation are considered dynamic modifications. A maximum delta Cn of 0.05, a peptide match rank equal to 1 and a 95% probability of phosphosite localization were considered. MS/MS spectra were manually inspected.

**MALDI ToFToF analysis**

Selected bands of ^FLAG^N3_ICD_, untreated or treated with ^HA^GSKβ alone or in combination with the CHIR99021 inhibitor, were subjected to trypsin proteolysis. After alkylation, the bands were incubated with a solution of 25 mM ammonium bicarbonate containing 100 ng of trypsin (Trypsin Gold, mass spectrometry grade, Promega) and 0.01% Trypsin Enhancer (ProteaseMAX™ Surfactant, Promega) for 1 h at 50°C. The analyses of the tryptic mixtures were carried out on a MALDI ToFToF platform (ultrafleXtreme, Bruker, Bremen, DE) equipped with a Smartbeam-II laser in reflector and positive modes. Interesting *m/z* values were subjected to tandem mass experiments in LIFT mode to identify and localize phosphorylated aminoacidic residues. All MS/MS spectra were manually examined and automatically analysed via Flex analysis; the identification of threonine and serine residues in the phosphorylated form was carried out via BioTools software against the SwissProt_all organism database, selecting trypsin as the enzyme, up to 1 missed cleavage, 50 ppm and 0.3 Da tolerance for precursor and fragment ions, respectively; carbamidomethylation of cysteines (+57.021 Da) as a fixed modification; and phosphorylation of serine and threonine residues (+79.966 Da) and oxidation of methionine (+15.995 Da) as variable modifications.

**Molecular Docking**

The docking of N3ICD-derived peptides on Pin1 was performed via the local installation of Rosetta FlexPepDock *ab-initio(24)*. As input structures, we used the crystallographic structure of Pin1 in the compact state (PDB ID: 1F8A). Missing coordinates corresponding to the flexible linker (residues 38–51) were built via the Rosetta next-generation kinematic loop modelling algorithm (NGK)(25) to generate a total of 1500 models. The best-scoring model was chosen for molecular docking. For efficient sampling of the peptide backbone, we generate a library of trimer, pentamer and nonamer backbone fragments, which are extracted from solved protein structures in the Protein Data Bank(26), following the procedure described in (24). The library is constructed on the basis of sequence similarity to the query peptide and the secondary structure predicted for the peptide by PSIPRED(27), resulting in 500 fragments from each category of secondary structure type, i.e., α-helix, extended β-strand and coiled-coil loop (with a total of 1500 fragments for a given query peptide).
The initial N3ICD peptides were then positioned manually near (~2.5 Å) the WW domain of Pin1 via Chimera(28). Finally, the PDB file was created in agreement with the specifications of the original FlexPepDock protocol(24). The unbound Pin1 peptide structures were “relaxed” and “prepacked”. The lowest-energy initial structures were selected for subsequent docking, resulting in 50000 models for each Pin1-peptide complex.

The standard Rosetta score function(29,30) was used, and the models were assessed according to their FlexPepDock reweighted score (“*reweighted_sc*”, sum of the *total score*, *interface score* and *peptide score*; where the *total score* is the overall Rosetta energy score for the complex, the *interface score* is the energy of pairwise interactions across the peptide–protein interface, and the *peptide score* is the sum of the Rosetta energy function over the peptide residues). This score was shown to discriminate near-native structures well in previous FlexPepDock modelling studies(31).

The top-scoring 5000 models (10% of the generated binding modes) are clustered via the Rosetta Cluster application, as described in Gray et al.(32), with a cluster radius cut-off of 1.5 Å peptide backbone atom RMSD. From each cluster, a representative model is subsequently selected according to the best *reweighted score*. The clusters are then ranked according to the energy of their representative models, and the first ranked model is used for MD simulations.

**Molecular dynamics simulations**

MD simulations were conducted to refine the Pin1-peptide complex structures.
All-atom MD simulations in explicit solvents were carried out in GROMACS version 2022.4 via the CHARMM36 force field and TIP3P water model(33). The simulations were set up via the CHARMM-GUI solution builder(34).

Each system was minimized via steepest descent for 50000 steps and a conjugate gradient for another 50000 steps. After minimization, the system was heated from 0 to 310 K in a 1 ns simulation by applying 1 kcal/(mol Å2) harmonic position restraints to the protein and peptide heavy atoms with a constant number, volume and temperature (NVT) ensemble. The system was then equilibrated using a constant number, pressure and temperature (NPT) ensemble at 1 atm and 310 K for 1 ns with the same restraints as in the NVT run. The system was further equilibrated in five successive steps with an iteratively decreasing force constant for the positional restraints for a total equilibration time of 5 ns and starting from randomly assigned velocities and an integration time step of 2 fs. Temperature coupling at 310 K was achieved via a V-rescale thermostat(35), whereas pressure coupling was achieved via a C-rescale barostat(36).

For production runs, without positional restraints, random velocities based on the Boltzmann distribution were assigned. An integration time step of 4 fs was allowed by the hydrogen mass repartitioning (HMR) scheme during production runs(37), with coordinates output every 10 ps. The LINCS algorithm was used to constrain H-bonds.

For each Pin1-peptide complex, 5 replicas of 600 ns each were performed (3 μs of sampling for each complex). Hydrogen bonds were constrained via linear constraints solver (LINCS)(38), and long-range electrostatic interactions were computed via Particle‒Mesh Ewald (PME)(39) with a 1.2 nm cut-off.
Before analysis, MD simulation trajectories were aligned on the Cα atoms of the PPIase domain of Pin1 α-helices via MDAnalysis(40).

Analysis was conducted via the GROMACS built-in analysis tool (*gmx rms, gmx rmsf, gmx covar, gmx anaeig*), MDAnalysis and MDTraj(41). Clustering in the PCA subspace was performed via the implementation of the density peak algorithm by Laio and Rodriguez(42), as implemented in (43).

**Supplementary references**

1. Huang D, Savage SR, Calinawan AP, Lin C, Zhang B, Wang P, et al. A highly annotated database of genes associated with platinum resistance in cancer. Oncogene. 2021 Nov 18;40(46):6395–405.

2. Beatus P, Lundkvist J, Öberg C, Pedersen K, Lendahl U. The origin of the ankyrin repeat region in Notch intracellular domains is critical for regulation of HES promoter activity. Mech Dev. 2001 Jun;104(1–2):3–20.

3. Rustighi A, Zannini A, Tiberi L, Sommaggio R, Piazza S, Sorrentino G, et al. Prolyl‐isomerase Pin1 controls normal and cancer stem cells of the breast. EMBO Mol Med. 2014 Jan 16;6(1):99–119.

4. Giuli MV, Hanieh PN, Forte J, Fabiano MG, Mancusi A, Natiello B, et al. pH-sensitive niosomes for ATRA delivery: A promising approach to inhibit Pin1 in high-grade serous ovarian cancer. Int J Pharm. 2024 Jan;649:123672.

5. Ben-Batalla I, Erdmann R, Jørgensen H, Mitchell R, Ernst T, von Amsberg G, et al. Axl Blockade by BGB324 Inhibits BCR-ABL Tyrosine Kinase Inhibitor-Sensitive and -Resistant Chronic Myeloid Leukemia. Clin Cancer Res. 2017 May 1;23(9):2289–300.

6. Khandani A, Mohtashami M, Camirand A. Inhibitor-2 induced M-phase arrest in Xenopus cycling egg extracts is dependent on MAPK activation. Cell Mol Biol Lett. 2011 Jan 1;16(4).

7. Zhao W, Ouyang C, Huang C, Zhang J, Xiao Q, Zhang F, et al. ELP3 stabilizes c-Myc to promote tumorigenesis. J Mol Cell Biol. 2024 Apr 4;15(9).

8. Franciosa G, Diluvio G, Gaudio F Del, Giuli M V, Palermo R, Grazioli P, et al. Prolyl-isomerase Pin1 controls Notch3 protein expression and regulates T-ALL progression. Oncogene. 2016 Sep 15;35(36):4741–51.

9. Daley-Brown D, Oprea-Iles G, Vann KT, Lanier V, Lee R, Candelaria P V., et al. Type II Endometrial Cancer Overexpresses NILCO: A Preliminary Evaluation. Dis Markers. 2017;2017:1–14.

10. Tao S, Pu Y, Yang EJ, Ren G, Shi C, Chen LJ, et al. Inhibition of GSK3β is synthetic lethal with FHIT loss in lung cancer by blocking homologous recombination repair. Exp Mol Med. 2025 Jan 6;57(1):167–83.

11. Sonego M, Pellizzari I, Dall’Acqua A, Pivetta E, Lorenzon I, Benevol S, et al. Common biological phenotypes characterize the acquisition of platinum-resistance in epithelial ovarian cancer cells. Sci Rep. 2017 Aug 2;7(1):7104.

12. Solier S, Sordet O, Kohn KW, Pommier Y. Death Receptor-Induced Activation of the Chk2- and Histone H2AX-Associated DNA Damage Response Pathways. Mol Cell Biol. 2009 Jan 1;29(1):68–82.

13. Di Giulio S, Colicchia V, Pastorino F, Pedretti F, Fabretti F, Nicolis di Robilant V, et al. A combination of PARP and CHK1 inhibitors efficiently antagonizes MYCN-driven tumors. Oncogene. 2021 Oct 28;40(43):6143–52.

14. Chung CH, Hsu KC, Huang MM, Tu HJ, Pan SL, Chao MW. Discovery of a novel RSK2 inhibitor for the treatment of metastatic pancreatic cancer. J Enzyme Inhib Med Chem. 2025 Dec 31;40(1).

15. Yun S, Jeon C, Park SJ, Lee H, Kwon OK, Lee JW. *N* ‑(p‑Coumaroyl) serotonin mitigates inflammatory responses in lipopolysaccharide‑stimulated RAW264.7 cells and phorbol 12‑myristate 13‑acetate‑stimulated A549 cells through NF‑κB and MAPK inactivation. Exp Ther Med. 2025 Dec 22;31(2):1–10.

16. van Vliet AR, Gillingham AK, Morgan TE, Ohashi Y, Smith TS, Abid Ali F, et al. A Rab1 interactome illuminates a dual role in autophagy and membrane trafficking. Journal of Cell Biology. 2026 Mar 2;225(3).

17. Bellavia D. Constitutive activation of NF-kappaB and T-cell leukemia/lymphoma in Notch3 transgenic mice. EMBO J. 2000 Jul 3;19(13):3337–48.

18. Rustighi A, Tiberi L, Soldano A, Napoli M, Nuciforo P, Rosato A, et al. The prolyl-isomerase Pin1 is a Notch1 target that enhances Notch1 activation in cancer. Nat Cell Biol. 2009 Feb 18;11(2):133–42.

19. Checquolo S, Palermo R, Cialfi S, Ferrara G, Oliviero C, Talora C, et al. Differential subcellular localization regulates c-Cbl E3 ligase activity upon Notch3 protein in T-cell leukemia. Oncogene. 2010 Mar 11;29(10):1463–74.

20. Pelullo M, Zema S, De Carolis M, Cialfi S, Giuli MV, Palermo R, et al. 5FU/Oxaliplatin-Induced Jagged1 Cleavage Counteracts Apoptosis Induction in Colorectal Cancer: A Novel Mechanism of Intrinsic Drug Resistance. Front Oncol. 2022 Jul 1;12.

21. Caporale A, Doti N, Monti A, Sandomenico A, Ruvo M. Automatic procedures for the synthesis of difficult peptides using oxyma as activating reagent: A comparative study on the use of bases and on different deprotection and agitation conditions. Peptides (NY). 2018 Apr;102:38–46.

22. Monti A, Ronca R, Campiani G, Ruvo M, Doti N. Expression, Purification, Structural and Functional Characterization of Recombinant Human Parvulin 17. Mol Biotechnol. 2023 Mar 25;65(3):337–49.

23. Tyanova S, Temu T, Sinitcyn P, Carlson A, Hein MY, Geiger T, et al. The Perseus computational platform for comprehensive analysis of (prote)omics data. Nat Methods. 2016 Sep 27;13(9):731–40.

24. Raveh B, London N, Zimmerman L, Schueler-Furman O. Rosetta FlexPepDock ab-initio: Simultaneous Folding, Docking and Refinement of Peptides onto Their Receptors. PLoS One. 2011 Apr 29;6(4):e18934.

25. Stein A, Kortemme T. Improvements to Robotics-Inspired Conformational Sampling in Rosetta. PLoS One. 2013 May 21;8(5):e63090.

26. Berman HM, Battistuz T, Bhat TN, Bluhm WF, Bourne PE, Burkhardt K, et al. The Protein Data Bank. Acta Crystallogr D Biol Crystallogr. 2002 Jun 1;58(6):899–907.

27. Jones DT. Protein secondary structure prediction based on position-specific scoring matrices 1 1Edited by G. Von Heijne. J Mol Biol. 1999 Sep;292(2):195–202.

28. Pettersen EF, Goddard TD, Huang CC, Couch GS, Greenblatt DM, Meng EC, et al. UCSF Chimera—A visualization system for exploratory research and analysis. J Comput Chem. 2004 Oct;25(13):1605–12.

29. Peters B, Sette A. Generating quantitative models describing the sequence specificity of biological processes with the stabilized matrix method. BMC Bioinformatics. 2005 May 31;6(1):132.

30. Alford RF, Leaver-Fay A, Jeliazkov JR, O’Meara MJ, DiMaio FP, Park H, et al. The Rosetta All-Atom Energy Function for Macromolecular Modeling and Design. J Chem Theory Comput. 2017 Jun 13;13(6):3031–48.

31. Alam N, Goldstein O, Xia B, Porter KA, Kozakov D, Schueler-Furman O. High-resolution global peptide-protein docking using fragments-based PIPER-FlexPepDock. PLoS Comput Biol. 2017 Dec 27;13(12):e1005905.

32. Gray JJ, Moughon S, Wang C, Schueler-Furman O, Kuhlman B, Rohl CA, et al. Protein–Protein Docking with Simultaneous Optimization of Rigid-body Displacement and Side-chain Conformations. J Mol Biol. 2003 Aug;331(1):281–99.

33. Abraham MJ, Murtola T, Schulz R, Páll S, Smith JC, Hess B, et al. GROMACS: High performance molecular simulations through multi-level parallelism from laptops to supercomputers. SoftwareX. 2015 Sep;1–2:19–25.

34. Jo S, Kim T, Iyer VG, Im W. CHARMM‐GUI: A web‐based graphical user interface for CHARMM. J Comput Chem. 2008 Aug 10;29(11):1859–65.

35. Bussi G, Donadio D, Parrinello M. Canonical sampling through velocity rescaling. J Chem Phys. 2007 Jan 7;126(1).

36. Bernetti M, Bussi G. Pressure control using stochastic cell rescaling. J Chem Phys. 2020 Sep 21;153(11).

37. Hopkins CW, Le Grand S, Walker RC, Roitberg AE. Long-Time-Step Molecular Dynamics through Hydrogen Mass Repartitioning. J Chem Theory Comput. 2015 Apr 14;11(4):1864–74.

38. Hess B, Bekker H, Berendsen HJC, Fraaije JGEM. LINCS: A linear constraint solver for molecular simulations. J Comput Chem. 1997 Sep;18(12):1463–72.

39. Abraham MJ, Gready JE. Optimization of parameters for molecular dynamics simulation using smooth particle‐mesh Ewald in GROMACS 4.5. J Comput Chem. 2011 Jul 15;32(9):2031–40.

40. Michaud‐Agrawal N, Denning EJ, Woolf TB, Beckstein O. MDAnalysis: A toolkit for the analysis of molecular dynamics simulations. J Comput Chem. 2011 Jul 30;32(10):2319–27.

41. McGibbon RT, Beauchamp KA, Harrigan MP, Klein C, Swails JM, Hernández CX, et al. MDTraj: A Modern Open Library for the Analysis of Molecular Dynamics Trajectories. Biophys J. 2015 Oct;109(8):1528–32.

42. Rodriguez A, Laio A. Clustering by fast search and find of density peaks. Science (1979). 2014 Jun 27;344(6191):1492–6.

43. Träger S, Tamò G, Aydin D, Fonti G, Audagnotto M, Dal Peraro M. CLoNe: automated clustering based on local density neighborhoods for application to biomolecular structural ensembles. Bioinformatics. 2021 May 17;37(7):921–8.
