# Supplementary material for: Prolyl-isomerase Pin1 drives platinum resistance by regulating Notch3 stability and function in ovarian cancer
Source: J Exp Clin Cancer Res. 2026 Feb 11;45:71. doi: 10.1186/s13046-026-03658-x (PMC12998371; doi:10.1186/s13046-026-03658-x)
Supplement: Supplementary file 2 — Supplementary Material 2. [file 13046_2026_3658_MOESM2_ESM.pdf]

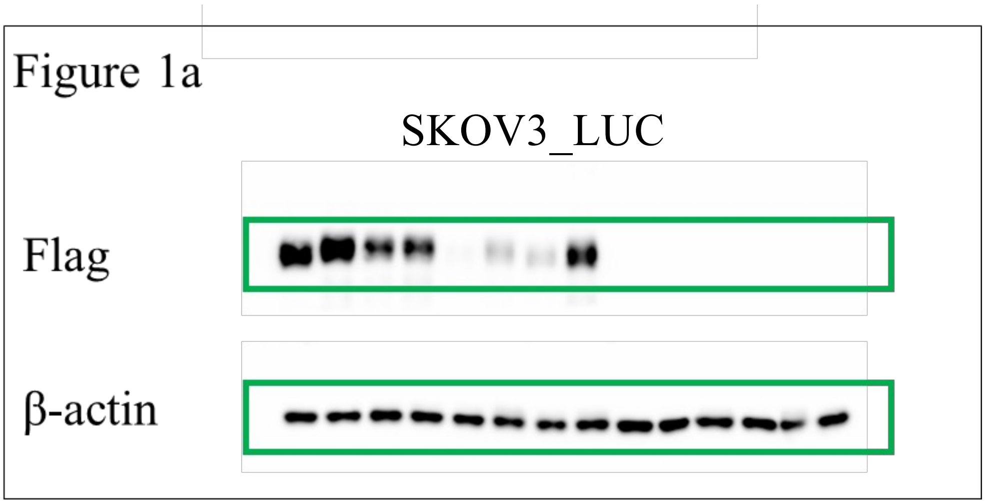

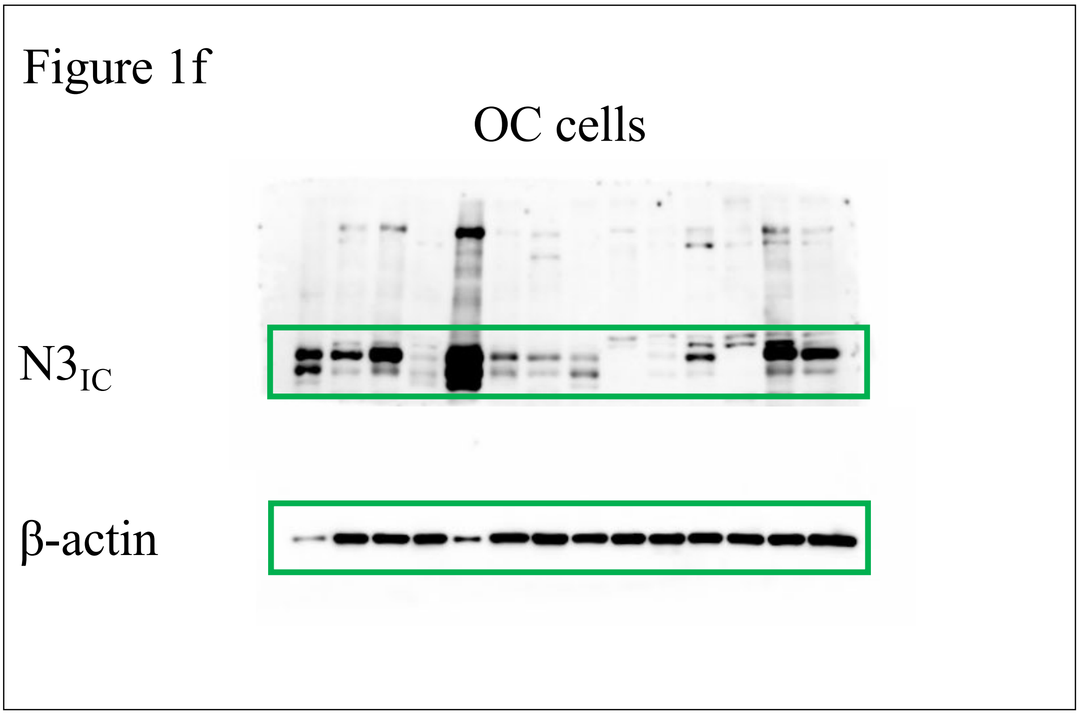

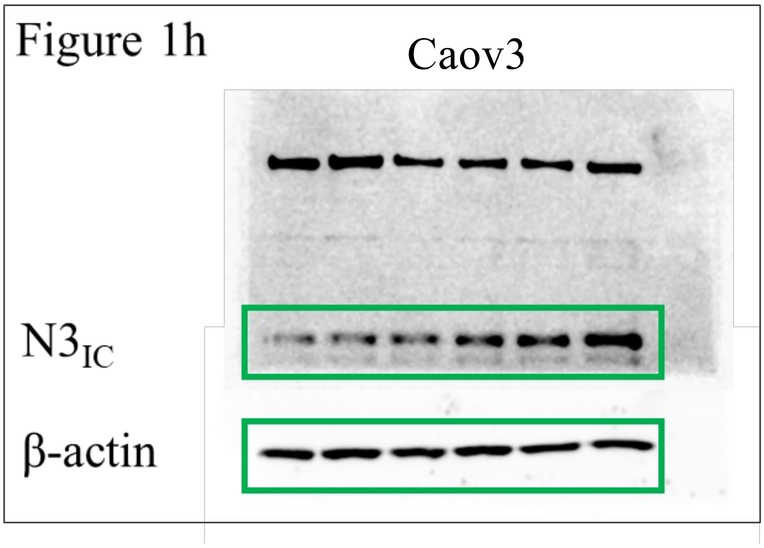

Supplementary Figure S16. Uncropped blots and gels for the main Figure 1

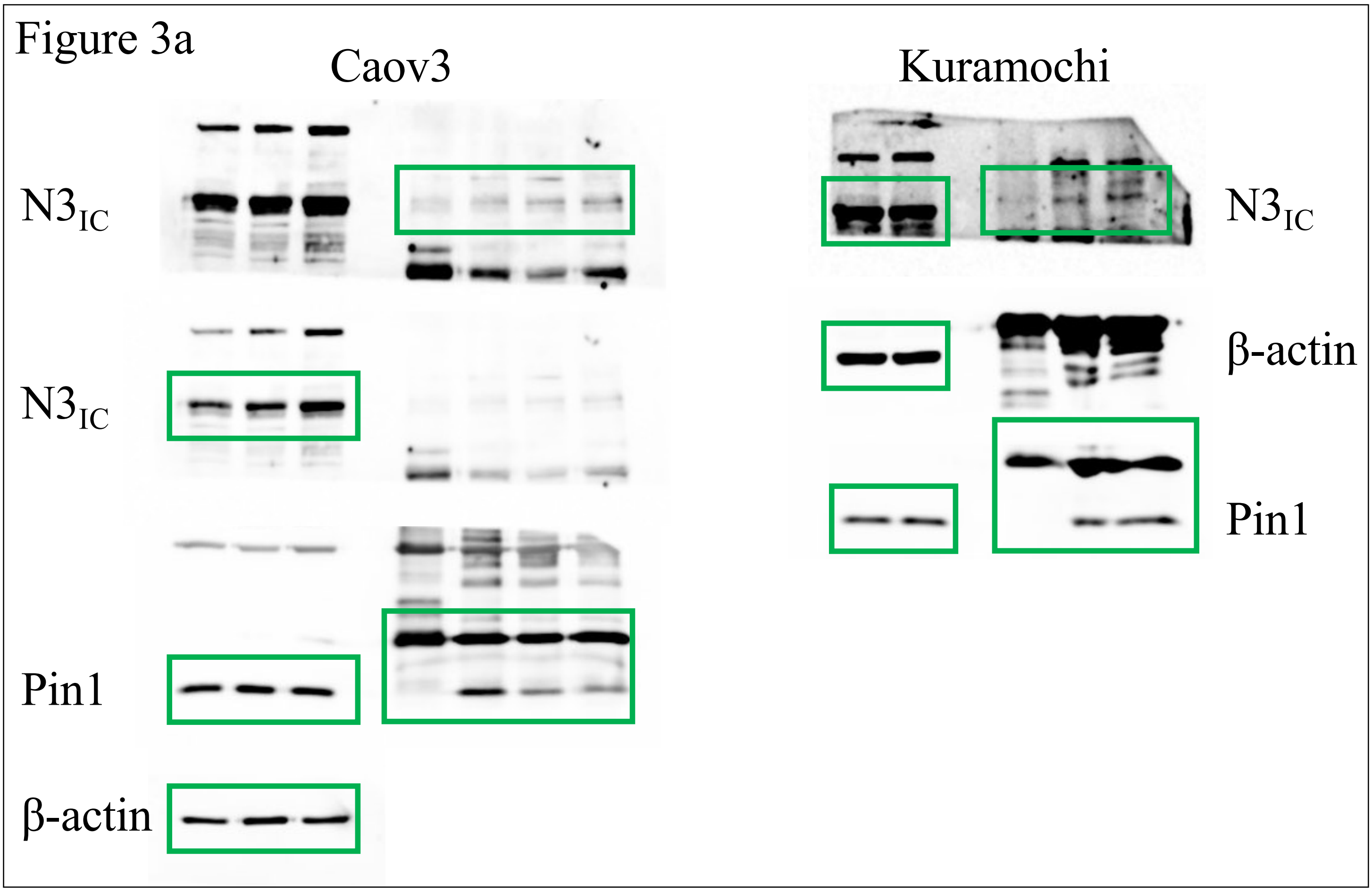

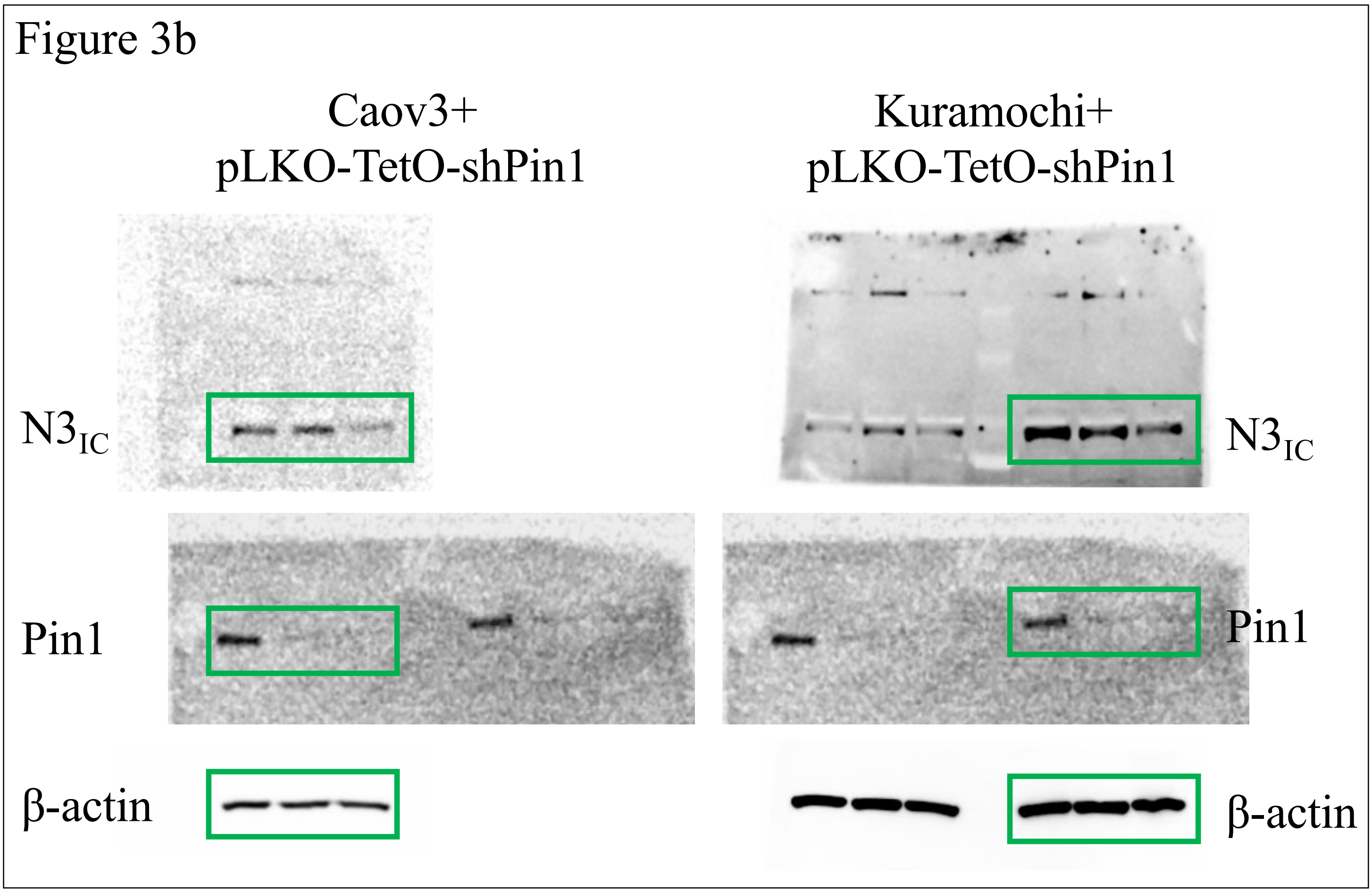

Supplementary Figure S17a. Uncropped blots and gels for the main Figure 3

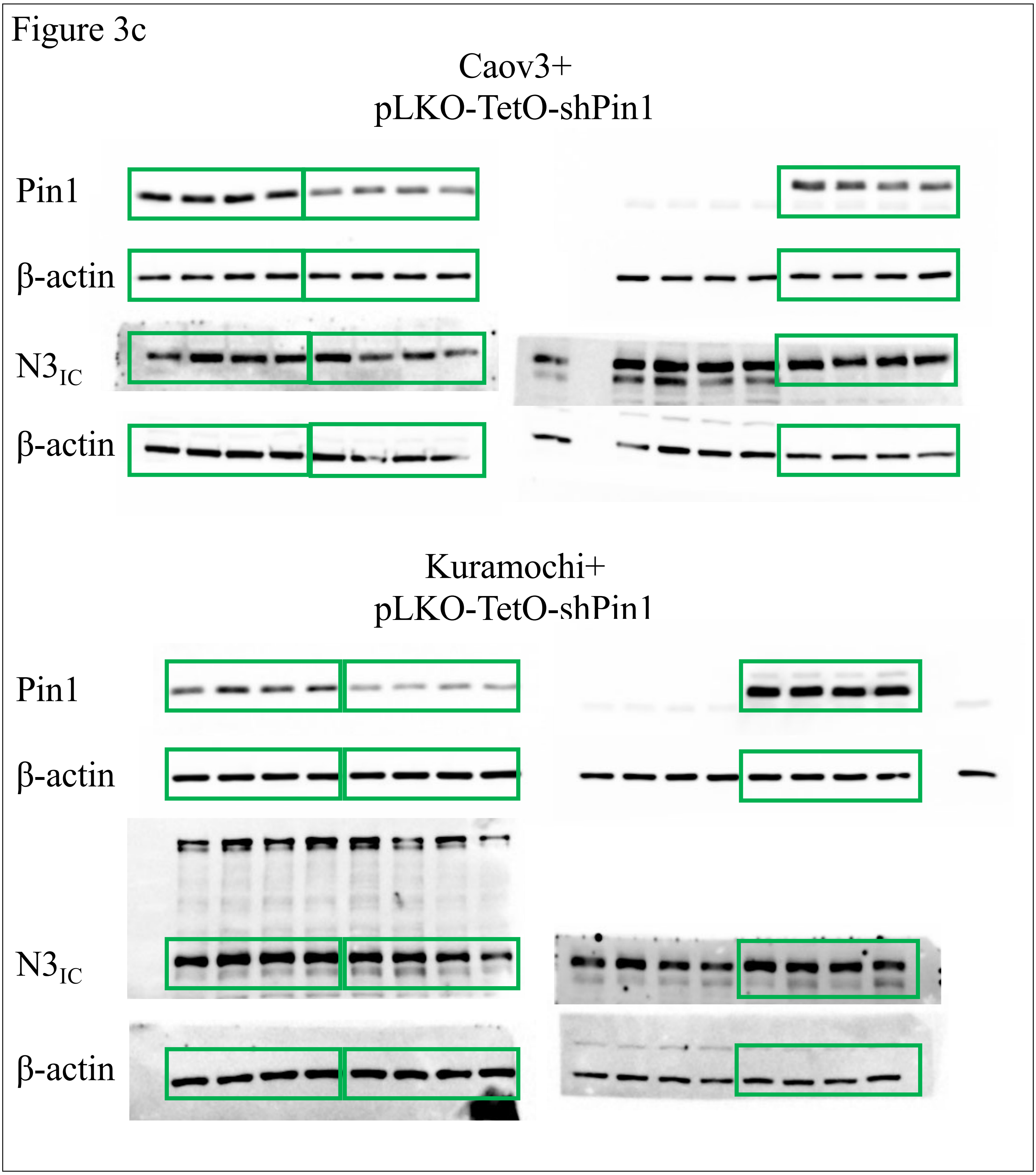

Supplementary Figure S17b. Uncropped blots and gels for the main Figure 3

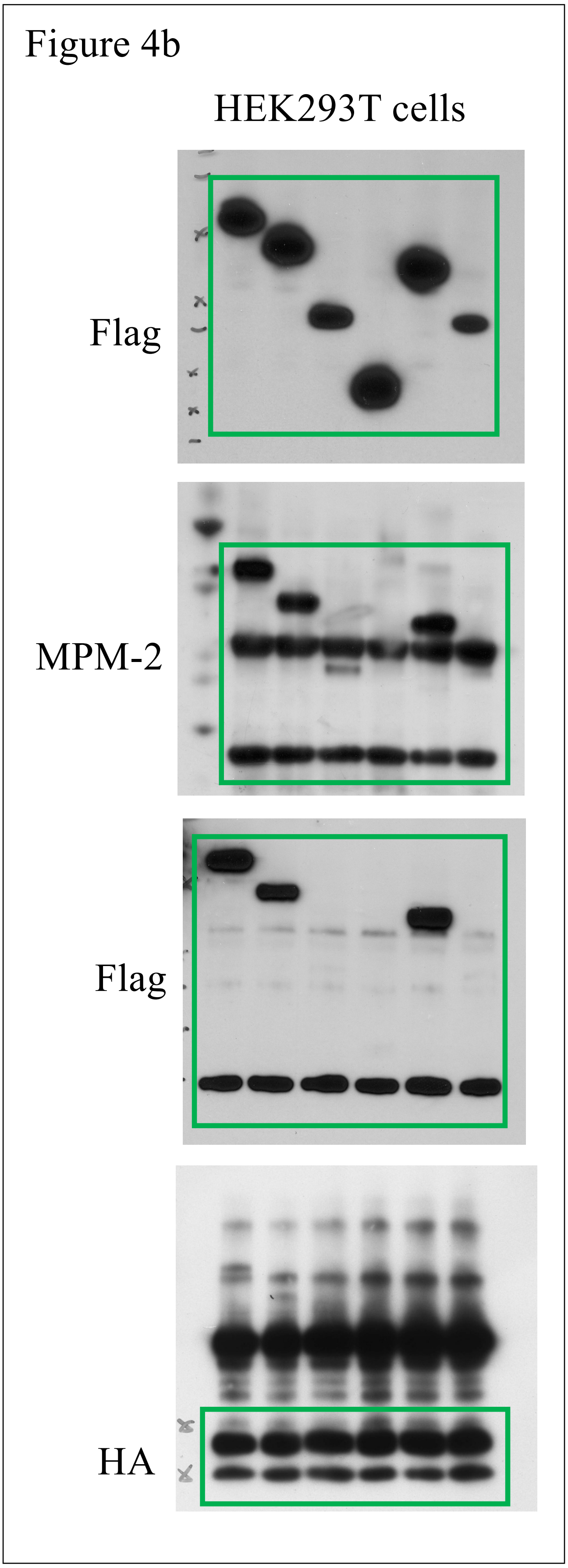

Supplementary Figure S18. Uncropped blots and gels for the main Figure 4

Figure 5g

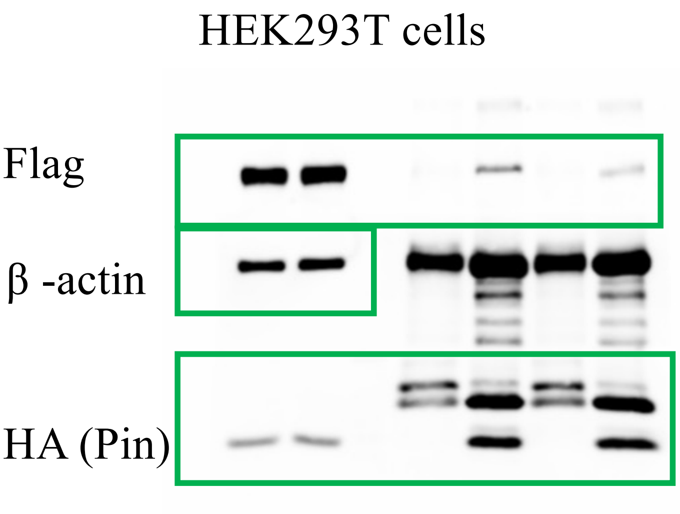

Supplementary Figure S19. Uncropped blots and gels for the main Figure 5

Figure 6e

HEK293T cells

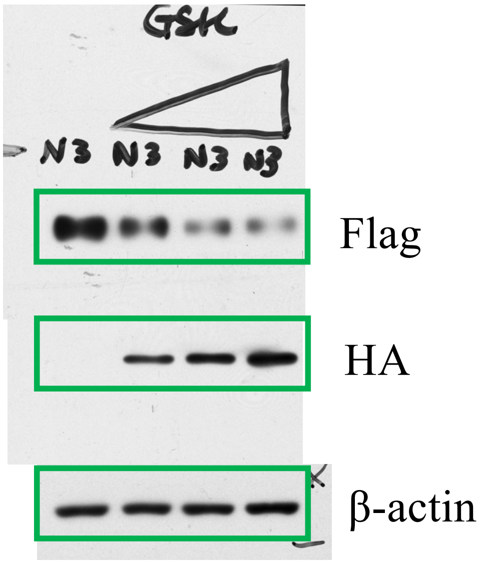

Figure 6f

HEK293T cells

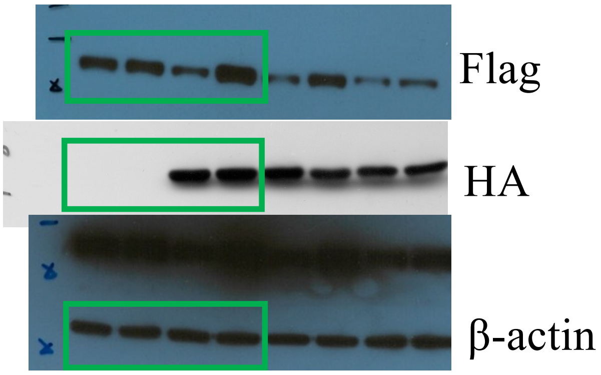

Supplementary Figure S20a. Uncropped blots and gels for the main Figure 6

Figure 6g

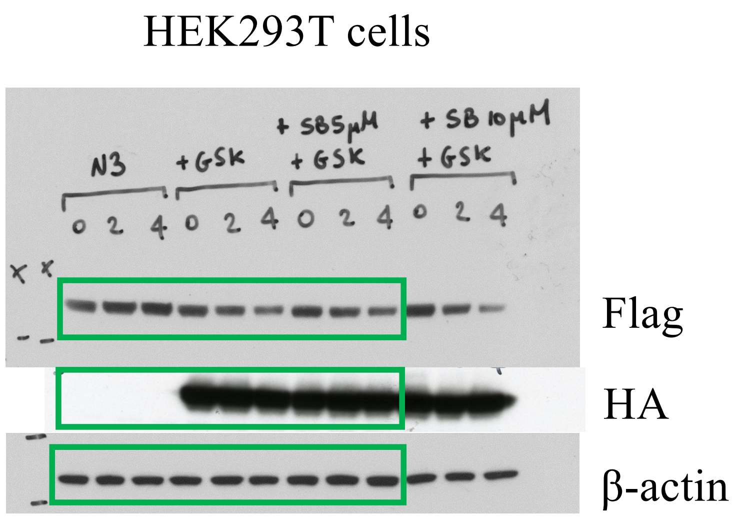

Figure 6h

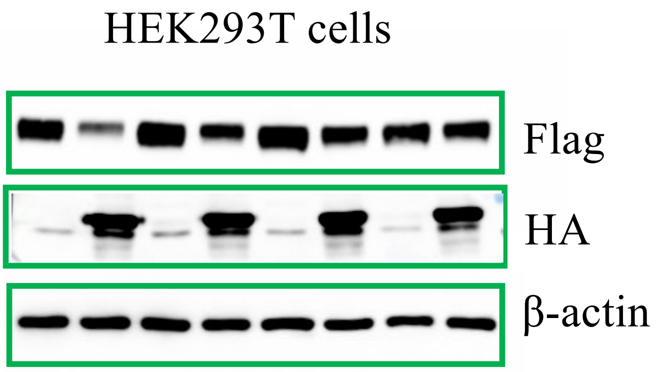

Figure 6i

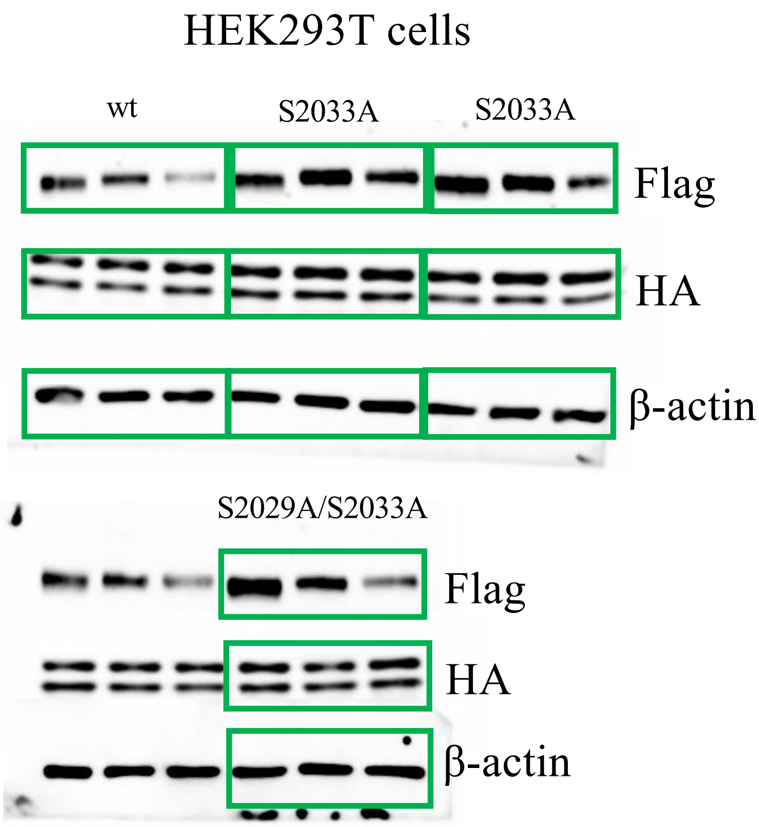

Supplementary Figure S20b. Uncropped blots and gels for the main Figure 6

Figure 7a

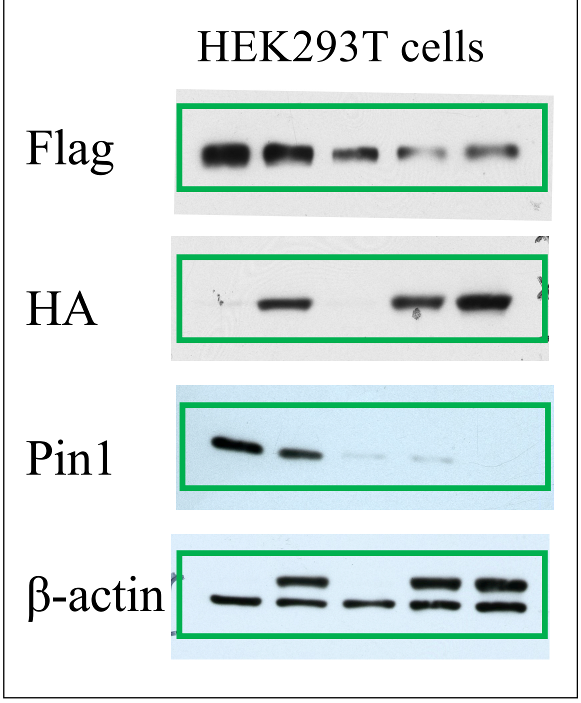

Figure 7b

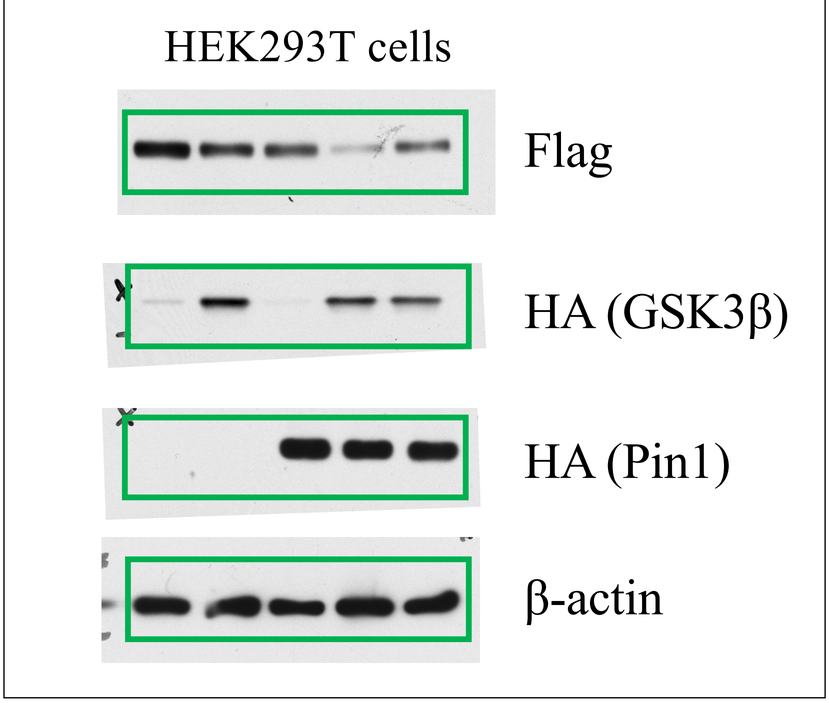

Figure 7c

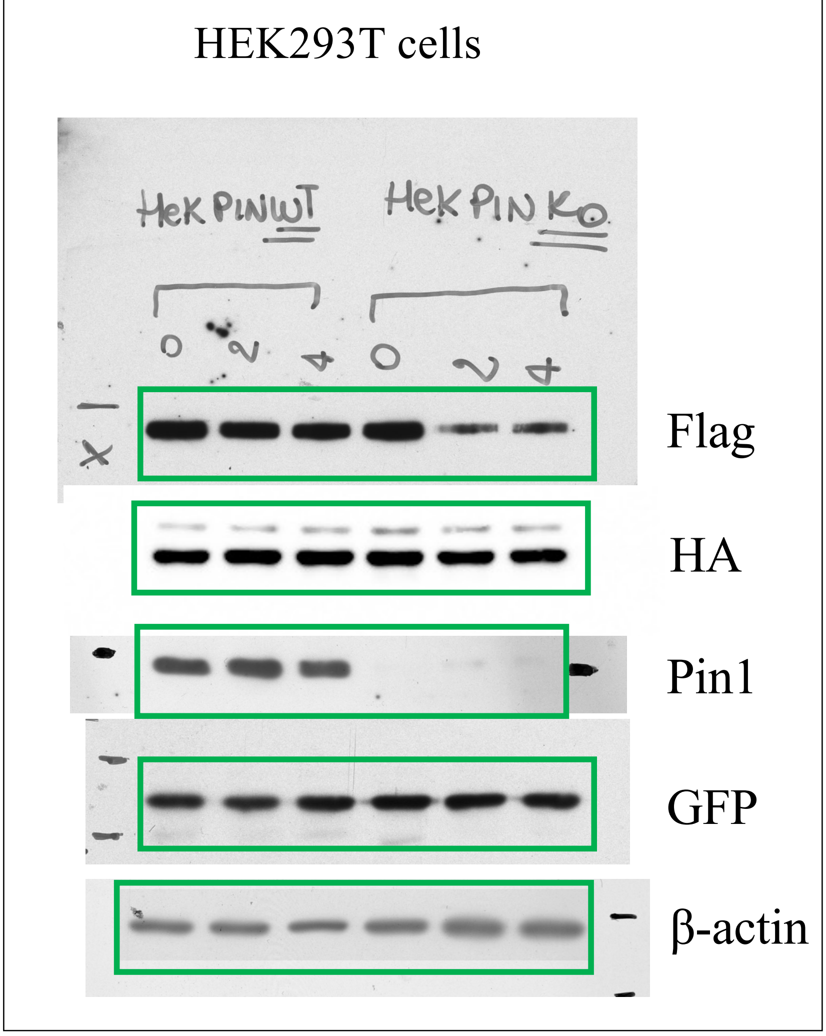

Supplementary Figure S21a. Uncropped blots and gels for the main Figure 7

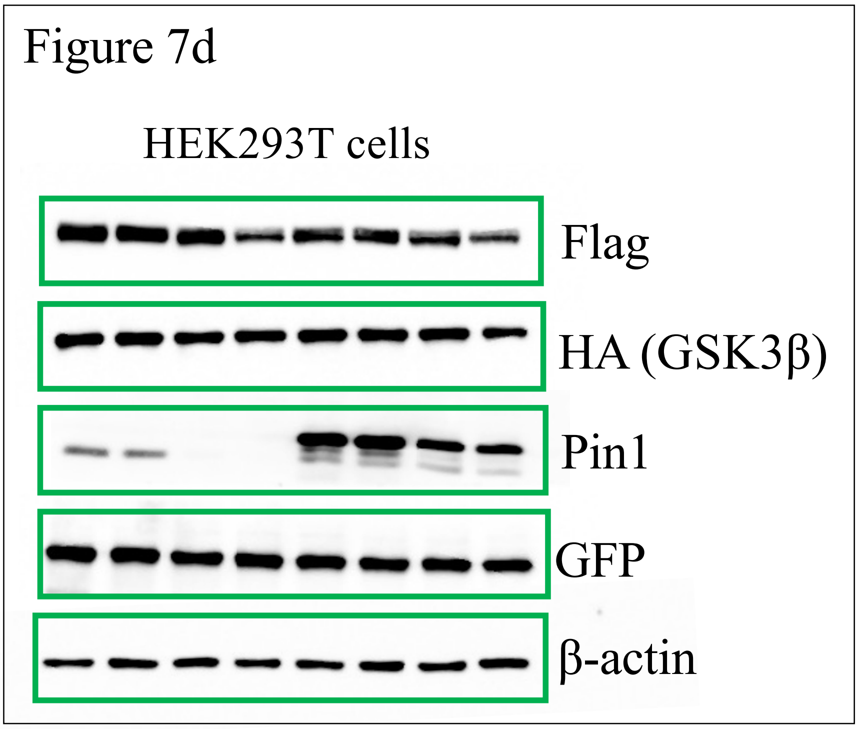

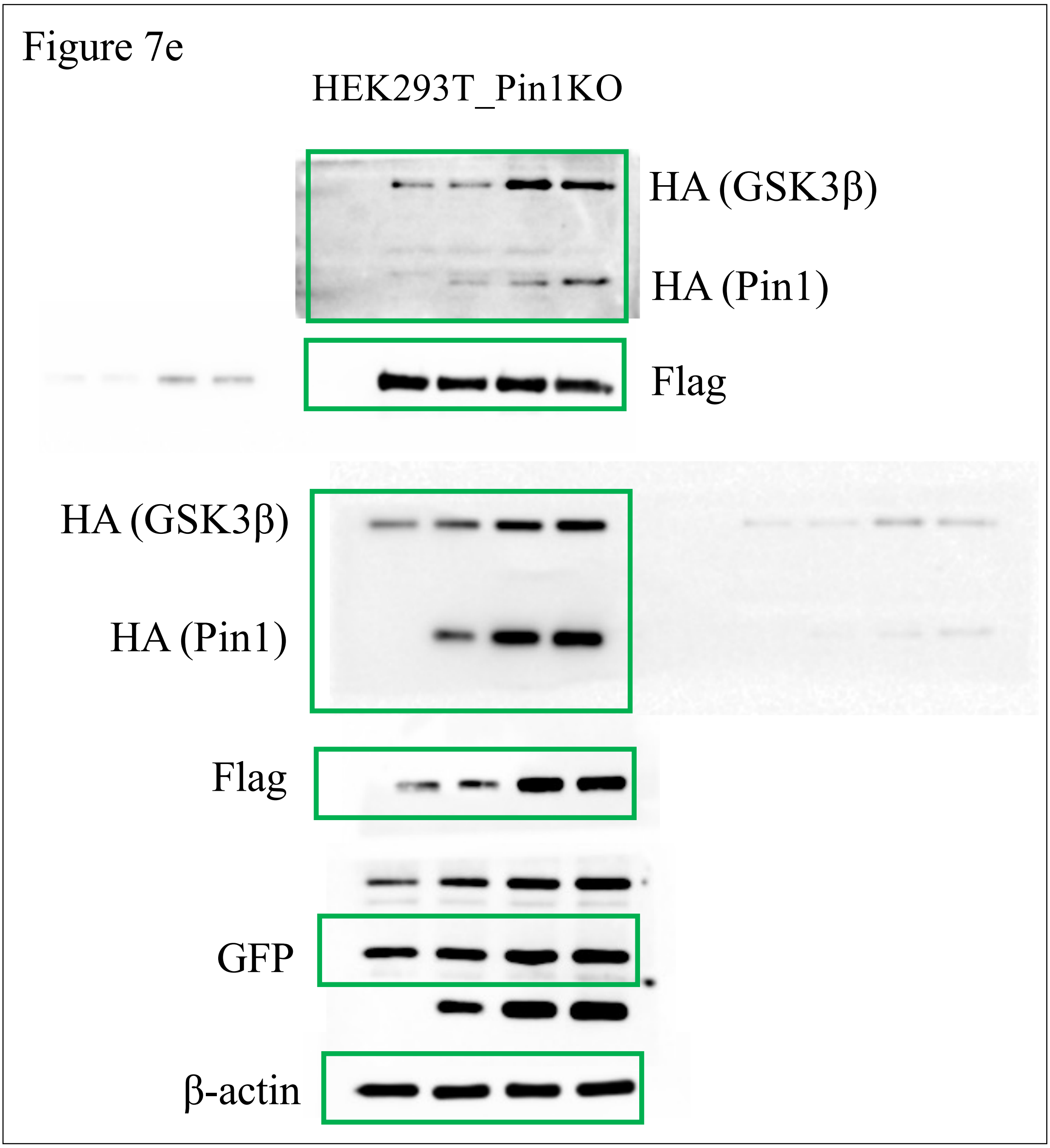

Supplementary Figure S21b. Uncropped blots and gels for the main Figure 7

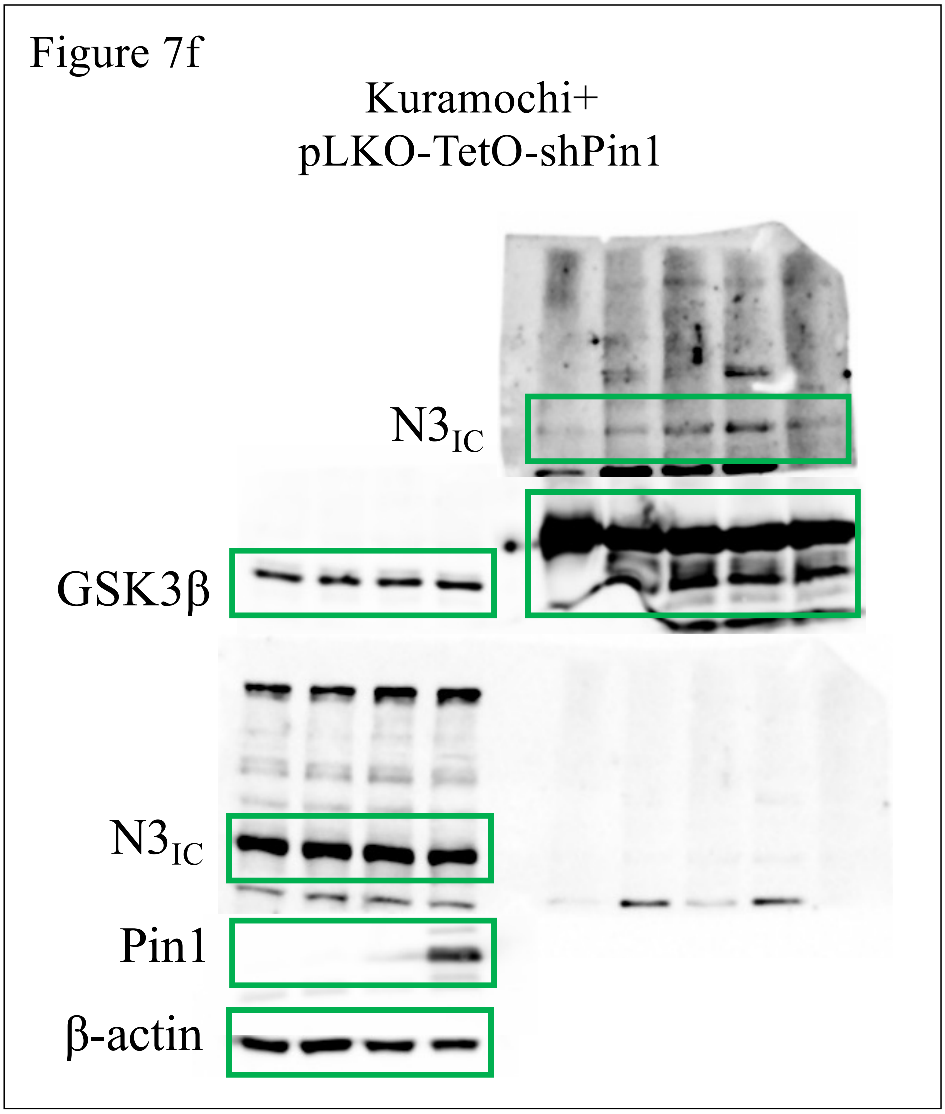

Supplementary Figure S21c. Uncropped blots and gels for the main Figure 7

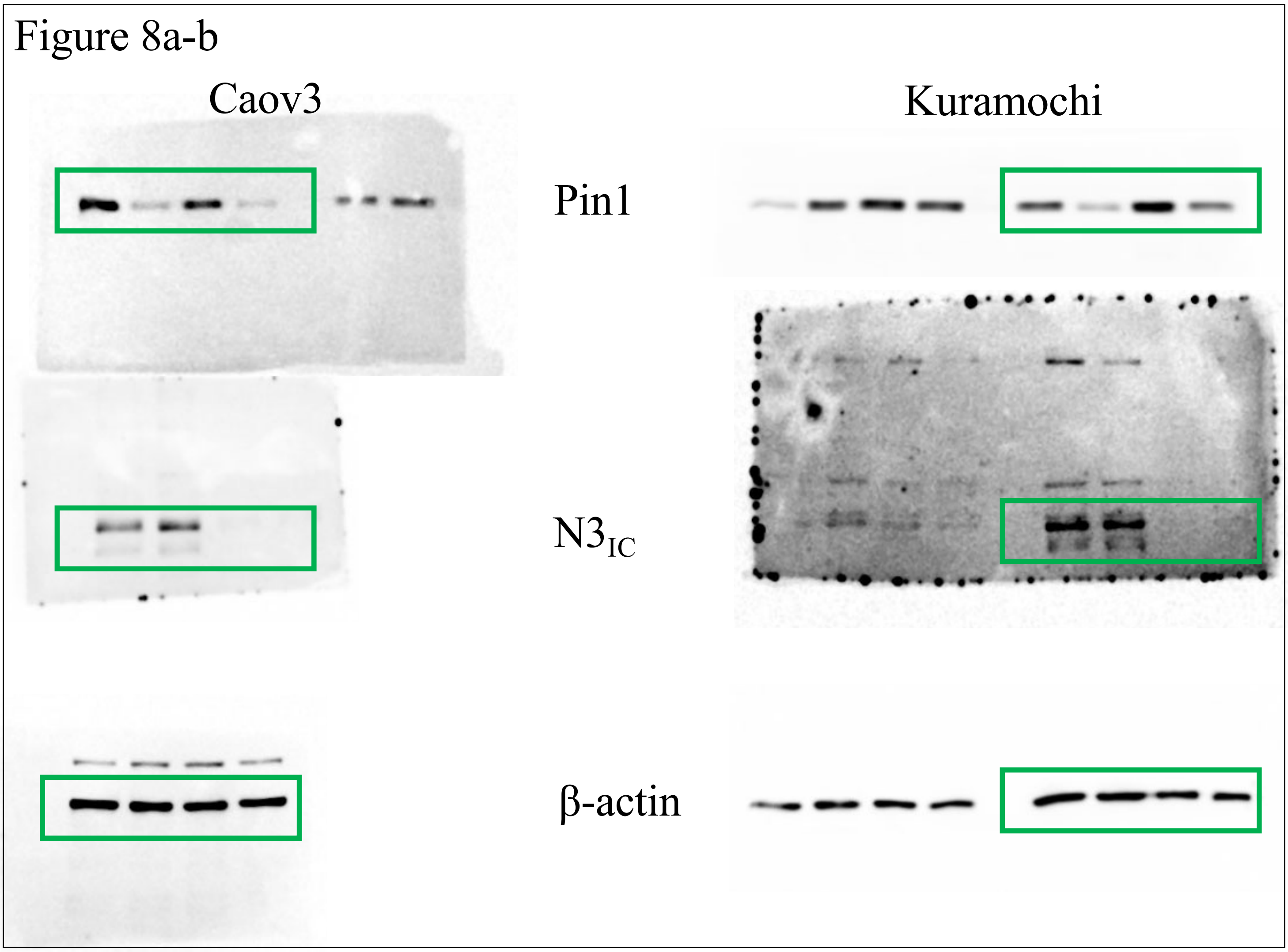

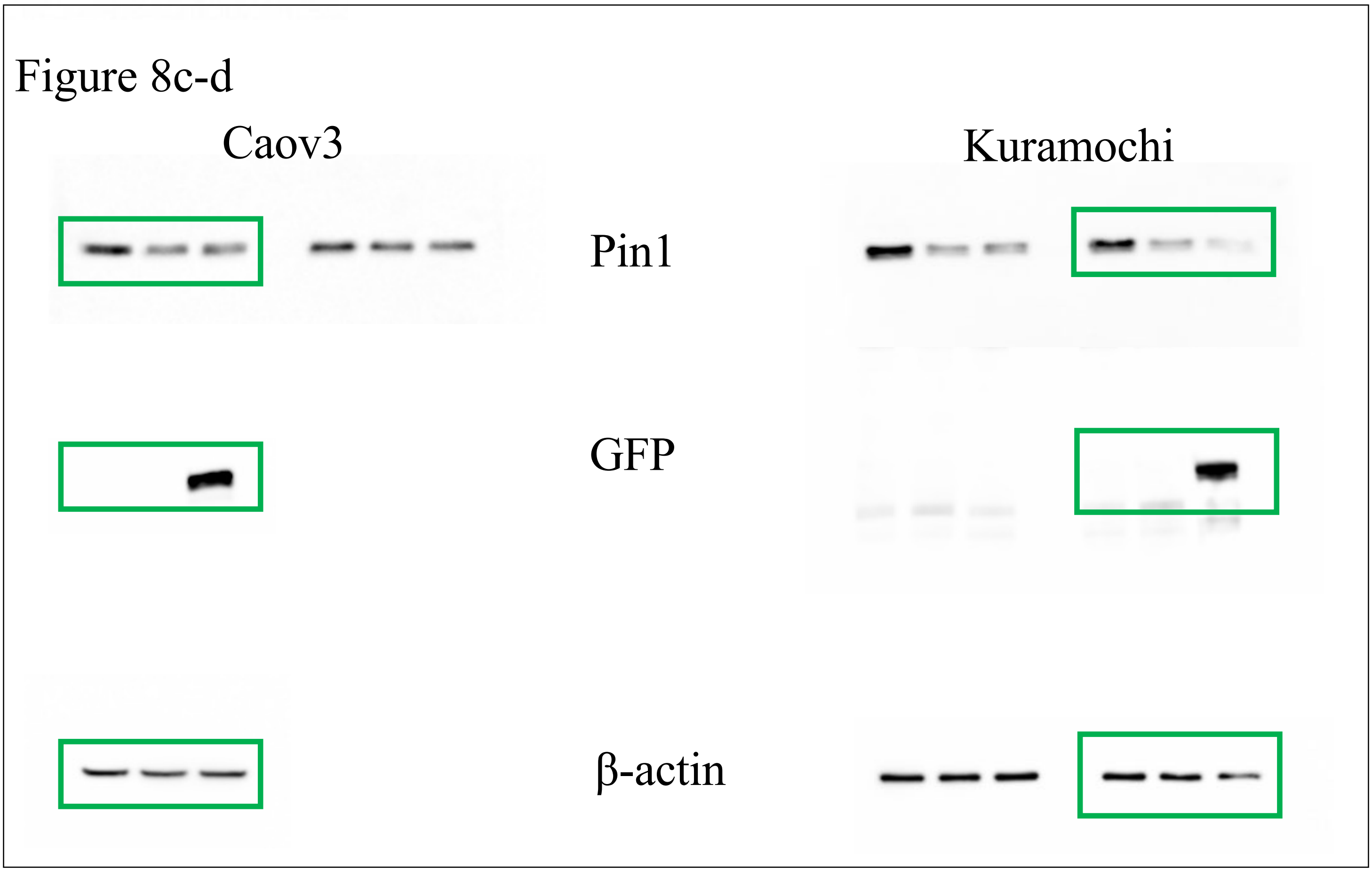

Supplementary Figure S22. Uncropped blots and gels for the main Figure 8

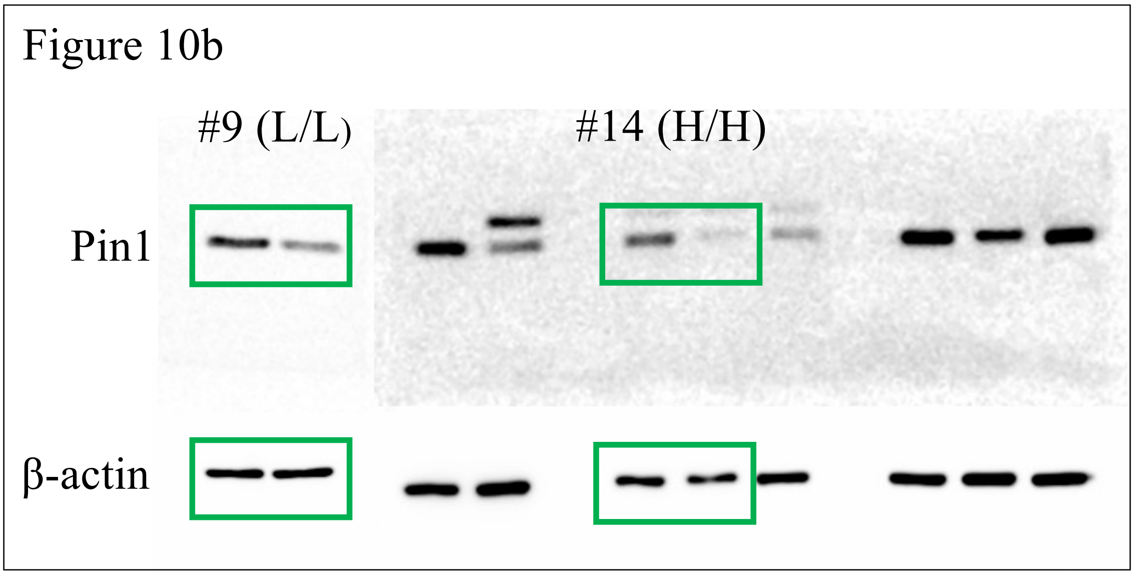

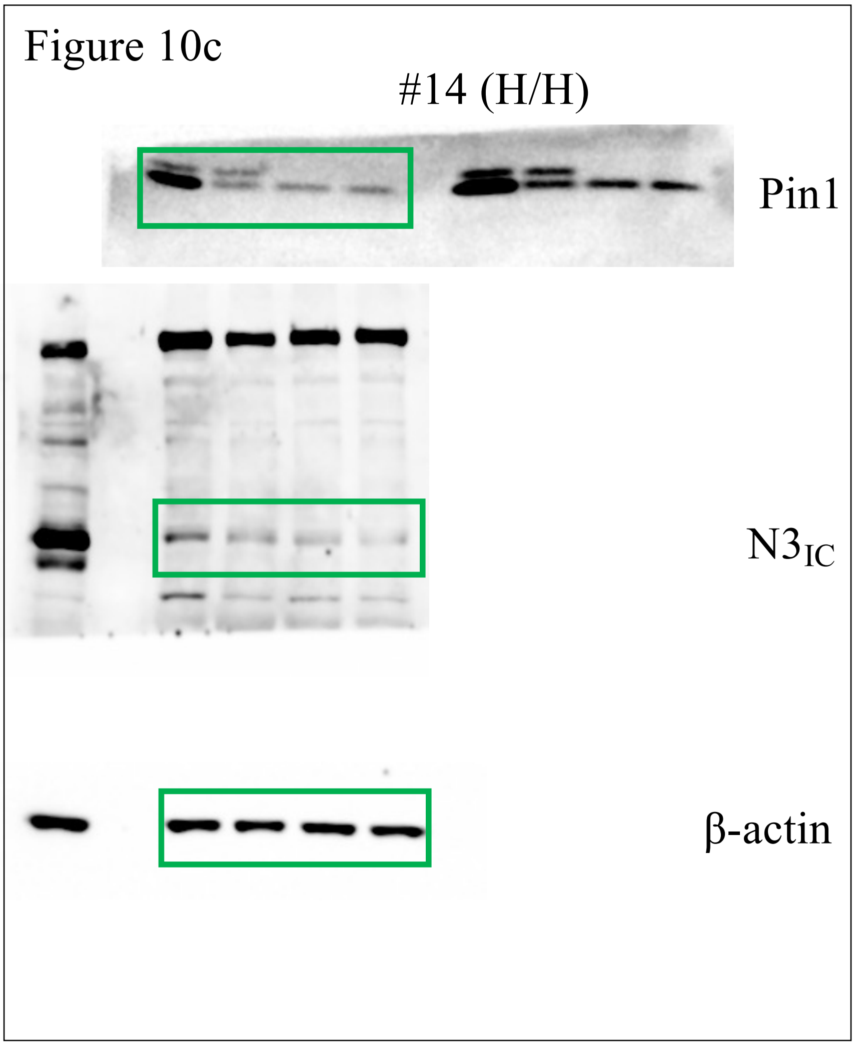

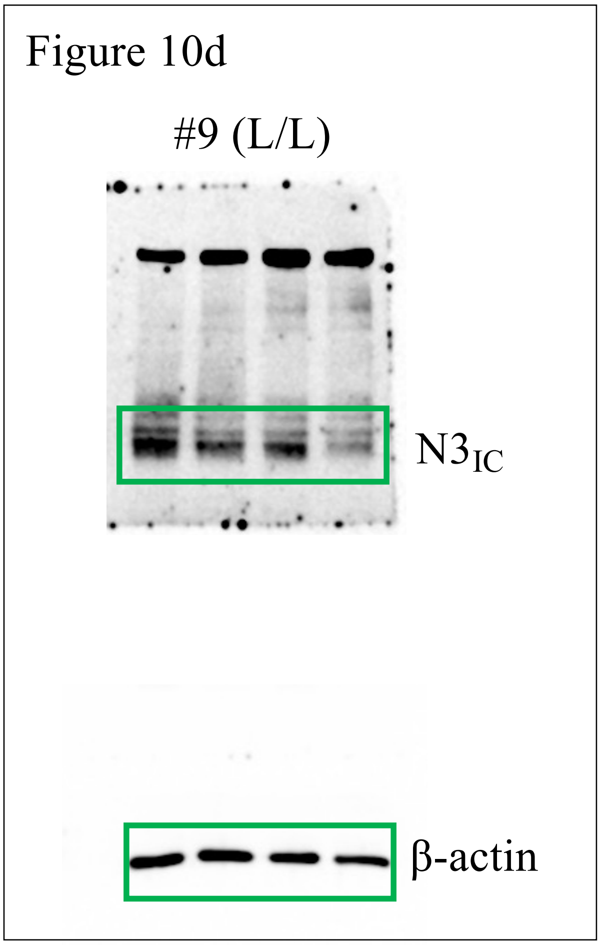

Supplementary Figure S23. Uncropped blots and gels for the main Figure 10

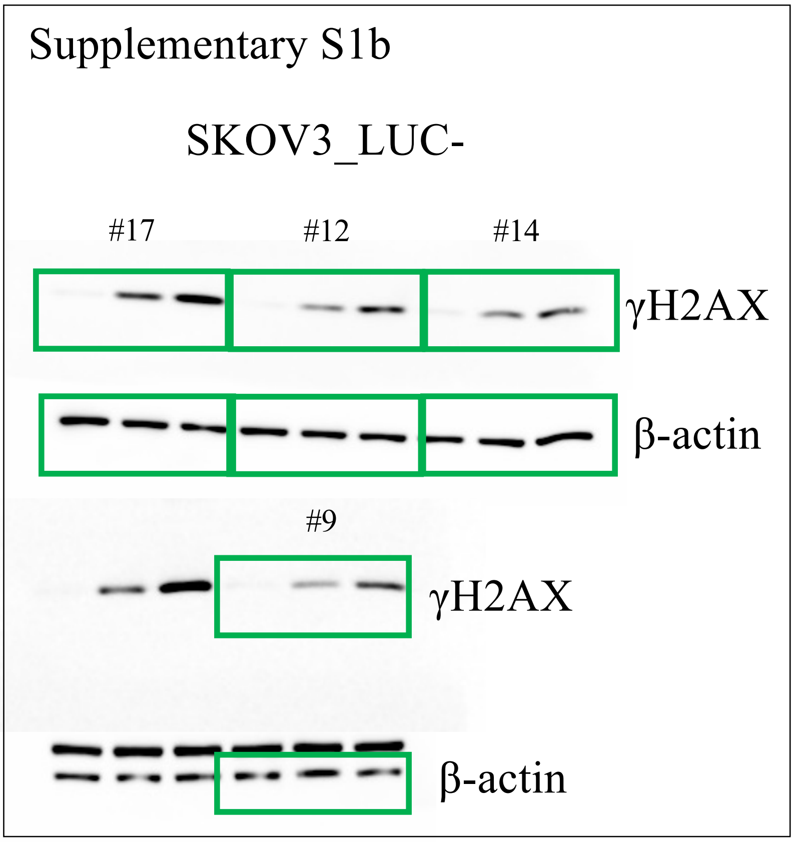

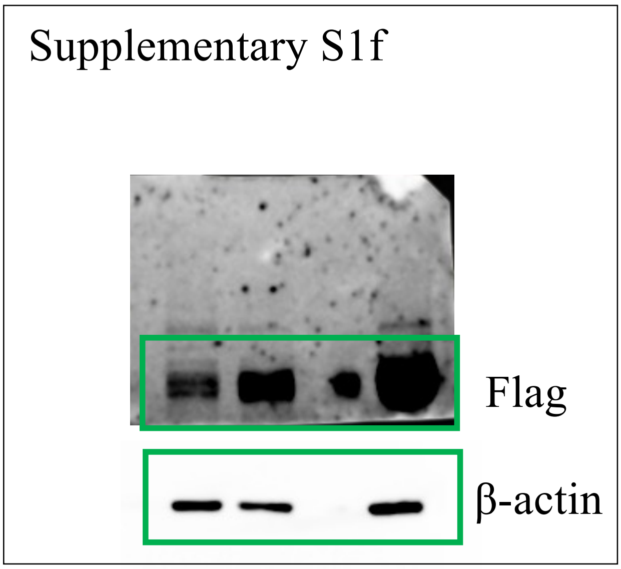

Supplementary Figure S24. Uncropped blots and gels for the Figure S1

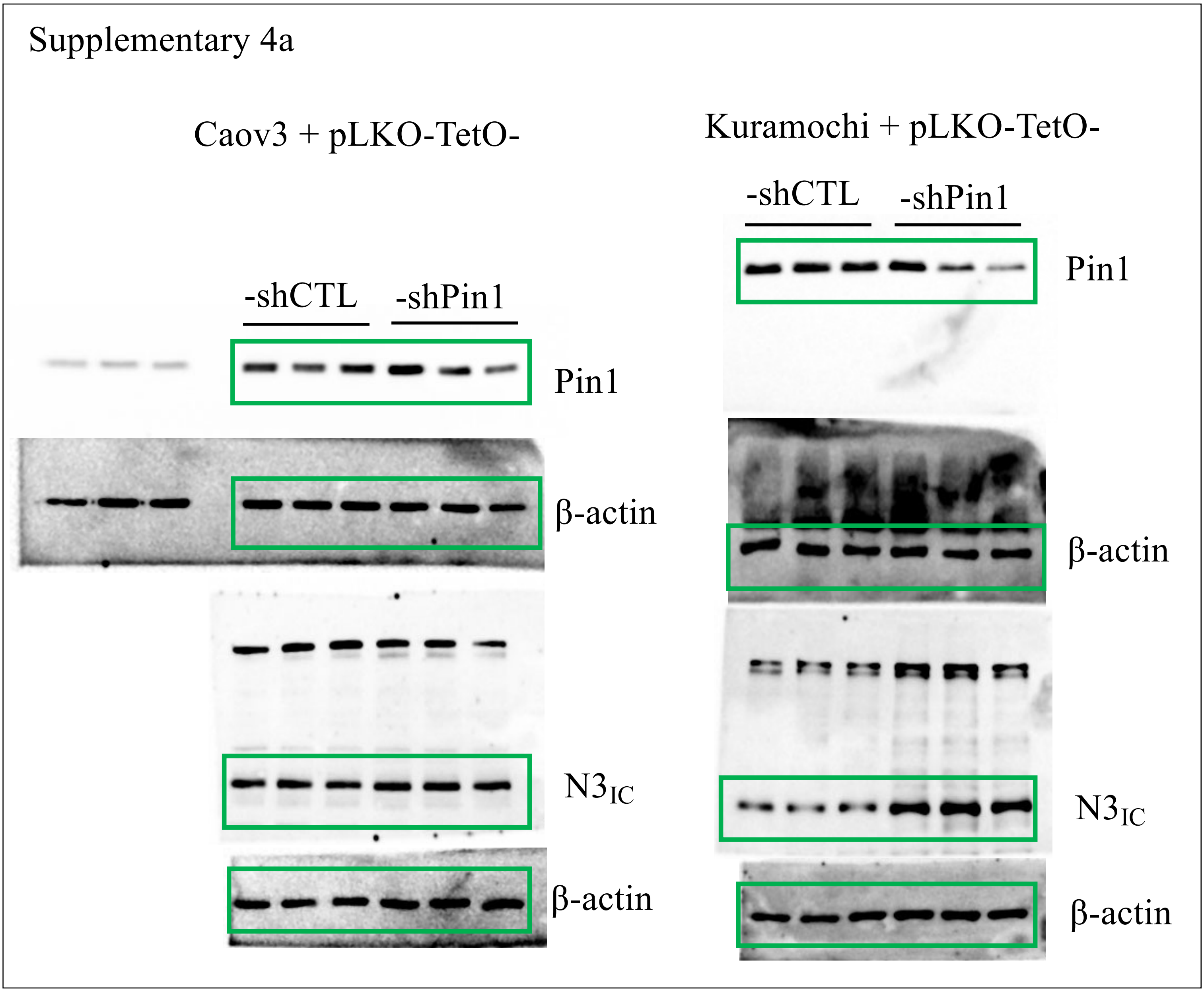

Supplementary Figure S25a. Uncropped blots and gels for the Figure S4

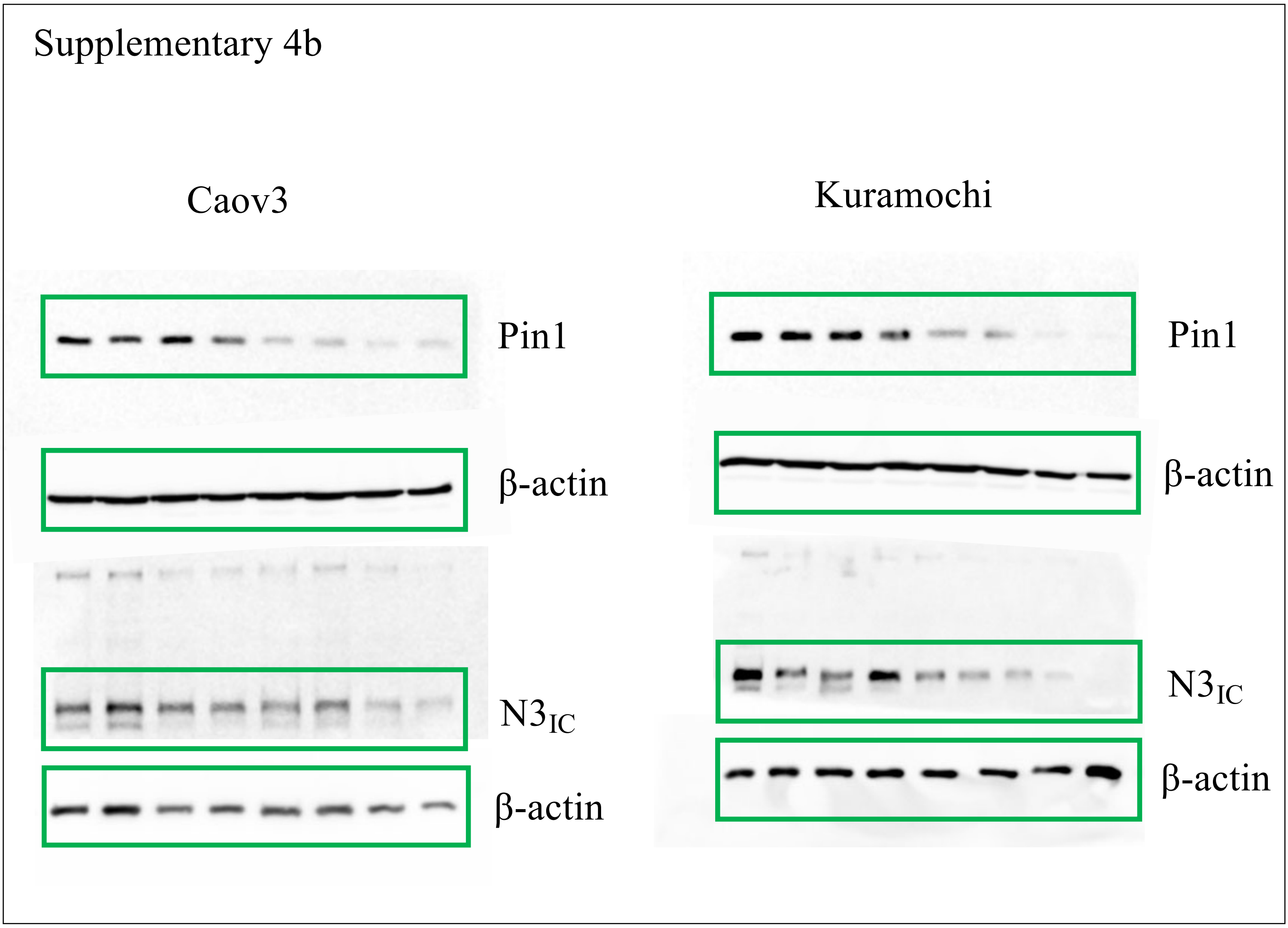

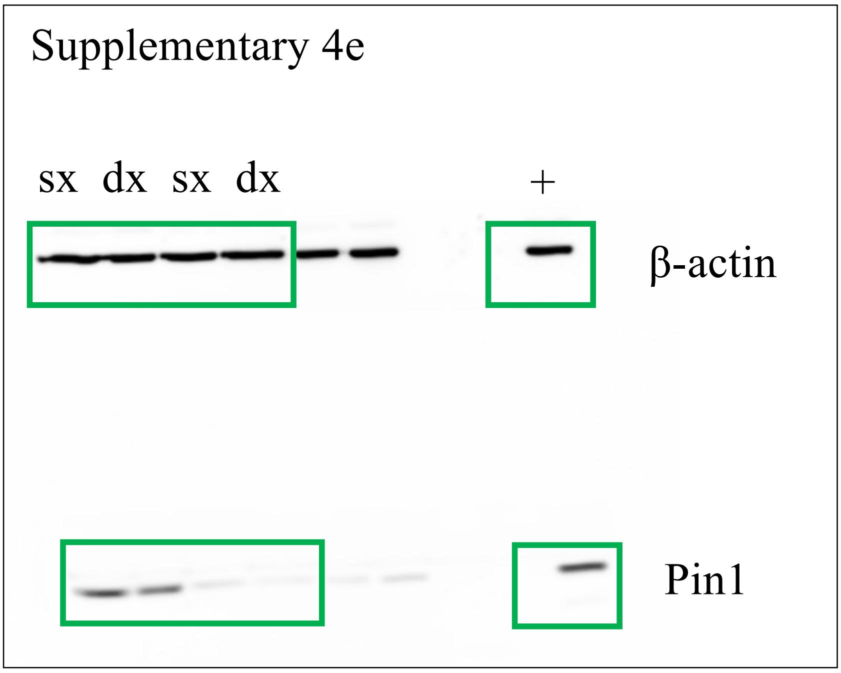

Supplementary Figure S25b. Uncropped blots and gels for the Figure S4

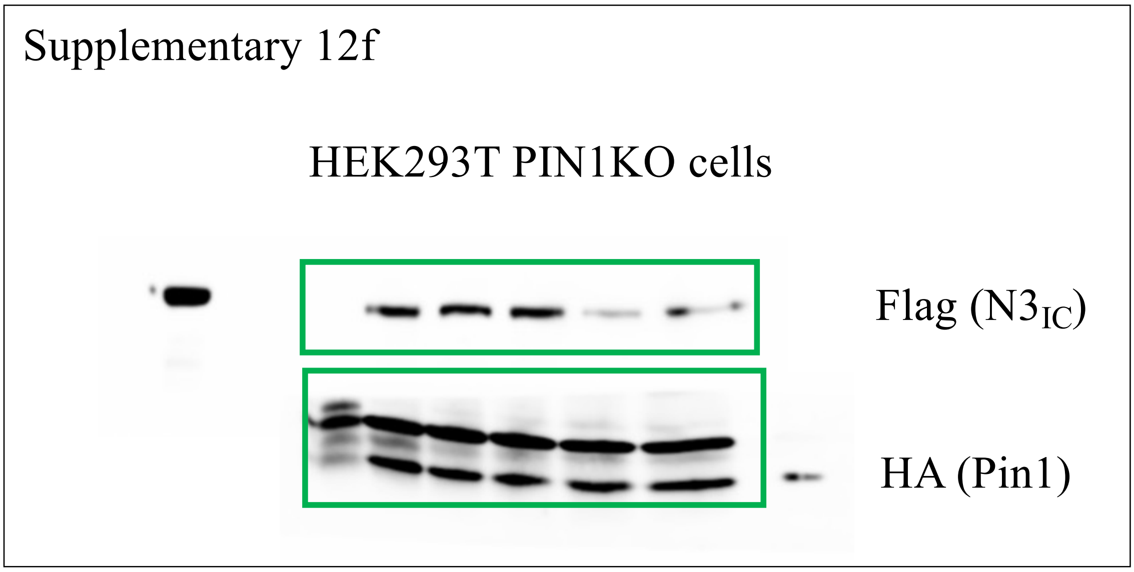

Supplementary Figure S26. Uncropped blots and gels for the Figure S12

Supplementary 14

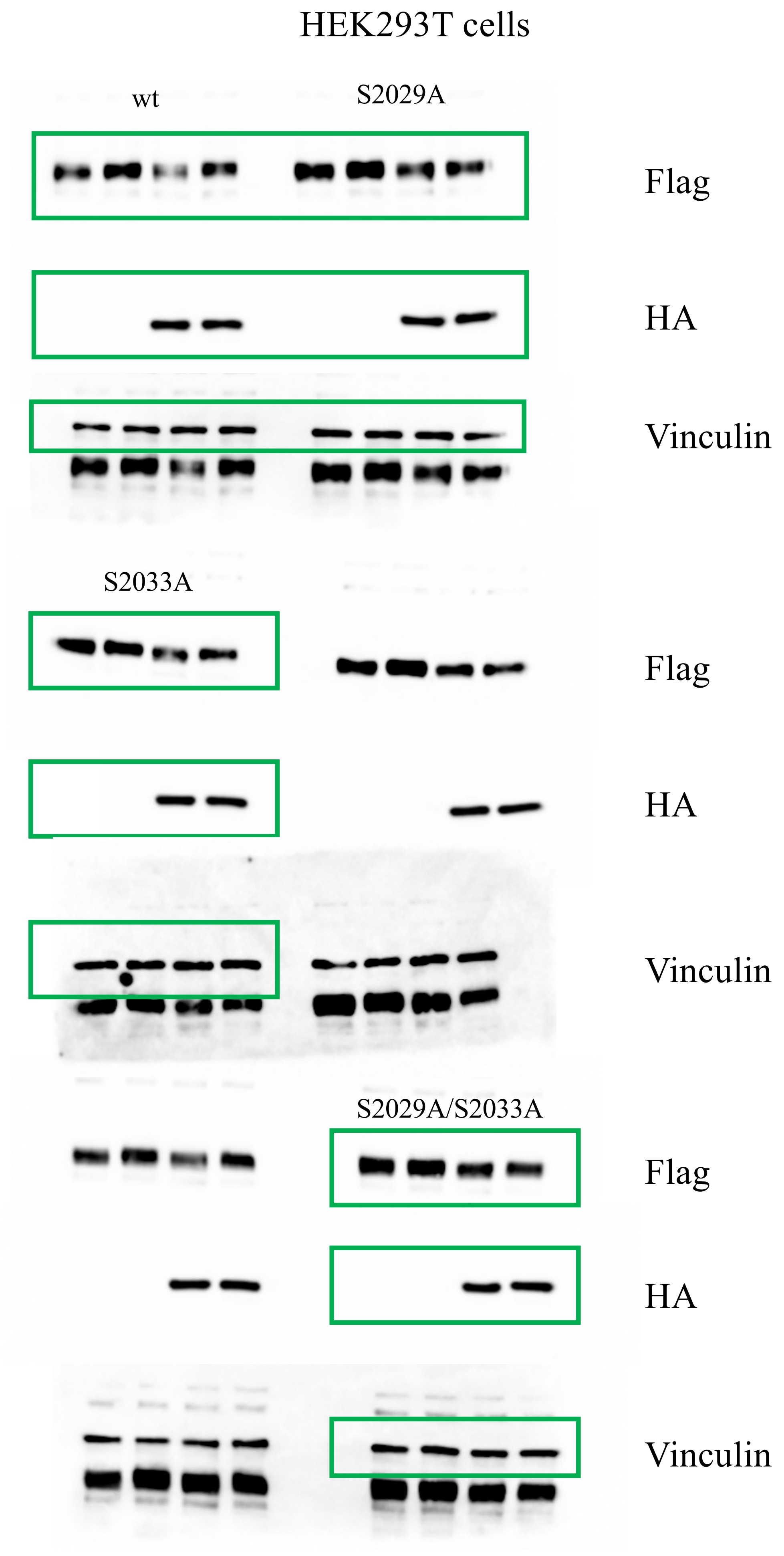

Supplementary Figure S27. Uncropped blots and gels for the Figure S14
